# Clinical and molecular genetic description of a Marfan-like syndrome in a Golden Retriever puppy

**DOI:** 10.1093/jvimsj/aalag222

**Published:** 2026-09-30

**Authors:** Harrison T Lennertz, Megan E McClosky, Maria del Mar Fuentes, Jessica K Niggel, Margret L Casal, Leonardo Murgiano

**Affiliations:** School of Veterinary Medicine, University of Pennsylvania, Philadelphia, PA 19104, United States; Department of Clinical Sciences & Advanced Medicine, School of Veterinary Medicine, University of Pennsylvania, Philadelphia, PA 19104, United States; School of Veterinary Medicine, University of Pennsylvania, Philadelphia, PA 19104, United States; Sylvia M. Van Sloun Laboratory for Canine Genomic Analysis, University of Pennsylvania, Philadelphia, PA 19104, United States; Department of Clinical Sciences & Advanced Medicine, School of Veterinary Medicine, University of Pennsylvania, Philadelphia, PA 19104, United States; Sylvia M. Van Sloun Laboratory for Canine Genomic Analysis, University of Pennsylvania, Philadelphia, PA 19104, United States; Division of Experimental Retinal Therapies, Department of Clinical Sciences & Advanced Medicine, School of Veterinary Medicine, University of Pennsylvania, Philadelphia, PA 19104, United States

**Keywords:** cardiac enlargement, genetic analysis, in-frame deletion, Marfan syndrome

## Abstract

**Background:**

Marfan syndrome (MFS) is a genetic fibrillin disorder causing cardiac, ocular, and musculoskeletal pathology; it is not well reported on in dogs, with minimal descriptions of antemortem diagnosis.

**Hypothesis/Objectives:**

Use whole-genome sequencing (WGS) to diagnose a dog with suspected MFS to guide prognostication and potential treatment.

**Animals:**

A client-owned, 10-month-old Golden Retriever presented with signs of possible connective tissue disorder. Blood and DNA samples from a non-affected German Shepherd-Beagle mix and 160 unrelated Golden Retrievers were used as controls.

**Methods:**

Physical examination, blood collection, and radiography were performed on the dog while at the hospital. Whole-genome sequencing was performed on DNA from the affected dog. Genetic analysis focused on single-nucleotide variants, small insertion–deletions, and structural variants to detect potential disease-causing genetic variants in the genome, prioritizing candidate genes. Polymerase chain reaction amplification and Sanger sequencing of a suspected region were carried out to confirm presence of the candidate pathogenic variant.

**Results:**

The dog was presented for cardiology consultation and had clinical signs of cardiac enlargement, pneumothorax, and joint hypermobility. After genetic analysis, a 3618 base pair deletion encompassing exon 24 of the *FBN1* gene on 1 allele was detected (NC_049251.1:g.14998228_15001846del), likely causing an in-frame deletion in the coding sequence.

**Conclusions and clinical importance:**

Physical and complementary examination findings are suggestive of MFS and associated with a causal deletion variant.

## Introduction

Marfan syndrome (MFS) is an autosomal dominant connective tissue disorder caused by genetic variants in the *FBN1* gene, which encodes fibrillin-1.^1^ Common clinical signs associated with the disease include aortic root dilatation or dissection and ectopia lentis, with additional systemic features including joint hypermobility, elongated appendicular skeleton, and deformities in the thorax.^2^ Although most thoroughly characterized in humans, spontaneous cases of MFS have been reported in cattle.^3^ Experimental models also have been created in mice,^4^ pigs,^5^ zebrafish,^6^ and rabbits,^7^ using genetic manipulation to yield organisms heterozygous for the pathogenic *FBN1* allele. Marfan syndrome and Marfan-like diseases also have been reported in dogs with variable degrees of confirmation, often relying on histological^8^^,^^9^ and physiological^10–12^ observations, but the identification of a pathogenic *FBN1* variant associated with the disorder has yet to be reported in dogs.

Whole-genome sequencing (WGS) has been successfully adopted in clinical settings.^13^ Although sequencing costs continue to decrease, data analysis can still be expensive.^14^^,^^15^ Targeted methods offer less expensive, streamlined results that may work well for detecting small variants in specific genes but can miss larger or heterozygous deletions.^16^^,^^17^ Whole-genome sequencing remains the most comprehensive approach, capturing both small and large intronic and exonic variants. When used in combination with genetic variant databases, its broad data output supports hypothesis-driven analysis and, despite higher initial costs, can decrease the need for multiple individual gene tests.^18^

A dog was presented to the Ryan Veterinary Hospital at the University of Pennsylvania for further evaluation of clinical signs of cardiac disease. Examination findings raised concerns for a possible connective tissue disorder, including MFS. We utilized WGS of the dog’s DNA to arrive at a genetic diagnosis.

## Materials and methods

### Animals

A 10-month-old male Golden Retriever dog was presented for a cardiology consultation regarding a previously detected heart murmur and possible signs of cardiac enlargement. Blood from a clinically normal German Shepherd-Beagle mix and DNA from 7 randomly selected, clinically normal Golden Retrievers previously tested for congenital ichthyosis (prebreeding screening) were used as controls. In addition, DNA from 153 Golden Retrievers from the United States of America (as was the proband) with no reported clinical signs similar to those of our dog was used as an additional control.

### Examination methods

Thorough physical examination was performed on the dog and a history was obtained. Additional diagnostic tests included a CBC (Advia 2120i, Siemens), biochemical profile (Vitros 4600, Ortho Quidel), radiography (Precision 600FP, GE Healthcare), echocardiography (EPIC CVx, Philips; Philips S9-2 Phased Array Transducer), and computed tomography (Revolution Maxima, GE Healthcare).

### DNA extraction

Genomic DNA was isolated from EDTA blood samples of the proband and controls using the Gentra Puregene Blood Kit (QIAGEN, Germantown, MD) according to the manufacturer’s protocol.

### Ethical statement

This work involved animals for which consent was obtained for experimental use of collected blood samples that would otherwise have been discarded. Established internationally recognized high standards of individual veterinary patient care were followed.

### Data availability

The whole-genome data of the proband has been added to the Dryad database: https://doi.org/10.5061/dryad.fn2z34v7b.

### Whole-genome sequencing

DNA from the dog was used to prepare a PCR-free library as follows: libraries of 300 base pair (bp) insert size were prepared and sequenced on Illumina NovaSeq 6000 (paired-end reads, 2 × 100 bp); FASTQ files were generated using Casava (1.8). Approximately 642 million reads (100 bp paired-end reads) were collected and successfully mapped for the sequenced dogs, corresponding to an approximate average of 27.5× coverage of the genome for the case. The paired-end reads were mapped against the dog reference genome UU_Cfam_GSD_1.0 (canFam4, BioProject PRJNA587469)^19^^,^^20^; the binary alignment map (BAM) file was processed, and the variant calling, alignment, and visualization were performed as previously described.^21^

### SNV and short indel detection

Variant calling was carried out using GATK (version 4.2.3.0)^22^ in the “HaplotypeCaller” mode, with the output set to variant call format (VCF v.4.0); raw call processing was carried out as previously described.^21^ Using SnpEff,^23^ the projected impacts of detected variants were determined. Single nucleotide variants (SNVs) and small insertion–deletion (indel) candidate variants called from the whole-genome alignment were filtered against the Dog10K.^19^^,^^20^ Further searching was carried out using the European Variation Archive variant browser (https://www.ebi.ac.uk/eva/?Variant-Browser, accessed on July 14, 2025).

### Structural variants discovery

Delly2^24^ was used for duplications, inversions, insertions, and deletions. An initial batch of 18 dogs of various breeds, including the proband, was called simultaneously. Further exclusion of variants was carried out using other controls and consulting BAM files from unrelated dogs belonging to other studies carried out by our group. Functional candidates then were checked if present in the Dog10K database (specifically in https://kiddlabshare.med.umich.edu/dog10K/Manta-SV_2021-11-18/SV-genotype.merge.vcf.gz, accessed on July 14, 2025—1879 dogs).^19^^,^^20^ The commands for deletions, insertions, inversion, and duplications were all executed separately. The variants generated were filtered against a bed file obtained from canFam4.gtf downloaded from University of California Santa Cruz (UCSC) Table Browser (https://genome.ucsc.edu/cgi-bin/hgTables, accessed July 14, 2025) to prioritize variants falling within genes.

### Genomic analysis

Spreadsheets containing information regarding SNVs, indels, and structural variants found in the genome were manually reviewed for variants located in genes of interest correlating to the observed phenotype, utilizing resources such as the National Library of Medicine and Online Mendelian Inheritance in Man (OMIM). The genome was visualized using IGV,^25^^,^^26^ with reads mapped to the UU_Cfam_GSD_1.0 reference genome.

### FBN1 amplification and screening

The dog’s *FBN1* gene was amplified from genomic DNA using PCR. The reaction was performed according to the suggested parameters for Thermo Scientific *Taq* DNA Polymerase #EP0404 (see Supplementary File 1). Both forward primers were designed relative to the same reverse primer, Forward 1, targeting upstream of the deletion, and Forward 2, targeting within the location of the deletion (Table 1).

**Table 1:** Primer sequences and base positions in CFA30 along the UU_Cfam_GSD_1.0 reference genome used for deletion detection via PCR.

**Primer**	**Sequence**	**Position start**	**Position end**
**Forward 1**	TCAGGGGAGATTCGGCATTC	14 997 883	14 997 902
**Forward 2**	TGAAGCCTTCTGGGCAAACT	15 001 053	15 001 072
**Reverse**	TAGCCTTCCTCCTCAAGCTC	15 002 033	15 002 014

The PCR products containing only the Forward 1 primer product from the deleted allele were sent to the University of Pennsylvania DNA Sequencing Facility for Sanger sequencing. Received sequences were aligned to the *FBN1* gene of the UU_Cfam_GSD_1.0 reference genome using ClustalW. BLAST-like alignment tool (BLAT) was utilized to determine the exact deletion boundaries.

## Results

### Clinical findings

A 10-month-old male Golden Retriever was referred for cardiology consultation. No familial history was available. Two months before presentation, the dog was evaluated by the primary veterinarian for vomiting, diarrhea, and decreased appetite. A heart murmur was auscultated at that visit. At presentation, the dog also had a 2-month history of weight loss and exercise intolerance. Six months before, the dog sustained a fracture of the right carpus and phalanges with no known traumatic incident.

On physical examination, the dog’s vital signs were within normal limits. It was underconditioned with a body condition score of 3/9. The dog had bilateral corneal scarring. Lung sounds were subjectively quiet in the right hemithorax. A grade IV/VI left-sided systolic murmur was auscultated with strong and synchronous femoral pulses. Multiple joints displayed hypermobility characterized by hyperflexion of the right carpus and hyperextension of the right tarsus.

Complete blood count identified mild, mature neutrophilia. The serum biochemical profile was within normal limits. Three-view thoracic radiographs identified marked hyperinflation of a right lung lobe, causing a mass effect with leftward mediastinal shift consistent with congenital lobar emphysema (Figure 1). Echocardiography identified a markedly enlarged aortic annulus and aortic root. Annuloaortic ectasia secondary to a congenital connective tissue disorder was suspected. Computed tomography of the thorax was performed and confirmed the presence of right middle lung lobe emphysema causing displacement of the right caudal and accessory lung lobes (Supplementary File 2). Thoracotomy with right lung lobectomy was initially considered for treatment, but these scans identified abnormalities in both lung lobes that, in addition to the cardiac findings, made the dog a poor candidate for anesthesia, and surgery therefore was not performed.

**Figure 1:**
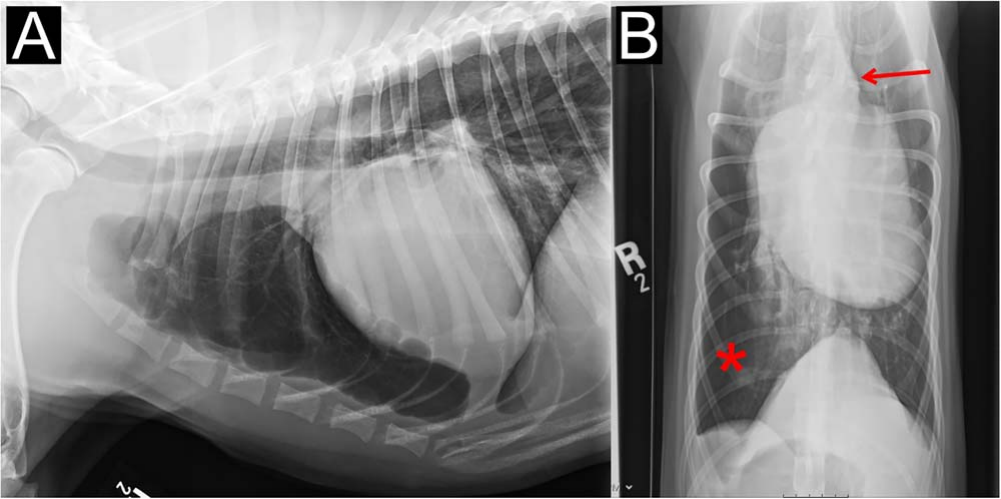
Radiographic views of the affected dog’s thorax. **(**A) Right lateral view of the region, (B) ventral-dorsal view. Left-sided cardiomegaly, aortic dilatation (marked by arrow), and right lung hyperinflation (marked by asterisk) can be observed.

### Candidate variant detection

Because we only had access to a single case with no related dogs accessible, we opted to proceed with WGS of the dog and prioritize plausible candidate genes. The reads were mapped against the UU_Cfam_GSD_1.0 reference (canFam4). The called variants were filtered against the Dog10K database and an internally generated database of sequenced dogs. Filtering assumed a dominant inheritance mechanism.

After the filtering process, 1144 SNV and small indel variants remained as potential candidates. Of these, 1135 were non-coding; 9 were coding. After filtering associated SNVs or small indel variants, we next assessed the presence of large deletions, insertions, inversions, and duplications, of which 252 were detected in total, including 176 deletions, 31 insertions, 20 duplications, and 25 inversions. The variants were filtered against an internal database of dogs without the candidate variant generated by our laboratory for other studies, and the UU_Cfam_GSD_1.0 (canFam4) Dog10K database (see Supplementary File 3). None of the filtered SNVs or small indel variants were deemed suitable after manual review indicated minimal or no impact of potential genetic variants on the presenting phenotype.

### Heterozygous, mosaic deletion of FBN1 exon 24

A 3618 bp deletion in 1 *FBN1* allele was detected via Integrative Genomics Viewer (IGV), as evidenced by a marked decrease in read frequency, and Delly2, with the deletion encompassing the entirety of exon 24 in the gene (Figure 2A). The deletion causes an in-frame deletion which, barring additional processing, only removes exon 24 from the mRNA transcript (228 bp) as well as the *FBN1* and fibrillin-1 precursor resultant amino acid sequence (see Supplementary File 4).

**Figure 2:**
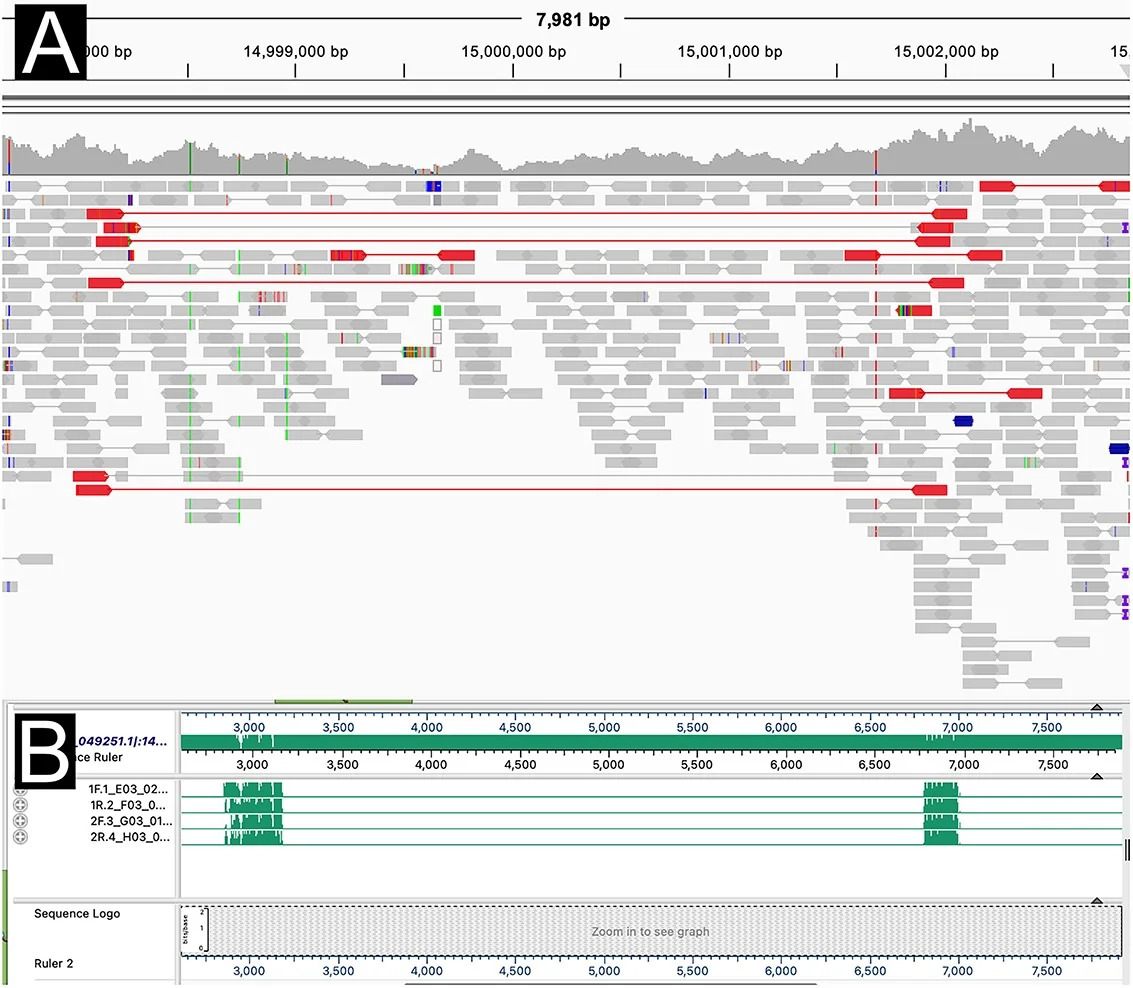
*FBN1* deletion of exon 24 in the dog. A is the region of the whole-genome sequence viewed as pairs on IGV. B is the PCR product of the forward 1 deletion amplification mapped to the reference genome, along the same region of *FBN1*, visualized using MegAlign pro. The latter image provides more clarity for the precise ends of the deletion.

After further inspection of the WGS data, Sanger sequencing suggested that the deletion was mosaic. Specifically, within the deleted interval, reads from the intact haplotype appeared to include a low-frequency second haplotype, consistent with a non-deleted allele persisting in a subset of cells and hence the uneven occurrence of SNVs within the deleted region (Supplementary File 5). This finding also suggests uneven heterozygosity of the deletion (comprising fewer than 50% of the total *FBN1* alleles).

### PCR amplification and deletion boundaries

After detection of the deletion, the *FBN1* gene was amplified from the respective genomic DNA of the affected dog and German Shepherd-Beagle (see *FBN1* Amplification and Sequencing, Figure 3A). Sequencing was performed on the Forward 1 deletion PCR product, which, when aligned to the reference genome, confirmed a 3618 bp gap (NC_049251.1:g.14998228_15001846del, Figure 2B, Supplementary File 6). Polymerase chain reaction was also performed on 160 additional DNA samples of non-affected Golden Retrievers, none of which showed presence of the deletion (Figure 3B, Supplementary File 7).

**Figure 3:**
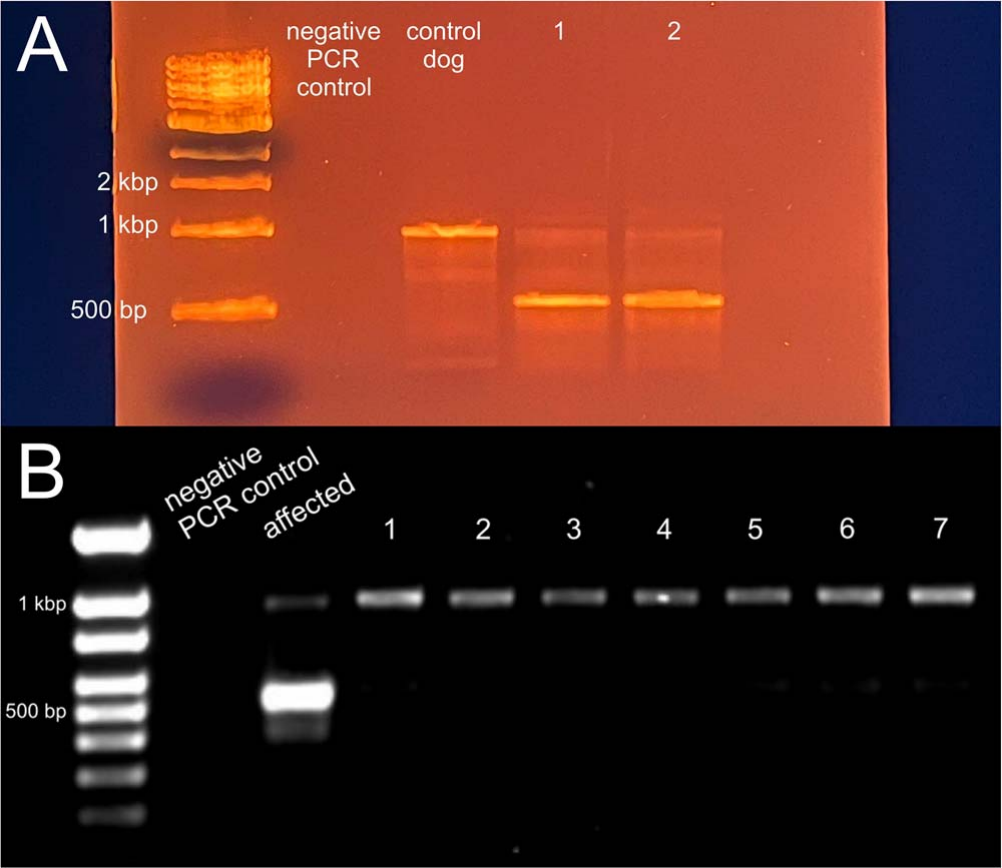
PCR for deletion detection and confirmation of unique *FBN1* variant. (A) PCR amplification of the area surrounding the exon 24 deletion in *FBN1*. The approximately 1 kbp band is a product of the forward 2 primer, confirming presence of a wild-type allele, and the approximately 500 bp band is a product of the forward 1 primer containing the deletion. Lanes 1 and 2 are both derived from 2 different extractions of the affected dog’s genomic DNA. (B) The affected sample is the same DNA as lane “1” in Figure 3A, with the same primers used. Lanes labeled 1-7 each contain PCR products of unique DNA samples of non-affected golden retrievers. The deletion (approximately 500 bp) band is only clearly seen in the affected sample.

## Discussion

We suspect the cause of the dog’s presumptive MFS to be the deletion in the *FBN1* gene because of its occurrence in a coding region and its association with similar clinical signs as observed in patients across species. Intriguingly, WGS and Sanger sequencing data suggest that a subset of cells of the proband does not carry the deletion, consistent with localized mosaicism. The observed pattern is compatible with a post-zygotic mosaic deletion, potentially arising during early development. This finding cannot be observed by the PCR band migration pattern alone because the non-deleted mosaic haplotype would co-migrate with the intact allele. The deletion was not detected in 160 randomly selected, non-affected Golden Retrievers, further supporting the uniqueness of the deletion in this dog. For additional confirmation of gene homology, a nucleotide BLAST of the reference *FBN1* sequence (NC_049251.1; 14 925 119-15 148 304) yielded 100% identity matches with multiple homologs within the *Canis lupus familiaris* species, with the best result being accession NM_001287085.2.

The deletion found in the dog of our report is consistent with previous diagnoses of MFS in humans, suggesting a similar pathophysiology. There are numerous reported disease-associated variants within *FBN1* in humans, all occurring within variable regions of the gene.^27–29^ They often affect cysteine residues or cause large-scale defects in the protein, with particularly pathogenic variants often being identified in exons 24-32.^28^^,^^30^^,^^31^ In our case, there was a deletion of the entirety of exon 24, a transforming growth factor-β (TGF-β) binding protein-like domain,^32^ as well as its surrounding intronic regions. This variant occurred in only 1 allele, but the autosomal dominant inheritance pattern of MFS suggests this occurrence would be sufficient genetic basis for disease. Possible pathologic mechanisms of this in-frame deletion could be inhibition of heparin binding,^33^ upregulation of TGF-β expression, or creation of a truncated product that would prevent proper formation of microfibrils.^27^ A previous characterization of exon 24 being skipped in a human patient yielded clinical signs of classical MFS.^30^ Furthermore, an in-frame deletion of exons 24-26 was found in a newborn presented with classical neonatal MFS, with more extreme clinical signs of pneumothorax and cardiomegaly^34^ compared with the previously mentioned human patient. Both findings, given their similarity to our case, highly suggest that the absence of exon 24 is causative of our dog’s MFS. However, because no biopsies were performed, we are unable to consider post-transcriptional or post-translational modifications, including possible impact on expression by the deleted intronic regions, as well as the influence of nonsense-mediated decay.^35^ These mechanisms might mitigate the expression of the aberrant allele, and dominant interference pathways could degrade polymers formed with affected monomers, attributing the diseased phenotype to haploinsufficiency of fibrillin-1.

Whole-genome sequencing has not been used previously to detect abnormalities in the *FBN1* gene in a dog,^36^ and this approach better reflects modern standards of diagnosis and allows for proactive treatment of MFS. Proper diagnosis of MFS has become reliant on genetic testing, with most criteria involving identification of a disease-causing variant in *FBN1.*^1^ Pathogenic variants in this gene are associated with many outcomes such as marfanoid features not including aortic dilatation or ectopia lentis, insufficient severity of aortic dilatation or ectopia lentis, or an entirely different presentation such as Weill-Marchesani syndrome. Genetic analysis helps eliminate confusion between MFS and other similarly presenting connective tissue disorders. Many previously reported diagnoses of MFS in dogs might not satisfy the current requirements for diagnosis because several were dependent on abnormal physiology, particularly regarding cardiac signs.^11^ Given a lack of genetic testing, further investigations involved analyzing autopsied heart and vascular tissue to visualize fibrillin-related abnormalities^8^^,^^9^ or relative expression of *FBN1* across tissue types.^12^ Genetic testing provides a more clear cause for suspected cases, prevents confusion with other similarly presenting connective tissue disorders such as cardiac-valvular Ehlers-Danlos syndrome^37^^,^^38^ or Loeys-Dietz syndrome,^1^^,^^29^ and allows for confident diagnosis while the patient is living. It also allows for better prognostication and may alert the clinician to associated internal findings that may not be noted on physical examination, such as aortic root dilatation.

Our findings support previous instances of WGS being used as a diagnostic tool.^39–42^ Whole-genome sequencing, in conjunction with structural variants analysis, allowed a broad analysis of the genome to explore many variants of interest, including intronic and non-coding variants that could affect expression, and created a resource that can be used for further reference should concerns of additional congenital disease arise. More limited forms of genetic analysis might not have been able to detect this variant given its size and heterozygosity, and although techniques such as multiplex ligation-dependent probe amplification might be capable of detecting deletions of this size,^43^ it still would require considerable suspicion of *FBN1* as the gene of interest. Whole-genome sequencing generates a wealth of information, but allows for hypothesis-driven analysis and decreases the amount of testing potentially needed given negative initial results. Although it is not necessarily the most inexpensive method for genetic analysis, it is still much less expensive than other physiological diagnostic tools, such as magnetic resonance imaging.

We were unable to reach the dog’s owners to obtain information about the proband’s parents or littermates despite multiple attempts, which represents a considerable study limitation for tracing the potential inheritance of the variant allele. Given our analysis to suggest its mosaic nature, however, we believe it arose de novo. This hypothesis suggests that the deletion of interest is most likely not present in any parental alleles.

Additionally, the deletion was detected using a number of methods: visual observation using IGV, confirmation by PCR, and programs for detecting large deletions, such as DELLY^24^ and ExomeDepth,^29^ which might serve as an initial step for future similar cases.

In conclusion, we explored an instance of deletion of exon 24 in a *FBN1* allele from a dog correlated with clinical signs such as aortic dilatation, components that would meet the baseline requirements for a diagnosis of MFS as defined in humans.^1^ This case hopefully will serve as a model to understand how MFS presents in dogs and further support the use of WGS as a useful initial diagnostic technique for suspected congenital diseases, especially in veterinary medicine.

## Supplementary Material

Supplementary_material_aalag222

## Off-label antimicrobial declaration

Authors declare no off-label use of antimicrobials.

## Institutional animal care and use committee or other approval declaration

Authors declare no institutional animal care and use committee or other approval was needed.

## Abbreviations

indel: insertion–deletion
MFS: Marfan syndrome
PCR: polymerase chain reaction
SNV: single-nucleotide variant
WGS: whole-genome sequencing
