# Supplementary material for: Clinical and molecular genetic description of a Marfan-like syndrome in a Golden Retriever puppy
Source: J Vet Intern Med. 2026 Sep 30;40(5):aalag222. doi: 10.1093/jvimsj/aalag222 (PMC13626598; doi:10.1093/jvimsj/aalag222)
Supplement: Supplementary file 1 [file supplementary_material_aalag222.zip › File S_4.docx]

**Supplementary File 3A:** Transcript of predicted *FBN1* mRNA transcript (NM_001287085.2) with deleted region highlighted. NM_001287085.2:c.A3,042_3,269del

TGCAGCTGGCAGGGGCCGCCTGAAGCGGGAGCAGCGCCTGGAGCAGGCGGGAGGAGCCGGCCCGGGGGAC

GGGCGGCGGGACAGCGGGACCCCGGCGGCGCGGCGCGCTTCGGGGCGCAGCGGCGGCCGCGGATCGAGCC

CGGGGCGCGGCGAGAGGCGGCAGGAGCCGGCGGCCGCTCGGCATCATGCGGCGGGGAGGGCTGCTGGAGG

TCGTCCTGGGATTTACCGTGCTCTTAGCGTCCTACACGAGCCACGGGGCGGACGCCAATTTGGAGGCTGG

GAACGTGAAGGAGACCAGGGCCAGCCGGGCCAAGAGAAGAGGCGGCGGGGGACACGACGCGCTTAAAGGA

CCCAATGTCTGTGGATCACGTTATAATGCTTACTGTTGCCCTGGATGGAAAACCTTACCTGGTGGAAATC

AATGTATTGTCCCCATTTGCCGGCATTCCTGTGGGGATGGATTTTGTTCTAGGCCAAATATGTGCACTTG

CCCATCTGGTCAGATATCTCCTTCCTGTGGCTCCAGATCCATCCAACACTGTAATATTCGCTGTATGAAC

GGAGGTAGCTGTAGTGATGATCACTGTCTGTGCCAGAAAGGATACATAGGGACTCACTGCGGACAACCTG

TCTGTGAAAGTGGCTGTCTCAATGGAGGCAGGTGTGTGGCTCCGAATCGATGTGCCTGCACATACGGCTT

TACTGGACCCCAGTGTGAAAGAGATTACAGGACAGGCCCATGTTTTACTGTGGTCAGCAACCAGATGTGC

CAGGGACAGCTCAGCGGGATTGTCTGCACAAAAACACTCTGCTGTGCCACGGTGGGCAGAGCCTGGGGCC

ACCCGTGCGAGATGTGCCCCGCCCAGCCCCACCCCTGTCGCCGAGGCTTCATTCCAAATATCCGTACGGG

AGCCTGTCAAGATGTGGATGAATGCCAGGCCATCCCTGGGCTCTGTCAAGGAGGAAATTGCATTAATACT

GTTGGGTCTTTTGAGTGCAAATGCCCTGCTGGACACAAATTTAATGAAGCATCACAAAAATGTGAAGATA

TTGACGAATGCAGTACCATTCCTGGAATCTGTGATGGGGGCGAGTGTACAAACACAGTCAGCAGTTACTT

TTGCAAGTGTCCTCCTGGTTTTTACCCCTCTCCCGATGGTACCCGATGCGTAGATGTTCGCCCCGGATAC

TGTTACACAGCTCTGGCAAATGGGCGCTGTTCTAACCAACTGCCCCAGTCCATAACAAAAATGCAGTGCT

GCTGTGATGTTGGCCGATGCTGGTCTCCAGGGGTCACCGTTGCCCCTGAGATGTGTCCCATCAGAGCAAC

AGAGGATTTCAACAAGCTGTGCTCTGTCCCCTTGGTAATTCCTGAGAGACCAGGATATCCTCCCCCACCC

CTTGGCCCCATTCCTCCAGTTCACCCTGTTTCTCCTGGCTTTCCTCCTGGACCTCCAGTTCCAGTCCCTC

GACCACCAGTGGAATATCCATACCCGTCTCGGGAACCACCAAGGGTGCTGCCAGTCAATGTCACTGATTA

CTGCCAGCTGTTTCGATATCTCTGTCAAAATGGACGCTGCATTCCAACTCCTGGGAGCTATCGGTGTGAA

TGCAACAAAGGATTCCAGCTGGACCTTCGTGGGGAGTGCATTGATGTTGATGAATGTGAGAAGAACCCTT

GTGCTGGTGGAGAGTGTATTAATAACCAGGGCTCGTACACCTGTCAGTGCCGACCTGGCTATCAAAGCAC

ACTCACCCGGACAGAGTGCCGAGACATCGACGAATGTTTACAGAATGGCCGTATCTGCAATAATGGGCGT

TGCATTAACACAGATGGCAGTTTCCACTGTGTGTGTAATGCAGGCTTTCATGTGACACGAGATGGGAAGA

ACTGTGAAGATATGGATGAATGCAGCATAAGGAACATGTGCCTCAATGGAATGTGTATCAATGAAGATGG

CAGTTTTAAATGTATTTGCAAACCTGGATTTCAGCTGGCATCAGACGGGCGTTATTGCAAAGATATTAAT

GAGTGTGAAACACCTGGGATCTGCATGAATGGACGCTGTGTCAACACTGATGGCTCCTACAGATGTGAAT

GCTTTCCTGGACTGGCTGTGGGTCTCGATGGTCGTGTGTGTGTTGACACACACATGCGGAGCACATGCTA

TGGTGGATATAAGAGAGGCCAGTGTGTCAAACCGTTGTTTGGTGCCGTTACTAAATCTGAATGCTGTTGT

GCCAGCACTGAGTATGCTTTTGGGGAACCTTGCCAACCATGTCCTGCACAAAATTCAGCGGAATATCAGG

CACTCTGCAGCAGTGGGCCAGGAATGACATCAGCAGGCAGTGATATAAATGAATGTGCGTTAGATCCGGA

TATCTGTCCAAACGGAATTTGTGAAAATCTCCGTGGGACCTACAAATGCATATGCAATTCAGGATATGAA

GTGGATCCATCTGGGAAGAACTGTGTTGATATTAATGAATGTGTATTGAACAGTCTGCTTTGTGATAATG

GACAATGTAGAAACACTCCTGGAAGTTTTGTCTGTACCTGTCCCAAGGGATTTATCTACAAACCTGACCT

AAAAACCTGTGAAGACATCGACGAATGTGAATCAAGTCCTTGCATTAATGGAGTCTGCAAGAACAGCCCG

GGCTCTTTTATTTGTGAATGTTCTTCCGAAAGTACCCTGGATCCAACAAAAACCATCTGCATAGAAACCA

TCAAGGGCACTTGCTGGCAGACTGTCATCGATGGGCGGTGTGAGATCAACATCAATGGAGCTACCTTAAA

GTCCCAGTGCTGTTCCTCTCTTGGTGCTGCCTGGGGGAGCCCTTGTACCCTATGCCAAGTCGATCCCATA

TGTGGCAAGGGCTACTCAAGAATTAAAGGAACACAGTGTGAAGATATAAATGAGTGTGAAGTATTTCCAG

GAGTATGCAAAAATGGTGTATGTGTTAACTCTGAGGGATCATTTAAATGTCAGTGTCCCAATGGGATGAC

TTTGGATGCCACAGGAAGGATCTGCCTAGATATCCGCCTGGAAACCTGCTTCCTGAGGTATGAGGATGAG

GAATGCACTCTGCCCGTTGCTGGCCGCCACCGCATGGACGCCTGTTGCTGCTCCGTTGGGGCAGCCTGGG

GTACTGAGGAATGCGAGGAGTGTCCTGTGAGAAACACGCCTGAGTACGAGGAGCTCTGTCCCAGAGGGCC

CGGATTCGCCACAAAAGAAATTACCAATGGAAAACCTTTCTTCAAAGATATCAACGAATGCAAGATGATT

CCCAGCCTCTGTACCCACGGCAAATGCAGAAACACCATTGGCAGCTTTAAGTGCAGGTGTGACAGTGGCT

TTGCTCTTGACTCTGAAGAAAGGAATTGCACAGACATCGATGAATGCCGAATCTCCCCTGACCTCTGCGG

CCGAGGCCAGTGCGTGAACACCCCAGGGGACTTCGAATGCAAGTGTGACGAAGGCTATGAAAGCGGATTC

ATGATGATGAAGAACTGCATGGATATCGATGAGTGTCAGAGAGATCCTCTCCTGTGCCGGGGTGGCGTTT

GCCTCAACACTGAGGGAAGTTACCGCTGTGAGTGTCCTCCTGGTCATCAGCTCTCTCCCAACATCTCAGC

ATGTATAGACATCAACGAGTGTGAACTGAGTGCGAACCTCTGCCCTAATGGCCGTTGTGTGAACCTCATC

GGGAAGTATCAGTGTGCTTGCAACCCTGGCTATCATTCAACTCCTGACAGGCTGTTTTGTGTGGACATCG

ACGAATGCAGCATAATGAATGGTGGTTGTGAGACCTTCTGTACAAACTCTGAAGGCAGCTATGAATGTAG

CTGTCAGCCAGGATTTGCACTAATGCCCGACCAGAGGTCATGCACTGACATTGATGAGTGTGAAGATAAC

CCCAATATCTGTGACGGCGGTCAGTGTACAAACATTCCCGGGGAGTACAGGTGCTTGTGTTATGATGGAT

TCATGGCATCTGAAGACATGAAGACTTGTGTAGATGTCAATGAATGTGACCTAAATCCAAATATCTGCCT

AAGTGGAACCTGTGAAAACACAAAAGGCTCGTTTATTTGCCACTGTGATATGGGATATTCAGGCAAAAAA

GGAAAAACTGGCTGTACAGACATCAACGAATGTGAAATTGGAGCACACAACTGTGACAGACACGCCGTAT

GTACCAACACAGCAGGAAGCTTCAAATGTAGCTGCAGCCCTGGATGGATTGGAGATGGCATTAAGTGCAC

GGATCTGGATGAATGCTCCAATGGAACACACATGTGCAGCCAACATGCAGACTGCAAGAATACCATGGGC

TCTTATCGCTGCCTCTGTAAAGAAGGATACACGGGTGATGGATTCACGTGTACAGATCTTGATGAGTGCT

CTGAGAACCTGAATCTTTGTGGCAATGGTCAGTGCCTGAATGCCCCAGGAGGATACCGCTGCGAATGTGA

CATGGGCTTTGTGCCCAGTGCTGATGGGAAAGCCTGTGAAGATATCGATGAGTGCTCCCTACCGAACATC

TGTGTTTTTGGAACTTGCCACAACCTTCCTGGCCTGTTCCGTTGTGAGTGCGAGATAGGCTACGAATTGG

ACAGAAGTGGCGGAAACTGCACAGATGTTAATGAGTGTCTGGATCCAACCACATGCATCAGTGGGAACTG

TGTCAACACTCCAGGCAGCTATACCTGTGATTGTCCTCCCGACTTTGAGTTGAACCCAACTCGAGTTGGC

TGTGTCGATACCCGTTCTGGAAATTGCTATCTGGATATTCGACCTCGAGGAGACAATGGAGATACAGCCT

GTAGCAATGAAATTGGAGTTGGTGTCTCCAAGGCTTCCTGCTGTTGTTCTTTGGGTAAAGCCTGGGGTAC

TCCTTGTGAGCTGTGCCCTCCTGTGAACACATCTGAGTATAAAATTCTCTGTCCTGGAGGAGAAGGTTTT

CGGCCCAATCCTATCACTGTCATATTGGAAGACATTGACGAGTGCCAGGAGCTCCCAGGGCTGTGCCAAG

GCGGGAAATGTATCAACACGTTTGGCAGTTTCCAATGCCGCTGTCCAACTGGTTACTACCTGAATGAAGA

CACCCGAGTGTGTGATGATGTGAATGAATGTGAAACTCCCGGAATCTGTGGTCCAGGAACATGTTACAAC

ACTGTCGGCAACTACACCTGTATCTGCCCTCCAGACTACATGCAAGTGAATGGGGGGAATAATTGCATGG

ATATGAGAAGAAGTTTGTGCTATAGAAACTACTATGCTGACAACCAAACCTGTGATGGAGAACTTTTGTT

CAATATGACCAAGAAGATGTGCTGTTGTTCCTACAACATTGGCCGGGCCTGGAATAAGCCCTGTGAACAG

TGTCCTATCCCAAGCACAGATGAATTTGCTACACTTTGTGGAAGTCAAAGGCCAGGCTTCGTCATTGACA

TTTACACCGGGTTACCTGTTGATATTGATGAATGCCGGGAGATCCCTGGGGTCTGTGAGAATGGAGTGTG

TATCAACATGGTTGGCAGCTTCAGATGTGAATGTCCGGTGGGCTTCTTCTATAATGACAAGTTGTTGGTT

TGTGAAGATATTGACGAGTGTCAGAACGGTCCTGTGTGCCAGCGCAATGCCGAGTGCATCAACACAGCAG

GCAGTTTCCGATGTGACTGCAAGCCCGGCTACCGCTTCACTTCCACAGGCCAGTGCAACGATCGCAATGA

ATGTCAAGAAATCCCCAATATTTGCAGCCATGGACAGTGTATCGACACAGTTGGAAGTTTTTATTGCCTT

TGCCACACTGGTTTTAAAACAAATGCTGATCAAACCATGTGTTTGGACATAAATGAGTGTGAGCGAGATG

CCTGTGGGAATGGAACTTGCCGCAACACGATTGGGTCCTTCAACTGCCGCTGCAACCACGGCTTCATCCT

TTCCCACAACAATGACTGCATAGATGTTGATGAATGTGCAACTGGAAATGGGAACCTTTGCAGAAATGGC

CAATGCATCAATACAGTGGGGTCCTTCCAGTGTCAGTGCAATGAAGGCTATGAGGTGGCTCCAGATGGGA

GGACCTGTGTGGATATCAATGAATGTCTTCTAGAACCTGGAAAATGTGCACCAGGCACCTGTCAAAACTT

GGATGGGTCCTACAGGTGCATTTGTCCACCTGGATACAGTCTACAGAATGACAAGTGTGAAGATATTGAT

GAGTGTGTTGAAGAGCCAGAAATCTGTGCCTTGGGAACATGCAGCAACACAGAAGGCAGCTTTAAATGTC

TCTGTCCAGATGGATTCTCCTTGTCCTCCACTGGAAGAAGGTGCCAAGATCTGCGAATGAGCTACTGCTA

TGCTAAGTTTGAAGGAGGAAAATGTTCATCACCCAAATCCAGAAATCACTCCAAGCAGGAATGCTGTTGT

GCCTTGAAGGGAGAAGGTTGGGGTGATCCCTGTGAGCTGTGCCCCACCGAACCTGATGAGGCTTTCCGCC

AGATCTGTCCCTATGGAAGTGGCATCATTGTGGGGCCTGATGATTCAGCAGTTGATATGGATGAGTGCAA

AGAACCTGATGTCTGTAAACATGGGCAGTGCATCAATACAGATGGCTCATACCGCTGCGAGTGTCCCTTT

GGTTACATTCTGGAAGGGAATGAATGTGTAGATACTGATGAATGTTCTGTGGGCAATCCTTGTGGAAATG

GAACCTGCAAGAATGTGATTGGAGGTTTTGAATGTACCTGTGAGGAGGGATTTGAGCCTGGTCCAATGAT

GACTTGTGAAGATATAAACGAATGTGCTCAGAATCCTCTGCTCTGTGCTTTCCGGTGTGTAAACACTTAT

GGGTCATATGAATGCAAATGTCCCGCTGGATATGTTCTGAGAGAAGATAGAAGGATGTGCAAAGATGAGG

ATGAGTGTGAAGAGGGCAAACACGACTGTGCTGAGAAGCAGATGGAGTGCAAGAACCTCATTGGCACATA

CATCTGCATCTGTGGGCCTGGCTATCAGCGGAGACCTGATGGAGAGGGCTGCGTAGATGAGAATGAATGT

CAGACTAAGCCGGGGATCTGTGAGAATGGGCGCTGCCTCAACACGCGGGGGAGTTACACTTGTGAATGCA

GTGACGGCTTCACCCCCAGCCCTGCCCAGGATGAGTGCCTTGACAACCGGGAAGGGTACTGCTTCACGGA

GGTGCTCCAAAACATGTGTCAGATCGGCTCAAGCAACAGGAACCCTGTCACCAAGTCTGAATGCTGCTGT

GATGGAGGGAGAGGCTGGGGCCCCCACTGTGAGATCTGTCCTTTCCAGGGCACTGTGGCCTTCAAAAAAC

TCTGCCCCCATGGCCGCGGATTTATGACCAACGGAGCAGATATTGATGAATGCAAGGTTATTCATGATGT

TTGTCGAAATGGGGAGTGTGTCAACGACAGAGGATCCTATCATTGCCTATGTAAAAATGGCTACACTCCA

GATATAACTGGAACTTCCTGTGTAGATCTGAATGAGTGCAACCAGGCCCCCAAACCCTGCAATTTTATCT

GCAAAAACACAGGAGGGAGTTTCCAGTGTTCCTGCCCGAAAGGCTACATCCTGCAGGAGGATGGAAGGAG

CTGCAAAGATCTTGATGAATGTGCCACTAAACAGCACAACTGCCAGTTCCTGTGTGTTAACACCATTGGC

AGCTTCACTTGTAAATGTCCTCCCGGATTTACACAACACCATACTGCCTGCATTGATAACAATGAATGCA

CTTCTGACATCAACTTGTGTGGGTCTAAGGGTATTTGCCAGAATACTCCAGGGAGCTTCACCTGTGAATG

CCAGCGGGGATTCTCACTTGATCAGAGTGGTGCCAGCTGTGAAGATGTGGATGAGTGTGAAGGAAACCAT

CGCTGCCAGCACGGCTGCCAGAACATCATCGGAGGCTACAGGTGCAGCTGCCCCCAGGGCTATCTCCAGC

ACTACCAATGGAACCAGTGTGTTGATGAAAACGAATGCCTCAGCGCACACATTTGTGGAGGAGCCTCCTG

TCACAACACCCTGGGAAGCTACAAGTGCATGTGCCCCGCCGGCTTCCAGTACGAACAGTTCAGTGGAGGG

TGCCAAGACATCAACGAATGTGGCTCTGCACAAGCCCCCTGCAGCTATGGATGCTCCAATACTGAGGGTG

GCTACCTGTGTGGCTGTCCACCTGGTTACTTCCGAATAGGCCAAGGGCACTGTGTTTCTGGAATTGGCAT

GGGCCGAGGAGGCTCGGAGCCACCTGCCAGTGGTGAAATGGATGACAATTCTCTCTCCCCGGAGGCTTGC

TATGAGTGTAAGATCAATGGCTACCCCAAACGAGGCAGGAAACGGAGAAGTGCAAATGAAACAGATGCCT

CCAACATTGAGGACCAGCCTGAGAGAGATACCAATGTGAGTCTTGCAAGTTGGGACACTGAGAAGACAGC

CATGTTCGCGTTCAATATTTCCCACATCAGTAACAAGGTCCGAATCCTAGAGCTCCTCCCGGCCCTGACG

ACTCTGACAAATCACAACAAATACTTGATCGAATCTGGAAATGAAAATGGCTTCTTTAAAATCAACCAAA

AGGAAGGGATCAGCTACCTCCACTTCACCAAGAAGAAGCCAGTGGCTGGAACCTATTCATTACAAATCAG

TAGTACTCCACTTTATAAAAAGAAAGAACTTAATCAGCTAGAAGATAAATATGACAAAGACTACCTCAGT

GGTGAACTGGGTGATAATCTGAAGATGAAAATTCAGATTTTGCTTCATTAATTCACCATACAGAGACCAA

ATAATTAAAAAAACAAAGATAGATAGGTAGAACGGATTTTTTCCCCCAATCAGAATCTTCATATCATAGG

TACAATCTTTCACCAAATAAATTTCTATAAATTAGCACTATTCTTGTATTACAAAAAGCAAGGTACAGGT

GACTACCCTAGTTCAAAACAACCACTTTCTCAGGCTTCTCATGTGTGTAGCTGAGCTACCTTGTCATATG

TGTTGATTCTTGAAAACTGGGACACGTATTTGCATTGGGGGTCAGCCCTTCACGCTGACGCACCAGCCTT

CTAGCAGATGTACAGGAAAGTGCTTTCAATCGGTGGACTGTCTTATTTTTTCAAATTTTTAAACTTTGCT

TCTCCAAACACAAGTACTAGGTTGGCCAATTATAATATCTATTTGGTGCTAGTAAATTCTCAAACTAGAT

TTATAAATGCACTGTAATATTTACACAACTTAGAAACCAAATTACAAGTATTCAGTTCAAATACTTCATT

AATTTCAATCAACCAAAGTTAGTTCAGTAGCTTATCTCAGTTATGAGTATAATACATTACATGTAAATTA

AGTGTGTGTATACTGTAATCGTGCTATTTTTTATCATTGAAACATGTATAAACTAGAATAATAATGCCCT

TAATGTGAGGGTTTGTAATGGTGCTTATTAAGACCAAAGACTTGTTAAATGTATACACCAAGTGGTAAGG

AAATTTCTGTGACTGGCCCACACGTGCATGGAGGTCTGGGAGGACCAAGAACCAGCCTCAGCAGCCAGAG

GATCACCAGTGCATCCTTCATCACACCTGTGCAATATGCCAAGATTACCCTCGGTCATTCCTATCAACAA

GGGGTCCATGTCATAAATGTCACAATAAAACAGTCTCTCTTTTTTTTAGTGTA

**Supplementary File 3B-F: mRNA Transcript alignments and protein alignments between wildtype and mutant *FBN1* sequences.** (B) Transcript alignment using EMBL-EBI Job Dispatcher Clustal Omega Multiple Sequence Alignment^39^ of predicted wildtype *FBN1* mRNA transcript (NM_001287085.2) to mutant sequence featuring exon 24 deletion. (C) Translated wildtype protein sequence using SIB Expasy Translate tool with the deleted protein sequence highlighted in red and underlined. (D) Translated mutant protein sequence using SIB Expasy Translate tool (E) Translated wildtype and mutant protein sequences aligned using EMBL-EBI Job Dispatcher Clustal Omega Multiple Sequence Alignment. (F) Translated mutant protein sequence and Fibrillin 1 precursor protein (NP_001274014.2) sequence aligned using EMBL-EBI Job Dispatcher Clustal Omega Multiple Sequence Alignment. NP_001274014.2:p.I953_1,028del

B)

```
CLUSTAL O(1.2.4) multiple sequence alignment


wildtype      TGCAGCTGGCAGGGGCCGCCTGAAGCGGGAGCAGCGCCTGGAGCAGGCGGGAGGAGCCGG	60
mutant        TGCAGCTGGCAGGGGCCGCCTGAAGCGGGAGCAGCGCCTGGAGCAGGCGGGAGGAGCCGG	60
              ************************************************************

wildtype      CCCGGGGGACGGGCGGCGGGACAGCGGGACCCCGGCGGCGCGGCGCGCTTCGGGGCGCAG	120
mutant        CCCGGGGGACGGGCGGCGGGACAGCGGGACCCCGGCGGCGCGGCGCGCTTCGGGGCGCAG	120
              ************************************************************

wildtype      CGGCGGCCGCGGATCGAGCCCGGGGCGCGGCGAGAGGCGGCAGGAGCCGGCGGCCGCTCG	180
mutant        CGGCGGCCGCGGATCGAGCCCGGGGCGCGGCGAGAGGCGGCAGGAGCCGGCGGCCGCTCG	180
              ************************************************************

wildtype      GCATCATGCGGCGGGGAGGGCTGCTGGAGGTCGTCCTGGGATTTACCGTGCTCTTAGCGT	240
mutant        GCATCATGCGGCGGGGAGGGCTGCTGGAGGTCGTCCTGGGATTTACCGTGCTCTTAGCGT	240
              ************************************************************

wildtype      CCTACACGAGCCACGGGGCGGACGCCAATTTGGAGGCTGGGAACGTGAAGGAGACCAGGG	300
mutant        CCTACACGAGCCACGGGGCGGACGCCAATTTGGAGGCTGGGAACGTGAAGGAGACCAGGG	300
              ************************************************************

wildtype      CCAGCCGGGCCAAGAGAAGAGGCGGCGGGGGACACGACGCGCTTAAAGGACCCAATGTCT	360
mutant        CCAGCCGGGCCAAGAGAAGAGGCGGCGGGGGACACGACGCGCTTAAAGGACCCAATGTCT	360
              ************************************************************

wildtype      GTGGATCACGTTATAATGCTTACTGTTGCCCTGGATGGAAAACCTTACCTGGTGGAAATC	420
mutant        GTGGATCACGTTATAATGCTTACTGTTGCCCTGGATGGAAAACCTTACCTGGTGGAAATC	420
              ************************************************************

wildtype      AATGTATTGTCCCCATTTGCCGGCATTCCTGTGGGGATGGATTTTGTTCTAGGCCAAATA	480
mutant        AATGTATTGTCCCCATTTGCCGGCATTCCTGTGGGGATGGATTTTGTTCTAGGCCAAATA	480
              ************************************************************

wildtype      TGTGCACTTGCCCATCTGGTCAGATATCTCCTTCCTGTGGCTCCAGATCCATCCAACACT	540
mutant        TGTGCACTTGCCCATCTGGTCAGATATCTCCTTCCTGTGGCTCCAGATCCATCCAACACT	540
              ************************************************************

wildtype      GTAATATTCGCTGTATGAACGGAGGTAGCTGTAGTGATGATCACTGTCTGTGCCAGAAAG	600
mutant        GTAATATTCGCTGTATGAACGGAGGTAGCTGTAGTGATGATCACTGTCTGTGCCAGAAAG	600
              ************************************************************

wildtype      GATACATAGGGACTCACTGCGGACAACCTGTCTGTGAAAGTGGCTGTCTCAATGGAGGCA	660
mutant        GATACATAGGGACTCACTGCGGACAACCTGTCTGTGAAAGTGGCTGTCTCAATGGAGGCA	660
              ************************************************************

wildtype      GGTGTGTGGCTCCGAATCGATGTGCCTGCACATACGGCTTTACTGGACCCCAGTGTGAAA	720
mutant        GGTGTGTGGCTCCGAATCGATGTGCCTGCACATACGGCTTTACTGGACCCCAGTGTGAAA	720
              ************************************************************

wildtype      GAGATTACAGGACAGGCCCATGTTTTACTGTGGTCAGCAACCAGATGTGCCAGGGACAGC	780
mutant        GAGATTACAGGACAGGCCCATGTTTTACTGTGGTCAGCAACCAGATGTGCCAGGGACAGC	780
              ************************************************************

wildtype      TCAGCGGGATTGTCTGCACAAAAACACTCTGCTGTGCCACGGTGGGCAGAGCCTGGGGCC	840
mutant        TCAGCGGGATTGTCTGCACAAAAACACTCTGCTGTGCCACGGTGGGCAGAGCCTGGGGCC	840
              ************************************************************

wildtype      ACCCGTGCGAGATGTGCCCCGCCCAGCCCCACCCCTGTCGCCGAGGCTTCATTCCAAATA	900
mutant        ACCCGTGCGAGATGTGCCCCGCCCAGCCCCACCCCTGTCGCCGAGGCTTCATTCCAAATA	900
              ************************************************************

wildtype      TCCGTACGGGAGCCTGTCAAGATGTGGATGAATGCCAGGCCATCCCTGGGCTCTGTCAAG	960
mutant        TCCGTACGGGAGCCTGTCAAGATGTGGATGAATGCCAGGCCATCCCTGGGCTCTGTCAAG	960
              ************************************************************

wildtype      GAGGAAATTGCATTAATACTGTTGGGTCTTTTGAGTGCAAATGCCCTGCTGGACACAAAT	1020
mutant        GAGGAAATTGCATTAATACTGTTGGGTCTTTTGAGTGCAAATGCCCTGCTGGACACAAAT	1020
              ************************************************************

wildtype      TTAATGAAGCATCACAAAAATGTGAAGATATTGACGAATGCAGTACCATTCCTGGAATCT	1080
mutant        TTAATGAAGCATCACAAAAATGTGAAGATATTGACGAATGCAGTACCATTCCTGGAATCT	1080
              ************************************************************

wildtype      GTGATGGGGGCGAGTGTACAAACACAGTCAGCAGTTACTTTTGCAAGTGTCCTCCTGGTT	1140
mutant        GTGATGGGGGCGAGTGTACAAACACAGTCAGCAGTTACTTTTGCAAGTGTCCTCCTGGTT	1140
              ************************************************************

wildtype      TTTACCCCTCTCCCGATGGTACCCGATGCGTAGATGTTCGCCCCGGATACTGTTACACAG	1200
mutant        TTTACCCCTCTCCCGATGGTACCCGATGCGTAGATGTTCGCCCCGGATACTGTTACACAG	1200
              ************************************************************

wildtype      CTCTGGCAAATGGGCGCTGTTCTAACCAACTGCCCCAGTCCATAACAAAAATGCAGTGCT	1260
mutant        CTCTGGCAAATGGGCGCTGTTCTAACCAACTGCCCCAGTCCATAACAAAAATGCAGTGCT	1260
              ************************************************************

wildtype      GCTGTGATGTTGGCCGATGCTGGTCTCCAGGGGTCACCGTTGCCCCTGAGATGTGTCCCA	1320
mutant        GCTGTGATGTTGGCCGATGCTGGTCTCCAGGGGTCACCGTTGCCCCTGAGATGTGTCCCA	1320
              ************************************************************

wildtype      TCAGAGCAACAGAGGATTTCAACAAGCTGTGCTCTGTCCCCTTGGTAATTCCTGAGAGAC	1380
mutant        TCAGAGCAACAGAGGATTTCAACAAGCTGTGCTCTGTCCCCTTGGTAATTCCTGAGAGAC	1380
              ************************************************************

wildtype      CAGGATATCCTCCCCCACCCCTTGGCCCCATTCCTCCAGTTCACCCTGTTTCTCCTGGCT	1440
mutant        CAGGATATCCTCCCCCACCCCTTGGCCCCATTCCTCCAGTTCACCCTGTTTCTCCTGGCT	1440
              ************************************************************

wildtype      TTCCTCCTGGACCTCCAGTTCCAGTCCCTCGACCACCAGTGGAATATCCATACCCGTCTC	1500
mutant        TTCCTCCTGGACCTCCAGTTCCAGTCCCTCGACCACCAGTGGAATATCCATACCCGTCTC	1500
              ************************************************************

wildtype      GGGAACCACCAAGGGTGCTGCCAGTCAATGTCACTGATTACTGCCAGCTGTTTCGATATC	1560
mutant        GGGAACCACCAAGGGTGCTGCCAGTCAATGTCACTGATTACTGCCAGCTGTTTCGATATC	1560
              ************************************************************

wildtype      TCTGTCAAAATGGACGCTGCATTCCAACTCCTGGGAGCTATCGGTGTGAATGCAACAAAG	1620
mutant        TCTGTCAAAATGGACGCTGCATTCCAACTCCTGGGAGCTATCGGTGTGAATGCAACAAAG	1620
              ************************************************************

wildtype      GATTCCAGCTGGACCTTCGTGGGGAGTGCATTGATGTTGATGAATGTGAGAAGAACCCTT	1680
mutant        GATTCCAGCTGGACCTTCGTGGGGAGTGCATTGATGTTGATGAATGTGAGAAGAACCCTT	1680
              ************************************************************

wildtype      GTGCTGGTGGAGAGTGTATTAATAACCAGGGCTCGTACACCTGTCAGTGCCGACCTGGCT	1740
mutant        GTGCTGGTGGAGAGTGTATTAATAACCAGGGCTCGTACACCTGTCAGTGCCGACCTGGCT	1740
              ************************************************************

wildtype      ATCAAAGCACACTCACCCGGACAGAGTGCCGAGACATCGACGAATGTTTACAGAATGGCC	1800
mutant        ATCAAAGCACACTCACCCGGACAGAGTGCCGAGACATCGACGAATGTTTACAGAATGGCC	1800
              ************************************************************

wildtype      GTATCTGCAATAATGGGCGTTGCATTAACACAGATGGCAGTTTCCACTGTGTGTGTAATG	1860
mutant        GTATCTGCAATAATGGGCGTTGCATTAACACAGATGGCAGTTTCCACTGTGTGTGTAATG	1860
              ************************************************************

wildtype      CAGGCTTTCATGTGACACGAGATGGGAAGAACTGTGAAGATATGGATGAATGCAGCATAA	1920
mutant        CAGGCTTTCATGTGACACGAGATGGGAAGAACTGTGAAGATATGGATGAATGCAGCATAA	1920
              ************************************************************

wildtype      GGAACATGTGCCTCAATGGAATGTGTATCAATGAAGATGGCAGTTTTAAATGTATTTGCA	1980
mutant        GGAACATGTGCCTCAATGGAATGTGTATCAATGAAGATGGCAGTTTTAAATGTATTTGCA	1980
              ************************************************************

wildtype      AACCTGGATTTCAGCTGGCATCAGACGGGCGTTATTGCAAAGATATTAATGAGTGTGAAA	2040
mutant        AACCTGGATTTCAGCTGGCATCAGACGGGCGTTATTGCAAAGATATTAATGAGTGTGAAA	2040
              ************************************************************

wildtype      CACCTGGGATCTGCATGAATGGACGCTGTGTCAACACTGATGGCTCCTACAGATGTGAAT	2100
mutant        CACCTGGGATCTGCATGAATGGACGCTGTGTCAACACTGATGGCTCCTACAGATGTGAAT	2100
              ************************************************************

wildtype      GCTTTCCTGGACTGGCTGTGGGTCTCGATGGTCGTGTGTGTGTTGACACACACATGCGGA	2160
mutant        GCTTTCCTGGACTGGCTGTGGGTCTCGATGGTCGTGTGTGTGTTGACACACACATGCGGA	2160
              ************************************************************

wildtype      GCACATGCTATGGTGGATATAAGAGAGGCCAGTGTGTCAAACCGTTGTTTGGTGCCGTTA	2220
mutant        GCACATGCTATGGTGGATATAAGAGAGGCCAGTGTGTCAAACCGTTGTTTGGTGCCGTTA	2220
              ************************************************************

wildtype      CTAAATCTGAATGCTGTTGTGCCAGCACTGAGTATGCTTTTGGGGAACCTTGCCAACCAT	2280
mutant        CTAAATCTGAATGCTGTTGTGCCAGCACTGAGTATGCTTTTGGGGAACCTTGCCAACCAT	2280
              ************************************************************

wildtype      GTCCTGCACAAAATTCAGCGGAATATCAGGCACTCTGCAGCAGTGGGCCAGGAATGACAT	2340
mutant        GTCCTGCACAAAATTCAGCGGAATATCAGGCACTCTGCAGCAGTGGGCCAGGAATGACAT	2340
              ************************************************************

wildtype      CAGCAGGCAGTGATATAAATGAATGTGCGTTAGATCCGGATATCTGTCCAAACGGAATTT	2400
mutant        CAGCAGGCAGTGATATAAATGAATGTGCGTTAGATCCGGATATCTGTCCAAACGGAATTT	2400
              ************************************************************

wildtype      GTGAAAATCTCCGTGGGACCTACAAATGCATATGCAATTCAGGATATGAAGTGGATCCAT	2460
mutant        GTGAAAATCTCCGTGGGACCTACAAATGCATATGCAATTCAGGATATGAAGTGGATCCAT	2460
              ************************************************************

wildtype      CTGGGAAGAACTGTGTTGATATTAATGAATGTGTATTGAACAGTCTGCTTTGTGATAATG	2520
mutant        CTGGGAAGAACTGTGTTGATATTAATGAATGTGTATTGAACAGTCTGCTTTGTGATAATG	2520
              ************************************************************

wildtype      GACAATGTAGAAACACTCCTGGAAGTTTTGTCTGTACCTGTCCCAAGGGATTTATCTACA	2580
mutant        GACAATGTAGAAACACTCCTGGAAGTTTTGTCTGTACCTGTCCCAAGGGATTTATCTACA	2580
              ************************************************************

wildtype      AACCTGACCTAAAAACCTGTGAAGACATCGACGAATGTGAATCAAGTCCTTGCATTAATG	2640
mutant        AACCTGACCTAAAAACCTGTGAAGACATCGACGAATGTGAATCAAGTCCTTGCATTAATG	2640
              ************************************************************

wildtype      GAGTCTGCAAGAACAGCCCGGGCTCTTTTATTTGTGAATGTTCTTCCGAAAGTACCCTGG	2700
mutant        GAGTCTGCAAGAACAGCCCGGGCTCTTTTATTTGTGAATGTTCTTCCGAAAGTACCCTGG	2700
              ************************************************************

wildtype      ATCCAACAAAAACCATCTGCATAGAAACCATCAAGGGCACTTGCTGGCAGACTGTCATCG	2760
mutant        ATCCAACAAAAACCATCTGCATAGAAACCATCAAGGGCACTTGCTGGCAGACTGTCATCG	2760
              ************************************************************

wildtype      ATGGGCGGTGTGAGATCAACATCAATGGAGCTACCTTAAAGTCCCAGTGCTGTTCCTCTC	2820
mutant        ATGGGCGGTGTGAGATCAACATCAATGGAGCTACCTTAAAGTCCCAGTGCTGTTCCTCTC	2820
              ************************************************************

wildtype      TTGGTGCTGCCTGGGGGAGCCCTTGTACCCTATGCCAAGTCGATCCCATATGTGGCAAGG	2880
mutant        TTGGTGCTGCCTGGGGGAGCCCTTGTACCCTATGCCAAGTCGATCCCATATGTGGCAAGG	2880
              ************************************************************

wildtype      GCTACTCAAGAATTAAAGGAACACAGTGTGAAGATATAAATGAGTGTGAAGTATTTCCAG	2940
mutant        GCTACTCAAGAATTAAAGGAACACAGTGTGAAGATATAAATGAGTGTGAAGTATTTCCAG	2940
              ************************************************************

wildtype      GAGTATGCAAAAATGGTGTATGTGTTAACTCTGAGGGATCATTTAAATGTCAGTGTCCCA	3000
mutant        GAGTATGCAAAAATGGTGTATGTGTTAACTCTGAGGGATCATTTAAATGTCAGTGTCCCA	3000
              ************************************************************

wildtype      ATGGGATGACTTTGGATGCCACAGGAAGGATCTGCCTAGATATCCGCCTGGAAACCTGCT	3060
mutant        ATGGGATGACTTTGGATGCCACAGGAAGGATCTGCCTAGAT-------------------	3041
              *****************************************

wildtype      TCCTGAGGTATGAGGATGAGGAATGCACTCTGCCCGTTGCTGGCCGCCACCGCATGGACG	3120
mutant        ------------------------------------------------------------	3041


wildtype      CCTGTTGCTGCTCCGTTGGGGCAGCCTGGGGTACTGAGGAATGCGAGGAGTGTCCTGTGA	3180
mutant        ------------------------------------------------------------	3041


wildtype      GAAACACGCCTGAGTACGAGGAGCTCTGTCCCAGAGGGCCCGGATTCGCCACAAAAGAAA	3240
mutant        ------------------------------------------------------------	3041


wildtype      TTACCAATGGAAAACCTTTCTTCAAAGATATCAACGAATGCAAGATGATTCCCAGCCTCT	3300
mutant        -----------------------------ATCAACGAATGCAAGATGATTCCCAGCCTCT	3072
                                           *******************************

wildtype      GTACCCACGGCAAATGCAGAAACACCATTGGCAGCTTTAAGTGCAGGTGTGACAGTGGCT	3360
mutant        GTACCCACGGCAAATGCAGAAACACCATTGGCAGCTTTAAGTGCAGGTGTGACAGTGGCT	3132
              ************************************************************

wildtype      TTGCTCTTGACTCTGAAGAAAGGAATTGCACAGACATCGATGAATGCCGAATCTCCCCTG	3420
mutant        TTGCTCTTGACTCTGAAGAAAGGAATTGCACAGACATCGATGAATGCCGAATCTCCCCTG	3192
              ************************************************************

wildtype      ACCTCTGCGGCCGAGGCCAGTGCGTGAACACCCCAGGGGACTTCGAATGCAAGTGTGACG	3480
mutant        ACCTCTGCGGCCGAGGCCAGTGCGTGAACACCCCAGGGGACTTCGAATGCAAGTGTGACG	3252
              ************************************************************

wildtype      AAGGCTATGAAAGCGGATTCATGATGATGAAGAACTGCATGGATATCGATGAGTGTCAGA	3540
mutant        AAGGCTATGAAAGCGGATTCATGATGATGAAGAACTGCATGGATATCGATGAGTGTCAGA	3312
              ************************************************************

wildtype      GAGATCCTCTCCTGTGCCGGGGTGGCGTTTGCCTCAACACTGAGGGAAGTTACCGCTGTG	3600
mutant        GAGATCCTCTCCTGTGCCGGGGTGGCGTTTGCCTCAACACTGAGGGAAGTTACCGCTGTG	3372
              ************************************************************

wildtype      AGTGTCCTCCTGGTCATCAGCTCTCTCCCAACATCTCAGCATGTATAGACATCAACGAGT	3660
mutant        AGTGTCCTCCTGGTCATCAGCTCTCTCCCAACATCTCAGCATGTATAGACATCAACGAGT	3432
              ************************************************************

wildtype      GTGAACTGAGTGCGAACCTCTGCCCTAATGGCCGTTGTGTGAACCTCATCGGGAAGTATC	3720
mutant        GTGAACTGAGTGCGAACCTCTGCCCTAATGGCCGTTGTGTGAACCTCATCGGGAAGTATC	3492
              ************************************************************

wildtype      AGTGTGCTTGCAACCCTGGCTATCATTCAACTCCTGACAGGCTGTTTTGTGTGGACATCG	3780
mutant        AGTGTGCTTGCAACCCTGGCTATCATTCAACTCCTGACAGGCTGTTTTGTGTGGACATCG	3552
              ************************************************************

wildtype      ACGAATGCAGCATAATGAATGGTGGTTGTGAGACCTTCTGTACAAACTCTGAAGGCAGCT	3840
mutant        ACGAATGCAGCATAATGAATGGTGGTTGTGAGACCTTCTGTACAAACTCTGAAGGCAGCT	3612
              ************************************************************

wildtype      ATGAATGTAGCTGTCAGCCAGGATTTGCACTAATGCCCGACCAGAGGTCATGCACTGACA	3900
mutant        ATGAATGTAGCTGTCAGCCAGGATTTGCACTAATGCCCGACCAGAGGTCATGCACTGACA	3672
              ************************************************************

wildtype      TTGATGAGTGTGAAGATAACCCCAATATCTGTGACGGCGGTCAGTGTACAAACATTCCCG	3960
mutant        TTGATGAGTGTGAAGATAACCCCAATATCTGTGACGGCGGTCAGTGTACAAACATTCCCG	3732
              ************************************************************

wildtype      GGGAGTACAGGTGCTTGTGTTATGATGGATTCATGGCATCTGAAGACATGAAGACTTGTG	4020
mutant        GGGAGTACAGGTGCTTGTGTTATGATGGATTCATGGCATCTGAAGACATGAAGACTTGTG	3792
              ************************************************************

wildtype      TAGATGTCAATGAATGTGACCTAAATCCAAATATCTGCCTAAGTGGAACCTGTGAAAACA	4080
mutant        TAGATGTCAATGAATGTGACCTAAATCCAAATATCTGCCTAAGTGGAACCTGTGAAAACA	3852
              ************************************************************

wildtype      CAAAAGGCTCGTTTATTTGCCACTGTGATATGGGATATTCAGGCAAAAAAGGAAAAACTG	4140
mutant        CAAAAGGCTCGTTTATTTGCCACTGTGATATGGGATATTCAGGCAAAAAAGGAAAAACTG	3912
              ************************************************************

wildtype      GCTGTACAGACATCAACGAATGTGAAATTGGAGCACACAACTGTGACAGACACGCCGTAT	4200
mutant        GCTGTACAGACATCAACGAATGTGAAATTGGAGCACACAACTGTGACAGACACGCCGTAT	3972
              ************************************************************

wildtype      GTACCAACACAGCAGGAAGCTTCAAATGTAGCTGCAGCCCTGGATGGATTGGAGATGGCA	4260
mutant        GTACCAACACAGCAGGAAGCTTCAAATGTAGCTGCAGCCCTGGATGGATTGGAGATGGCA	4032
              ************************************************************

wildtype      TTAAGTGCACGGATCTGGATGAATGCTCCAATGGAACACACATGTGCAGCCAACATGCAG	4320
mutant        TTAAGTGCACGGATCTGGATGAATGCTCCAATGGAACACACATGTGCAGCCAACATGCAG	4092
              ************************************************************

wildtype      ACTGCAAGAATACCATGGGCTCTTATCGCTGCCTCTGTAAAGAAGGATACACGGGTGATG	4380
mutant        ACTGCAAGAATACCATGGGCTCTTATCGCTGCCTCTGTAAAGAAGGATACACGGGTGATG	4152
              ************************************************************

wildtype      GATTCACGTGTACAGATCTTGATGAGTGCTCTGAGAACCTGAATCTTTGTGGCAATGGTC	4440
mutant        GATTCACGTGTACAGATCTTGATGAGTGCTCTGAGAACCTGAATCTTTGTGGCAATGGTC	4212
              ************************************************************

wildtype      AGTGCCTGAATGCCCCAGGAGGATACCGCTGCGAATGTGACATGGGCTTTGTGCCCAGTG	4500
mutant        AGTGCCTGAATGCCCCAGGAGGATACCGCTGCGAATGTGACATGGGCTTTGTGCCCAGTG	4272
              ************************************************************

wildtype      CTGATGGGAAAGCCTGTGAAGATATCGATGAGTGCTCCCTACCGAACATCTGTGTTTTTG	4560
mutant        CTGATGGGAAAGCCTGTGAAGATATCGATGAGTGCTCCCTACCGAACATCTGTGTTTTTG	4332
              ************************************************************

wildtype      GAACTTGCCACAACCTTCCTGGCCTGTTCCGTTGTGAGTGCGAGATAGGCTACGAATTGG	4620
mutant        GAACTTGCCACAACCTTCCTGGCCTGTTCCGTTGTGAGTGCGAGATAGGCTACGAATTGG	4392
              ************************************************************

wildtype      ACAGAAGTGGCGGAAACTGCACAGATGTTAATGAGTGTCTGGATCCAACCACATGCATCA	4680
mutant        ACAGAAGTGGCGGAAACTGCACAGATGTTAATGAGTGTCTGGATCCAACCACATGCATCA	4452
              ************************************************************

wildtype      GTGGGAACTGTGTCAACACTCCAGGCAGCTATACCTGTGATTGTCCTCCCGACTTTGAGT	4740
mutant        GTGGGAACTGTGTCAACACTCCAGGCAGCTATACCTGTGATTGTCCTCCCGACTTTGAGT	4512
              ************************************************************

wildtype      TGAACCCAACTCGAGTTGGCTGTGTCGATACCCGTTCTGGAAATTGCTATCTGGATATTC	4800
mutant        TGAACCCAACTCGAGTTGGCTGTGTCGATACCCGTTCTGGAAATTGCTATCTGGATATTC	4572
              ************************************************************

wildtype      GACCTCGAGGAGACAATGGAGATACAGCCTGTAGCAATGAAATTGGAGTTGGTGTCTCCA	4860
mutant        GACCTCGAGGAGACAATGGAGATACAGCCTGTAGCAATGAAATTGGAGTTGGTGTCTCCA	4632
              ************************************************************

wildtype      AGGCTTCCTGCTGTTGTTCTTTGGGTAAAGCCTGGGGTACTCCTTGTGAGCTGTGCCCTC	4920
mutant        AGGCTTCCTGCTGTTGTTCTTTGGGTAAAGCCTGGGGTACTCCTTGTGAGCTGTGCCCTC	4692
              ************************************************************

wildtype      CTGTGAACACATCTGAGTATAAAATTCTCTGTCCTGGAGGAGAAGGTTTTCGGCCCAATC	4980
mutant        CTGTGAACACATCTGAGTATAAAATTCTCTGTCCTGGAGGAGAAGGTTTTCGGCCCAATC	4752
              ************************************************************

wildtype      CTATCACTGTCATATTGGAAGACATTGACGAGTGCCAGGAGCTCCCAGGGCTGTGCCAAG	5040
mutant        CTATCACTGTCATATTGGAAGACATTGACGAGTGCCAGGAGCTCCCAGGGCTGTGCCAAG	4812
              ************************************************************

wildtype      GCGGGAAATGTATCAACACGTTTGGCAGTTTCCAATGCCGCTGTCCAACTGGTTACTACC	5100
mutant        GCGGGAAATGTATCAACACGTTTGGCAGTTTCCAATGCCGCTGTCCAACTGGTTACTACC	4872
              ************************************************************

wildtype      TGAATGAAGACACCCGAGTGTGTGATGATGTGAATGAATGTGAAACTCCCGGAATCTGTG	5160
mutant        TGAATGAAGACACCCGAGTGTGTGATGATGTGAATGAATGTGAAACTCCCGGAATCTGTG	4932
              ************************************************************

wildtype      GTCCAGGAACATGTTACAACACTGTCGGCAACTACACCTGTATCTGCCCTCCAGACTACA	5220
mutant        GTCCAGGAACATGTTACAACACTGTCGGCAACTACACCTGTATCTGCCCTCCAGACTACA	4992
              ************************************************************

wildtype      TGCAAGTGAATGGGGGGAATAATTGCATGGATATGAGAAGAAGTTTGTGCTATAGAAACT	5280
mutant        TGCAAGTGAATGGGGGGAATAATTGCATGGATATGAGAAGAAGTTTGTGCTATAGAAACT	5052
              ************************************************************

wildtype      ACTATGCTGACAACCAAACCTGTGATGGAGAACTTTTGTTCAATATGACCAAGAAGATGT	5340
mutant        ACTATGCTGACAACCAAACCTGTGATGGAGAACTTTTGTTCAATATGACCAAGAAGATGT	5112
              ************************************************************

wildtype      GCTGTTGTTCCTACAACATTGGCCGGGCCTGGAATAAGCCCTGTGAACAGTGTCCTATCC	5400
mutant        GCTGTTGTTCCTACAACATTGGCCGGGCCTGGAATAAGCCCTGTGAACAGTGTCCTATCC	5172
              ************************************************************

wildtype      CAAGCACAGATGAATTTGCTACACTTTGTGGAAGTCAAAGGCCAGGCTTCGTCATTGACA	5460
mutant        CAAGCACAGATGAATTTGCTACACTTTGTGGAAGTCAAAGGCCAGGCTTCGTCATTGACA	5232
              ************************************************************

wildtype      TTTACACCGGGTTACCTGTTGATATTGATGAATGCCGGGAGATCCCTGGGGTCTGTGAGA	5520
mutant        TTTACACCGGGTTACCTGTTGATATTGATGAATGCCGGGAGATCCCTGGGGTCTGTGAGA	5292
              ************************************************************

wildtype      ATGGAGTGTGTATCAACATGGTTGGCAGCTTCAGATGTGAATGTCCGGTGGGCTTCTTCT	5580
mutant        ATGGAGTGTGTATCAACATGGTTGGCAGCTTCAGATGTGAATGTCCGGTGGGCTTCTTCT	5352
              ************************************************************

wildtype      ATAATGACAAGTTGTTGGTTTGTGAAGATATTGACGAGTGTCAGAACGGTCCTGTGTGCC	5640
mutant        ATAATGACAAGTTGTTGGTTTGTGAAGATATTGACGAGTGTCAGAACGGTCCTGTGTGCC	5412
              ************************************************************

wildtype      AGCGCAATGCCGAGTGCATCAACACAGCAGGCAGTTTCCGATGTGACTGCAAGCCCGGCT	5700
mutant        AGCGCAATGCCGAGTGCATCAACACAGCAGGCAGTTTCCGATGTGACTGCAAGCCCGGCT	5472
              ************************************************************

wildtype      ACCGCTTCACTTCCACAGGCCAGTGCAACGATCGCAATGAATGTCAAGAAATCCCCAATA	5760
mutant        ACCGCTTCACTTCCACAGGCCAGTGCAACGATCGCAATGAATGTCAAGAAATCCCCAATA	5532
              ************************************************************

wildtype      TTTGCAGCCATGGACAGTGTATCGACACAGTTGGAAGTTTTTATTGCCTTTGCCACACTG	5820
mutant        TTTGCAGCCATGGACAGTGTATCGACACAGTTGGAAGTTTTTATTGCCTTTGCCACACTG	5592
              ************************************************************

wildtype      GTTTTAAAACAAATGCTGATCAAACCATGTGTTTGGACATAAATGAGTGTGAGCGAGATG	5880
mutant        GTTTTAAAACAAATGCTGATCAAACCATGTGTTTGGACATAAATGAGTGTGAGCGAGATG	5652
              ************************************************************

wildtype      CCTGTGGGAATGGAACTTGCCGCAACACGATTGGGTCCTTCAACTGCCGCTGCAACCACG	5940
mutant        CCTGTGGGAATGGAACTTGCCGCAACACGATTGGGTCCTTCAACTGCCGCTGCAACCACG	5712
              ************************************************************

wildtype      GCTTCATCCTTTCCCACAACAATGACTGCATAGATGTTGATGAATGTGCAACTGGAAATG	6000
mutant        GCTTCATCCTTTCCCACAACAATGACTGCATAGATGTTGATGAATGTGCAACTGGAAATG	5772
              ************************************************************

wildtype      GGAACCTTTGCAGAAATGGCCAATGCATCAATACAGTGGGGTCCTTCCAGTGTCAGTGCA	6060
mutant        GGAACCTTTGCAGAAATGGCCAATGCATCAATACAGTGGGGTCCTTCCAGTGTCAGTGCA	5832
              ************************************************************

wildtype      ATGAAGGCTATGAGGTGGCTCCAGATGGGAGGACCTGTGTGGATATCAATGAATGTCTTC	6120
mutant        ATGAAGGCTATGAGGTGGCTCCAGATGGGAGGACCTGTGTGGATATCAATGAATGTCTTC	5892
              ************************************************************

wildtype      TAGAACCTGGAAAATGTGCACCAGGCACCTGTCAAAACTTGGATGGGTCCTACAGGTGCA	6180
mutant        TAGAACCTGGAAAATGTGCACCAGGCACCTGTCAAAACTTGGATGGGTCCTACAGGTGCA	5952
              ************************************************************

wildtype      TTTGTCCACCTGGATACAGTCTACAGAATGACAAGTGTGAAGATATTGATGAGTGTGTTG	6240
mutant        TTTGTCCACCTGGATACAGTCTACAGAATGACAAGTGTGAAGATATTGATGAGTGTGTTG	6012
              ************************************************************

wildtype      AAGAGCCAGAAATCTGTGCCTTGGGAACATGCAGCAACACAGAAGGCAGCTTTAAATGTC	6300
mutant        AAGAGCCAGAAATCTGTGCCTTGGGAACATGCAGCAACACAGAAGGCAGCTTTAAATGTC	6072
              ************************************************************

wildtype      TCTGTCCAGATGGATTCTCCTTGTCCTCCACTGGAAGAAGGTGCCAAGATCTGCGAATGA	6360
mutant        TCTGTCCAGATGGATTCTCCTTGTCCTCCACTGGAAGAAGGTGCCAAGATCTGCGAATGA	6132
              ************************************************************

wildtype      GCTACTGCTATGCTAAGTTTGAAGGAGGAAAATGTTCATCACCCAAATCCAGAAATCACT	6420
mutant        GCTACTGCTATGCTAAGTTTGAAGGAGGAAAATGTTCATCACCCAAATCCAGAAATCACT	6192
              ************************************************************

wildtype      CCAAGCAGGAATGCTGTTGTGCCTTGAAGGGAGAAGGTTGGGGTGATCCCTGTGAGCTGT	6480
mutant        CCAAGCAGGAATGCTGTTGTGCCTTGAAGGGAGAAGGTTGGGGTGATCCCTGTGAGCTGT	6252
              ************************************************************

wildtype      GCCCCACCGAACCTGATGAGGCTTTCCGCCAGATCTGTCCCTATGGAAGTGGCATCATTG	6540
mutant        GCCCCACCGAACCTGATGAGGCTTTCCGCCAGATCTGTCCCTATGGAAGTGGCATCATTG	6312
              ************************************************************

wildtype      TGGGGCCTGATGATTCAGCAGTTGATATGGATGAGTGCAAAGAACCTGATGTCTGTAAAC	6600
mutant        TGGGGCCTGATGATTCAGCAGTTGATATGGATGAGTGCAAAGAACCTGATGTCTGTAAAC	6372
              ************************************************************

wildtype      ATGGGCAGTGCATCAATACAGATGGCTCATACCGCTGCGAGTGTCCCTTTGGTTACATTC	6660
mutant        ATGGGCAGTGCATCAATACAGATGGCTCATACCGCTGCGAGTGTCCCTTTGGTTACATTC	6432
              ************************************************************

wildtype      TGGAAGGGAATGAATGTGTAGATACTGATGAATGTTCTGTGGGCAATCCTTGTGGAAATG	6720
mutant        TGGAAGGGAATGAATGTGTAGATACTGATGAATGTTCTGTGGGCAATCCTTGTGGAAATG	6492
              ************************************************************

wildtype      GAACCTGCAAGAATGTGATTGGAGGTTTTGAATGTACCTGTGAGGAGGGATTTGAGCCTG	6780
mutant        GAACCTGCAAGAATGTGATTGGAGGTTTTGAATGTACCTGTGAGGAGGGATTTGAGCCTG	6552
              ************************************************************

wildtype      GTCCAATGATGACTTGTGAAGATATAAACGAATGTGCTCAGAATCCTCTGCTCTGTGCTT	6840
mutant        GTCCAATGATGACTTGTGAAGATATAAACGAATGTGCTCAGAATCCTCTGCTCTGTGCTT	6612
              ************************************************************

wildtype      TCCGGTGTGTAAACACTTATGGGTCATATGAATGCAAATGTCCCGCTGGATATGTTCTGA	6900
mutant        TCCGGTGTGTAAACACTTATGGGTCATATGAATGCAAATGTCCCGCTGGATATGTTCTGA	6672
              ************************************************************

wildtype      GAGAAGATAGAAGGATGTGCAAAGATGAGGATGAGTGTGAAGAGGGCAAACACGACTGTG	6960
mutant        GAGAAGATAGAAGGATGTGCAAAGATGAGGATGAGTGTGAAGAGGGCAAACACGACTGTG	6732
              ************************************************************

wildtype      CTGAGAAGCAGATGGAGTGCAAGAACCTCATTGGCACATACATCTGCATCTGTGGGCCTG	7020
mutant        CTGAGAAGCAGATGGAGTGCAAGAACCTCATTGGCACATACATCTGCATCTGTGGGCCTG	6792
              ************************************************************

wildtype      GCTATCAGCGGAGACCTGATGGAGAGGGCTGCGTAGATGAGAATGAATGTCAGACTAAGC	7080
mutant        GCTATCAGCGGAGACCTGATGGAGAGGGCTGCGTAGATGAGAATGAATGTCAGACTAAGC	6852
              ************************************************************

wildtype      CGGGGATCTGTGAGAATGGGCGCTGCCTCAACACGCGGGGGAGTTACACTTGTGAATGCA	7140
mutant        CGGGGATCTGTGAGAATGGGCGCTGCCTCAACACGCGGGGGAGTTACACTTGTGAATGCA	6912
              ************************************************************

wildtype      GTGACGGCTTCACCCCCAGCCCTGCCCAGGATGAGTGCCTTGACAACCGGGAAGGGTACT	7200
mutant        GTGACGGCTTCACCCCCAGCCCTGCCCAGGATGAGTGCCTTGACAACCGGGAAGGGTACT	6972
              ************************************************************

wildtype      GCTTCACGGAGGTGCTCCAAAACATGTGTCAGATCGGCTCAAGCAACAGGAACCCTGTCA	7260
mutant        GCTTCACGGAGGTGCTCCAAAACATGTGTCAGATCGGCTCAAGCAACAGGAACCCTGTCA	7032
              ************************************************************

wildtype      CCAAGTCTGAATGCTGCTGTGATGGAGGGAGAGGCTGGGGCCCCCACTGTGAGATCTGTC	7320
mutant        CCAAGTCTGAATGCTGCTGTGATGGAGGGAGAGGCTGGGGCCCCCACTGTGAGATCTGTC	7092
              ************************************************************

wildtype      CTTTCCAGGGCACTGTGGCCTTCAAAAAACTCTGCCCCCATGGCCGCGGATTTATGACCA	7380
mutant        CTTTCCAGGGCACTGTGGCCTTCAAAAAACTCTGCCCCCATGGCCGCGGATTTATGACCA	7152
              ************************************************************

wildtype      ACGGAGCAGATATTGATGAATGCAAGGTTATTCATGATGTTTGTCGAAATGGGGAGTGTG	7440
mutant        ACGGAGCAGATATTGATGAATGCAAGGTTATTCATGATGTTTGTCGAAATGGGGAGTGTG	7212
              ************************************************************

wildtype      TCAACGACAGAGGATCCTATCATTGCCTATGTAAAAATGGCTACACTCCAGATATAACTG	7500
mutant        TCAACGACAGAGGATCCTATCATTGCCTATGTAAAAATGGCTACACTCCAGATATAACTG	7272
              ************************************************************

wildtype      GAACTTCCTGTGTAGATCTGAATGAGTGCAACCAGGCCCCCAAACCCTGCAATTTTATCT	7560
mutant        GAACTTCCTGTGTAGATCTGAATGAGTGCAACCAGGCCCCCAAACCCTGCAATTTTATCT	7332
              ************************************************************

wildtype      GCAAAAACACAGGAGGGAGTTTCCAGTGTTCCTGCCCGAAAGGCTACATCCTGCAGGAGG	7620
mutant        GCAAAAACACAGGAGGGAGTTTCCAGTGTTCCTGCCCGAAAGGCTACATCCTGCAGGAGG	7392
              ************************************************************

wildtype      ATGGAAGGAGCTGCAAAGATCTTGATGAATGTGCCACTAAACAGCACAACTGCCAGTTCC	7680
mutant        ATGGAAGGAGCTGCAAAGATCTTGATGAATGTGCCACTAAACAGCACAACTGCCAGTTCC	7452
              ************************************************************

wildtype      TGTGTGTTAACACCATTGGCAGCTTCACTTGTAAATGTCCTCCCGGATTTACACAACACC	7740
mutant        TGTGTGTTAACACCATTGGCAGCTTCACTTGTAAATGTCCTCCCGGATTTACACAACACC	7512
              ************************************************************

wildtype      ATACTGCCTGCATTGATAACAATGAATGCACTTCTGACATCAACTTGTGTGGGTCTAAGG	7800
mutant        ATACTGCCTGCATTGATAACAATGAATGCACTTCTGACATCAACTTGTGTGGGTCTAAGG	7572
              ************************************************************

wildtype      GTATTTGCCAGAATACTCCAGGGAGCTTCACCTGTGAATGCCAGCGGGGATTCTCACTTG	7860
mutant        GTATTTGCCAGAATACTCCAGGGAGCTTCACCTGTGAATGCCAGCGGGGATTCTCACTTG	7632
              ************************************************************

wildtype      ATCAGAGTGGTGCCAGCTGTGAAGATGTGGATGAGTGTGAAGGAAACCATCGCTGCCAGC	7920
mutant        ATCAGAGTGGTGCCAGCTGTGAAGATGTGGATGAGTGTGAAGGAAACCATCGCTGCCAGC	7692
              ************************************************************

wildtype      ACGGCTGCCAGAACATCATCGGAGGCTACAGGTGCAGCTGCCCCCAGGGCTATCTCCAGC	7980
mutant        ACGGCTGCCAGAACATCATCGGAGGCTACAGGTGCAGCTGCCCCCAGGGCTATCTCCAGC	7752
              ************************************************************

wildtype      ACTACCAATGGAACCAGTGTGTTGATGAAAACGAATGCCTCAGCGCACACATTTGTGGAG	8040
mutant        ACTACCAATGGAACCAGTGTGTTGATGAAAACGAATGCCTCAGCGCACACATTTGTGGAG	7812
              ************************************************************

wildtype      GAGCCTCCTGTCACAACACCCTGGGAAGCTACAAGTGCATGTGCCCCGCCGGCTTCCAGT	8100
mutant        GAGCCTCCTGTCACAACACCCTGGGAAGCTACAAGTGCATGTGCCCCGCCGGCTTCCAGT	7872
              ************************************************************

wildtype      ACGAACAGTTCAGTGGAGGGTGCCAAGACATCAACGAATGTGGCTCTGCACAAGCCCCCT	8160
mutant        ACGAACAGTTCAGTGGAGGGTGCCAAGACATCAACGAATGTGGCTCTGCACAAGCCCCCT	7932
              ************************************************************

wildtype      GCAGCTATGGATGCTCCAATACTGAGGGTGGCTACCTGTGTGGCTGTCCACCTGGTTACT	8220
mutant        GCAGCTATGGATGCTCCAATACTGAGGGTGGCTACCTGTGTGGCTGTCCACCTGGTTACT	7992
              ************************************************************

wildtype      TCCGAATAGGCCAAGGGCACTGTGTTTCTGGAATTGGCATGGGCCGAGGAGGCTCGGAGC	8280
mutant        TCCGAATAGGCCAAGGGCACTGTGTTTCTGGAATTGGCATGGGCCGAGGAGGCTCGGAGC	8052
              ************************************************************

wildtype      CACCTGCCAGTGGTGAAATGGATGACAATTCTCTCTCCCCGGAGGCTTGCTATGAGTGTA	8340
mutant        CACCTGCCAGTGGTGAAATGGATGACAATTCTCTCTCCCCGGAGGCTTGCTATGAGTGTA	8112
              ************************************************************

wildtype      AGATCAATGGCTACCCCAAACGAGGCAGGAAACGGAGAAGTGCAAATGAAACAGATGCCT	8400
mutant        AGATCAATGGCTACCCCAAACGAGGCAGGAAACGGAGAAGTGCAAATGAAACAGATGCCT	8172
              ************************************************************

wildtype      CCAACATTGAGGACCAGCCTGAGAGAGATACCAATGTGAGTCTTGCAAGTTGGGACACTG	8460
mutant        CCAACATTGAGGACCAGCCTGAGAGAGATACCAATGTGAGTCTTGCAAGTTGGGACACTG	8232
              ************************************************************

wildtype      AGAAGACAGCCATGTTCGCGTTCAATATTTCCCACATCAGTAACAAGGTCCGAATCCTAG	8520
mutant        AGAAGACAGCCATGTTCGCGTTCAATATTTCCCACATCAGTAACAAGGTCCGAATCCTAG	8292
              ************************************************************

wildtype      AGCTCCTCCCGGCCCTGACGACTCTGACAAATCACAACAAATACTTGATCGAATCTGGAA	8580
mutant        AGCTCCTCCCGGCCCTGACGACTCTGACAAATCACAACAAATACTTGATCGAATCTGGAA	8352
              ************************************************************

wildtype      ATGAAAATGGCTTCTTTAAAATCAACCAAAAGGAAGGGATCAGCTACCTCCACTTCACCA	8640
mutant        ATGAAAATGGCTTCTTTAAAATCAACCAAAAGGAAGGGATCAGCTACCTCCACTTCACCA	8412
              ************************************************************

wildtype      AGAAGAAGCCAGTGGCTGGAACCTATTCATTACAAATCAGTAGTACTCCACTTTATAAAA	8700
mutant        AGAAGAAGCCAGTGGCTGGAACCTATTCATTACAAATCAGTAGTACTCCACTTTATAAAA	8472
              ************************************************************

wildtype      AGAAAGAACTTAATCAGCTAGAAGATAAATATGACAAAGACTACCTCAGTGGTGAACTGG	8760
mutant        AGAAAGAACTTAATCAGCTAGAAGATAAATATGACAAAGACTACCTCAGTGGTGAACTGG	8532
              ************************************************************

wildtype      GTGATAATCTGAAGATGAAAATTCAGATTTTGCTTCATTAATTCACCATACAGAGACCAA	8820
mutant        GTGATAATCTGAAGATGAAAATTCAGATTTTGCTTCATTAATTCACCATACAGAGACCAA	8592
              ************************************************************

wildtype      ATAATTAAAAAAACAAAGATAGATAGGTAGAACGGATTTTTTCCCCCAATCAGAATCTTC	8880
mutant        ATAATTAAAAAAACAAAGATAGATAGGTAGAACGGATTTTTTCCCCCAATCAGAATCTTC	8652
              ************************************************************

wildtype      ATATCATAGGTACAATCTTTCACCAAATAAATTTCTATAAATTAGCACTATTCTTGTATT	8940
mutant        ATATCATAGGTACAATCTTTCACCAAATAAATTTCTATAAATTAGCACTATTCTTGTATT	8712
              ************************************************************

wildtype      ACAAAAAGCAAGGTACAGGTGACTACCCTAGTTCAAAACAACCACTTTCTCAGGCTTCTC	9000
mutant        ACAAAAAGCAAGGTACAGGTGACTACCCTAGTTCAAAACAACCACTTTCTCAGGCTTCTC	8772
              ************************************************************

wildtype      ATGTGTGTAGCTGAGCTACCTTGTCATATGTGTTGATTCTTGAAAACTGGGACACGTATT	9060
mutant        ATGTGTGTAGCTGAGCTACCTTGTCATATGTGTTGATTCTTGAAAACTGGGACACGTATT	8832
              ************************************************************

wildtype      TGCATTGGGGGTCAGCCCTTCACGCTGACGCACCAGCCTTCTAGCAGATGTACAGGAAAG	9120
mutant        TGCATTGGGGGTCAGCCCTTCACGCTGACGCACCAGCCTTCTAGCAGATGTACAGGAAAG	8892
              ************************************************************

wildtype      TGCTTTCAATCGGTGGACTGTCTTATTTTTTCAAATTTTTAAACTTTGCTTCTCCAAACA	9180
mutant        TGCTTTCAATCGGTGGACTGTCTTATTTTTTCAAATTTTTAAACTTTGCTTCTCCAAACA	8952
              ************************************************************

wildtype      CAAGTACTAGGTTGGCCAATTATAATATCTATTTGGTGCTAGTAAATTCTCAAACTAGAT	9240
mutant        CAAGTACTAGGTTGGCCAATTATAATATCTATTTGGTGCTAGTAAATTCTCAAACTAGAT	9012
              ************************************************************

wildtype      TTATAAATGCACTGTAATATTTACACAACTTAGAAACCAAATTACAAGTATTCAGTTCAA	9300
mutant        TTATAAATGCACTGTAATATTTACACAACTTAGAAACCAAATTACAAGTATTCAGTTCAA	9072
              ************************************************************

wildtype      ATACTTCATTAATTTCAATCAACCAAAGTTAGTTCAGTAGCTTATCTCAGTTATGAGTAT	9360
mutant        ATACTTCATTAATTTCAATCAACCAAAGTTAGTTCAGTAGCTTATCTCAGTTATGAGTAT	9132
              ************************************************************

wildtype      AATACATTACATGTAAATTAAGTGTGTGTATACTGTAATCGTGCTATTTTTTATCATTGA	9420
mutant        AATACATTACATGTAAATTAAGTGTGTGTATACTGTAATCGTGCTATTTTTTATCATTGA	9192
              ************************************************************

wildtype      AACATGTATAAACTAGAATAATAATGCCCTTAATGTGAGGGTTTGTAATGGTGCTTATTA	9480
mutant        AACATGTATAAACTAGAATAATAATGCCCTTAATGTGAGGGTTTGTAATGGTGCTTATTA	9252
              ************************************************************

wildtype      AGACCAAAGACTTGTTAAATGTATACACCAAGTGGTAAGGAAATTTCTGTGACTGGCCCA	9540
mutant        AGACCAAAGACTTGTTAAATGTATACACCAAGTGGTAAGGAAATTTCTGTGACTGGCCCA	9312
              ************************************************************

wildtype      CACGTGCATGGAGGTCTGGGAGGACCAAGAACCAGCCTCAGCAGCCAGAGGATCACCAGT	9600
mutant        CACGTGCATGGAGGTCTGGGAGGACCAAGAACCAGCCTCAGCAGCCAGAGGATCACCAGT	9372
              ************************************************************

wildtype      GCATCCTTCATCACACCTGTGCAATATGCCAAGATTACCCTCGGTCATTCCTATCAACAA	9660
mutant        GCATCCTTCATCACACCTGTGCAATATGCCAAGATTACCCTCGGTCATTCCTATCAACAA	9432
              ************************************************************

wildtype      GGGGTCCATGTCATAAATGTCACAATAAAACAGTCTCTCTTTTTTTTAGTGTA	9713
mutant        GGGGTCCATGTCATAAATGTCACAATAAAACAGTCTCTCTTTTTTTTAGTGTA	9485
              *****************************************************
```

C)

**M**RRGGLLEVVLGFTVLLASYTSHGADANLEAGNVKETRASRAKRRGGGGHDALKGPNVCGSRYN

AYCCPGWKTLPGGNQCIVPICRHSCGDGFCSRPNMCTCPSGQISPSCGSRSIQHCNIRCMNGGSC

SDDHCLCQKGYIGTHCGQPVCESGCLNGGRCVAPNRCACTYGFTGPQCERDYRTGPCFTVVSNQ

MCQGQLSGIVCTKTLCCATVGRAWGHPCEMCPAQPHPCRRGFIPNIRTGACQDVDECQAIPGLCQ

GGNCINTVGSFECKCPAGHKFNEASQKCEDIDECSTIPGICDGGECTNTVSSYFCKCPPGFYPSPD

GTRCVDVRPGYCYTALANGRCSNQLPQSITKMQCCCDVGRCWSPGVTVAPEMCPIRATEDFNKLC

SVPLVIPERPGYPPPPLGPIPPVHPVSPGFPPGPPVPVPRPPVEYPYPSREPPRVLPVNVTDYCQLF

RYLCQNGRCIPTPGSYRCECNKGFQLDLRGECIDVDECEKNPCAGGECINNQGSYTCQCRPGYQS

TLTRTECRDIDECLQNGRICNNGRCINTDGSFHCVCNAGFHVTRDGKNCEDMDECSIRNMCLNGM

CINEDGSFKCICKPGFQLASDGRYCKDINECETPGICMNGRCVNTDGSYRCECFPGLAVGLDGRVC

VDTHMRSTCYGGYKRGQCVKPLFGAVTKSECCCASTEYAFGEPCQPCPAQNSAEYQALCSSGPG

MTSAGSDINECALDPDICPNGICENLRGTYKCICNSGYEVDPSGKNCVDINECVLNSLLCDNGQCRN

TPGSFVCTCPKGFIYKPDLKTCEDIDECESSPCINGVCKNSPGSFICECSSESTLDPTKTICIETIKGTC

WQTVIDGRCEININGATLKSQCCSSLGAAWGSPCTLCQVDPICGKGYSRIKGTQCEDINECEVFPGV

CKNGVCVNSEGSFKCQCPNGMTLDATGRICLDIRLETCFLRYEDEECTLPVAGRHRMDACCCSVG

AAWGTEECEECPVRNTPEYEELCPRGPGFATKEITNGKPFFKDINECKMIPSLCTHGKCRNTIGSFK

CRCDSGFALDSEERNCTDIDECRISPDLCGRGQCVNTPGDFECKCDEGYESGFMMMKNCMDIDEC

QRDPLLCRGGVCLNTEGSYRCECPPGHQLSPNISACIDINECELSANLCPNGRCVNLIGKYQCACN

PGYHSTPDRLFCVDIDECSIMNGGCETFCTNSEGSYECSCQPGFALMPDQRSCTDIDECEDNPNIC

DGGQCTNIPGEYRCLCYDGFMASEDMKTCVDVNECDLNPNICLSGTCENTKGSFICHCDMGYSGK

KGKTGCTDINECEIGAHNCDRHAVCTNTAGSFKCSCSPGWIGDGIKCTDLDECSNGTHMCSQHAD

CKNTMGSYRCLCKEGYTGDGFTCTDLDECSENLNLCGNGQCLNAPGGYRCECDMGFVPSADGKA

CEDIDECSLPNICVFGTCHNLPGLFRCECEIGYELDRSGGNCTDVNECLDPTTCISGNCVNTPGSYT

CDCPPDFELNPTRVGCVDTRSGNCYLDIRPRGDNGDTACSNEIGVGVSKASCCCSLGKAWGTPCE

LCPPVNTSEYKILCPGGEGFRPNPITVILEDIDECQELPGLCQGGKCINTFGSFQCRCPTGYYLNEDT

RVCDDVNECETPGICGPGTCYNTVGNYTCICPPDYMQVNGGNNCMDMRRSLCYRNYYADNQTCD

GELLFNMTKKMCCCSYNIGRAWNKPCEQCPIPSTDEFATLCGSQRPGFVIDIYTGLPVDIDECREIP

GVCENGVCINMVGSFRCECPVGFFYNDKLLVCEDIDECQNGPVCQRNAECINTAGSFRCDCKPGY

RFTSTGQCNDRNECQEIPNICSHGQCIDTVGSFYCLCHTGFKTNADQTMCLDINECERDACGNGTC

RNTIGSFNCRCNHGFILSHNNDCIDVDECATGNGNLCRNGQCINTVGSFQCQCNEGYEVAPDGRT

CVDINECLLEPGKCAPGTCQNLDGSYRCICPPGYSLQNDKCEDIDECVEEPEICALGTCSNTEGSFK

CLCPDGFSLSSTGRRCQDLRMSYCYAKFEGGKCSSPKSRNHSKQECCCALKGEGWGDPCELCPT

EPDEAFRQICPYGSGIIVGPDDSAVDMDECKEPDVCKHGQCINTDGSYRCECPFGYILEGNECVDT

DECSVGNPCGNGTCKNVIGGFECTCEEGFEPGPMMTCEDINECAQNPLLCAFRCVNTYGSYECKC

PAGYVLREDRRMCKDEDECEEGKHDCAEKQMECKNLIGTYICICGPGYQRRPDGEGCVDENECQT

KPGICENGRCLNTRGSYTCECSDGFTPSPAQDECLDNREGYCFTEVLQNMCQIGSSNRNPVTKSE

CCCDGGRGWGPHCEICPFQGTVAFKKLCPHGRGFMTNGADIDECKVIHDVCRNGECVNDRGSYH

CLCKNGYTPDITGTSCVDLNECNQAPKPCNFICKNTGGSFQCSCPKGYILQEDGRSCKDLDECATK

QHNCQFLCVNTIGSFTCKCPPGFTQHHTACIDNNECTSDINLCGSKGICQNTPGSFTCECQRGFSL

DQSGASCEDVDECEGNHRCQHGCQNIIGGYRCSCPQGYLQHYQWNQCVDENECLSAHICGGASC

HNTLGSYKCMCPAGFQYEQFSGGCQDINECGSAQAPCSYGCSNTEGGYLCGCPPGYFRIGQGHC

VSGIGMGRGGSEPPASGEMDDNSLSPEACYECKINGYPKRGRKRRSANETDASNIEDQPERDTNV

SLASWDTEKTAMFAFNISHISNKVRILELLPALTTLTNHNKYLIESGNENGFFKINQKEGISYLHFTKKK

PVAGTYSLQISSTPLYKKKELNQLEDKYDKDYLSGELGDNLKMKIQILLH

D)

**M**RRGGLLEVVLGFTVLLASYTSHGADANLEAGNVKETRASRAKRRGGGGHDALKGPNVCGSRYN

AYCCPGWKTLPGGNQCIVPICRHSCGDGFCSRPNMCTCPSGQISPSCGSRSIQHCNIRCMNGGSC

SDDHCLCQKGYIGTHCGQPVCESGCLNGGRCVAPNRCACTYGFTGPQCERDYRTGPCFTVVSNQ

MCQGQLSGIVCTKTLCCATVGRAWGHPCEMCPAQPHPCRRGFIPNIRTGACQDVDECQAIPGLCQ

GGNCINTVGSFECKCPAGHKFNEASQKCEDIDECSTIPGICDGGECTNTVSSYFCKCPPGFYPSPD

GTRCVDVRPGYCYTALANGRCSNQLPQSITKMQCCCDVGRCWSPGVTVAPEMCPIRATEDFNKLC

SVPLVIPERPGYPPPPLGPIPPVHPVSPGFPPGPPVPVPRPPVEYPYPSREPPRVLPVNVTDYCQLF

RYLCQNGRCIPTPGSYRCECNKGFQLDLRGECIDVDECEKNPCAGGECINNQGSYTCQCRPGYQS

TLTRTECRDIDECLQNGRICNNGRCINTDGSFHCVCNAGFHVTRDGKNCEDMDECSIRNMCLNGM

CINEDGSFKCICKPGFQLASDGRYCKDINECETPGICMNGRCVNTDGSYRCECFPGLAVGLDGRVC

VDTHMRSTCYGGYKRGQCVKPLFGAVTKSECCCASTEYAFGEPCQPCPAQNSAEYQALCSSGPG

MTSAGSDINECALDPDICPNGICENLRGTYKCICNSGYEVDPSGKNCVDINECVLNSLLCDNGQCRN

TPGSFVCTCPKGFIYKPDLKTCEDIDECESSPCINGVCKNSPGSFICECSSESTLDPTKTICIETIKGTC

WQTVIDGRCEININGATLKSQCCSSLGAAWGSPCTLCQVDPICGKGYSRIKGTQCEDINECEVFPGV

CKNGVCVNSEGSFKCQCPNGMTLDATGRICLDINECKMIPSLCTHGKCRNTIGSFKCRCDSGFALD

SEERNCTDIDECRISPDLCGRGQCVNTPGDFECKCDEGYESGFMMMKNCMDIDECQRDPLLCRG

GVCLNTEGSYRCECPPGHQLSPNISACIDINECELSANLCPNGRCVNLIGKYQCACNPGYHSTPDRL

FCVDIDECSIMNGGCETFCTNSEGSYECSCQPGFALMPDQRSCTDIDECEDNPNICDGGQCTNIPG

EYRCLCYDGFMASEDMKTCVDVNECDLNPNICLSGTCENTKGSFICHCDMGYSGKKGKTGCTDIN

ECEIGAHNCDRHAVCTNTAGSFKCSCSPGWIGDGIKCTDLDECSNGTHMCSQHADCKNTMGSYR

CLCKEGYTGDGFTCTDLDECSENLNLCGNGQCLNAPGGYRCECDMGFVPSADGKACEDIDECSLP

NICVFGTCHNLPGLFRCECEIGYELDRSGGNCTDVNECLDPTTCISGNCVNTPGSYTCDCPPDFEL

NPTRVGCVDTRSGNCYLDIRPRGDNGDTACSNEIGVGVSKASCCCSLGKAWGTPCELCPPVNTSE

YKILCPGGEGFRPNPITVILEDIDECQELPGLCQGGKCINTFGSFQCRCPTGYYLNEDTRVCDDVNE

CETPGICGPGTCYNTVGNYTCICPPDYMQVNGGNNCMDMRRSLCYRNYYADNQTCDGELLFNMT

KKMCCCSYNIGRAWNKPCEQCPIPSTDEFATLCGSQRPGFVIDIYTGLPVDIDECREIPGVCENGVCI

NMVGSFRCECPVGFFYNDKLLVCEDIDECQNGPVCQRNAECINTAGSFRCDCKPGYRFTSTGQCN

DRNECQEIPNICSHGQCIDTVGSFYCLCHTGFKTNADQTMCLDINECERDACGNGTCRNTIGSFNC

RCNHGFILSHNNDCIDVDECATGNGNLCRNGQCINTVGSFQCQCNEGYEVAPDGRTCVDINECLLE

PGKCAPGTCQNLDGSYRCICPPGYSLQNDKCEDIDECVEEPEICALGTCSNTEGSFKCLCPDGFSL

SSTGRRCQDLRMSYCYAKFEGGKCSSPKSRNHSKQECCCALKGEGWGDPCELCPTEPDEAFRQI

CPYGSGIIVGPDDSAVDMDECKEPDVCKHGQCINTDGSYRCECPFGYILEGNECVDTDECSVGNPC

GNGTCKNVIGGFECTCEEGFEPGPMMTCEDINECAQNPLLCAFRCVNTYGSYECKCPAGYVLRED

RRMCKDEDECEEGKHDCAEKQMECKNLIGTYICICGPGYQRRPDGEGCVDENECQTKPGICENGR

CLNTRGSYTCECSDGFTPSPAQDECLDNREGYCFTEVLQNMCQIGSSNRNPVTKSECCCDGGRG

WGPHCEICPFQGTVAFKKLCPHGRGFMTNGADIDECKVIHDVCRNGECVNDRGSYHCLCKNGYTP

DITGTSCVDLNECNQAPKPCNFICKNTGGSFQCSCPKGYILQEDGRSCKDLDECATKQHNCQFLCV

NTIGSFTCKCPPGFTQHHTACIDNNECTSDINLCGSKGICQNTPGSFTCECQRGFSLDQSGASCED

VDECEGNHRCQHGCQNIIGGYRCSCPQGYLQHYQWNQCVDENECLSAHICGGASCHNTLGSYKC

MCPAGFQYEQFSGGCQDINECGSAQAPCSYGCSNTEGGYLCGCPPGYFRIGQGHCVSGIGMGRG

GSEPPASGEMDDNSLSPEACYECKINGYPKRGRKRRSANETDASNIEDQPERDTNVSLASWDTEK

TAMFAFNISHISNKVRILELLPALTTLTNHNKYLIESGNENGFFKINQKEGISYLHFTKKKPVAGTYSLQ

ISSTPLYKKKELNQLEDKYDKDYLSGELGDNLKMKIQILLH

E)

```
CLUSTAL O(1.2.4) multiple sequence alignment


wildtype      MRRGGLLEVVLGFTVLLASYTSHGADANLEAGNVKETRASRAKRRGGGGHDALKGPNVCG	60
mutant        MRRGGLLEVVLGFTVLLASYTSHGADANLEAGNVKETRASRAKRRGGGGHDALKGPNVCG	60
              ************************************************************

wildtype      SRYNAYCCPGWKTLPGGNQCIVPICRHSCGDGFCSRPNMCTCPSGQISPSCGSRSIQHCN	120
mutant        SRYNAYCCPGWKTLPGGNQCIVPICRHSCGDGFCSRPNMCTCPSGQISPSCGSRSIQHCN	120
              ************************************************************

wildtype      IRCMNGGSCSDDHCLCQKGYIGTHCGQPVCESGCLNGGRCVAPNRCACTYGFTGPQCERD	180
mutant        IRCMNGGSCSDDHCLCQKGYIGTHCGQPVCESGCLNGGRCVAPNRCACTYGFTGPQCERD	180
              ************************************************************

wildtype      YRTGPCFTVVSNQMCQGQLSGIVCTKTLCCATVGRAWGHPCEMCPAQPHPCRRGFIPNIR	240
mutant        YRTGPCFTVVSNQMCQGQLSGIVCTKTLCCATVGRAWGHPCEMCPAQPHPCRRGFIPNIR	240
              ************************************************************

wildtype      TGACQDVDECQAIPGLCQGGNCINTVGSFECKCPAGHKFNEASQKCEDIDECSTIPGICD	300
mutant        TGACQDVDECQAIPGLCQGGNCINTVGSFECKCPAGHKFNEASQKCEDIDECSTIPGICD	300
              ************************************************************

wildtype      GGECTNTVSSYFCKCPPGFYPSPDGTRCVDVRPGYCYTALANGRCSNQLPQSITKMQCCC	360
mutant        GGECTNTVSSYFCKCPPGFYPSPDGTRCVDVRPGYCYTALANGRCSNQLPQSITKMQCCC	360
              ************************************************************

wildtype      DVGRCWSPGVTVAPEMCPIRATEDFNKLCSVPLVIPERPGYPPPPLGPIPPVHPVSPGFP	420
mutant        DVGRCWSPGVTVAPEMCPIRATEDFNKLCSVPLVIPERPGYPPPPLGPIPPVHPVSPGFP	420
              ************************************************************

wildtype      PGPPVPVPRPPVEYPYPSREPPRVLPVNVTDYCQLFRYLCQNGRCIPTPGSYRCECNKGF	480
mutant        PGPPVPVPRPPVEYPYPSREPPRVLPVNVTDYCQLFRYLCQNGRCIPTPGSYRCECNKGF	480
              ************************************************************

wildtype      QLDLRGECIDVDECEKNPCAGGECINNQGSYTCQCRPGYQSTLTRTECRDIDECLQNGRI	540
mutant        QLDLRGECIDVDECEKNPCAGGECINNQGSYTCQCRPGYQSTLTRTECRDIDECLQNGRI	540
              ************************************************************

wildtype      CNNGRCINTDGSFHCVCNAGFHVTRDGKNCEDMDECSIRNMCLNGMCINEDGSFKCICKP	600
mutant        CNNGRCINTDGSFHCVCNAGFHVTRDGKNCEDMDECSIRNMCLNGMCINEDGSFKCICKP	600
              ************************************************************

wildtype      GFQLASDGRYCKDINECETPGICMNGRCVNTDGSYRCECFPGLAVGLDGRVCVDTHMRST	660
mutant        GFQLASDGRYCKDINECETPGICMNGRCVNTDGSYRCECFPGLAVGLDGRVCVDTHMRST	660
              ************************************************************

wildtype      CYGGYKRGQCVKPLFGAVTKSECCCASTEYAFGEPCQPCPAQNSAEYQALCSSGPGMTSA	720
mutant        CYGGYKRGQCVKPLFGAVTKSECCCASTEYAFGEPCQPCPAQNSAEYQALCSSGPGMTSA	720
              ************************************************************

wildtype      GSDINECALDPDICPNGICENLRGTYKCICNSGYEVDPSGKNCVDINECVLNSLLCDNGQ	780
mutant        GSDINECALDPDICPNGICENLRGTYKCICNSGYEVDPSGKNCVDINECVLNSLLCDNGQ	780
              ************************************************************

wildtype      CRNTPGSFVCTCPKGFIYKPDLKTCEDIDECESSPCINGVCKNSPGSFICECSSESTLDP	840
mutant        CRNTPGSFVCTCPKGFIYKPDLKTCEDIDECESSPCINGVCKNSPGSFICECSSESTLDP	840
              ************************************************************

wildtype      TKTICIETIKGTCWQTVIDGRCEININGATLKSQCCSSLGAAWGSPCTLCQVDPICGKGY	900
mutant        TKTICIETIKGTCWQTVIDGRCEININGATLKSQCCSSLGAAWGSPCTLCQVDPICGKGY	900
              ************************************************************

wildtype      SRIKGTQCEDINECEVFPGVCKNGVCVNSEGSFKCQCPNGMTLDATGRICLDIRLETCFL	960
mutant        SRIKGTQCEDINECEVFPGVCKNGVCVNSEGSFKCQCPNGMTLDATGRICLD--------	952
              ****************************************************

wildtype      RYEDEECTLPVAGRHRMDACCCSVGAAWGTEECEECPVRNTPEYEELCPRGPGFATKEIT	1020
mutant        ------------------------------------------------------------	952


wildtype      NGKPFFKDINECKMIPSLCTHGKCRNTIGSFKCRCDSGFALDSEERNCTDIDECRISPDL	1080
mutant        --------INECKMIPSLCTHGKCRNTIGSFKCRCDSGFALDSEERNCTDIDECRISPDL	1004
                      ****************************************************

wildtype      CGRGQCVNTPGDFECKCDEGYESGFMMMKNCMDIDECQRDPLLCRGGVCLNTEGSYRCEC	1140
mutant        CGRGQCVNTPGDFECKCDEGYESGFMMMKNCMDIDECQRDPLLCRGGVCLNTEGSYRCEC	1064
              ************************************************************

wildtype      PPGHQLSPNISACIDINECELSANLCPNGRCVNLIGKYQCACNPGYHSTPDRLFCVDIDE	1200
mutant        PPGHQLSPNISACIDINECELSANLCPNGRCVNLIGKYQCACNPGYHSTPDRLFCVDIDE	1124
              ************************************************************

wildtype      CSIMNGGCETFCTNSEGSYECSCQPGFALMPDQRSCTDIDECEDNPNICDGGQCTNIPGE	1260
mutant        CSIMNGGCETFCTNSEGSYECSCQPGFALMPDQRSCTDIDECEDNPNICDGGQCTNIPGE	1184
              ************************************************************

wildtype      YRCLCYDGFMASEDMKTCVDVNECDLNPNICLSGTCENTKGSFICHCDMGYSGKKGKTGC	1320
mutant        YRCLCYDGFMASEDMKTCVDVNECDLNPNICLSGTCENTKGSFICHCDMGYSGKKGKTGC	1244
              ************************************************************

wildtype      TDINECEIGAHNCDRHAVCTNTAGSFKCSCSPGWIGDGIKCTDLDECSNGTHMCSQHADC	1380
mutant        TDINECEIGAHNCDRHAVCTNTAGSFKCSCSPGWIGDGIKCTDLDECSNGTHMCSQHADC	1304
              ************************************************************

wildtype      KNTMGSYRCLCKEGYTGDGFTCTDLDECSENLNLCGNGQCLNAPGGYRCECDMGFVPSAD	1440
mutant        KNTMGSYRCLCKEGYTGDGFTCTDLDECSENLNLCGNGQCLNAPGGYRCECDMGFVPSAD	1364
              ************************************************************

wildtype      GKACEDIDECSLPNICVFGTCHNLPGLFRCECEIGYELDRSGGNCTDVNECLDPTTCISG	1500
mutant        GKACEDIDECSLPNICVFGTCHNLPGLFRCECEIGYELDRSGGNCTDVNECLDPTTCISG	1424
              ************************************************************

wildtype      NCVNTPGSYTCDCPPDFELNPTRVGCVDTRSGNCYLDIRPRGDNGDTACSNEIGVGVSKA	1560
mutant        NCVNTPGSYTCDCPPDFELNPTRVGCVDTRSGNCYLDIRPRGDNGDTACSNEIGVGVSKA	1484
              ************************************************************

wildtype      SCCCSLGKAWGTPCELCPPVNTSEYKILCPGGEGFRPNPITVILEDIDECQELPGLCQGG	1620
mutant        SCCCSLGKAWGTPCELCPPVNTSEYKILCPGGEGFRPNPITVILEDIDECQELPGLCQGG	1544
              ************************************************************

wildtype      KCINTFGSFQCRCPTGYYLNEDTRVCDDVNECETPGICGPGTCYNTVGNYTCICPPDYMQ	1680
mutant        KCINTFGSFQCRCPTGYYLNEDTRVCDDVNECETPGICGPGTCYNTVGNYTCICPPDYMQ	1604
              ************************************************************

wildtype      VNGGNNCMDMRRSLCYRNYYADNQTCDGELLFNMTKKMCCCSYNIGRAWNKPCEQCPIPS	1740
mutant        VNGGNNCMDMRRSLCYRNYYADNQTCDGELLFNMTKKMCCCSYNIGRAWNKPCEQCPIPS	1664
              ************************************************************

wildtype      TDEFATLCGSQRPGFVIDIYTGLPVDIDECREIPGVCENGVCINMVGSFRCECPVGFFYN	1800
mutant        TDEFATLCGSQRPGFVIDIYTGLPVDIDECREIPGVCENGVCINMVGSFRCECPVGFFYN	1724
              ************************************************************

wildtype      DKLLVCEDIDECQNGPVCQRNAECINTAGSFRCDCKPGYRFTSTGQCNDRNECQEIPNIC	1860
mutant        DKLLVCEDIDECQNGPVCQRNAECINTAGSFRCDCKPGYRFTSTGQCNDRNECQEIPNIC	1784
              ************************************************************

wildtype      SHGQCIDTVGSFYCLCHTGFKTNADQTMCLDINECERDACGNGTCRNTIGSFNCRCNHGF	1920
mutant        SHGQCIDTVGSFYCLCHTGFKTNADQTMCLDINECERDACGNGTCRNTIGSFNCRCNHGF	1844
              ************************************************************

wildtype      ILSHNNDCIDVDECATGNGNLCRNGQCINTVGSFQCQCNEGYEVAPDGRTCVDINECLLE	1980
mutant        ILSHNNDCIDVDECATGNGNLCRNGQCINTVGSFQCQCNEGYEVAPDGRTCVDINECLLE	1904
              ************************************************************

wildtype      PGKCAPGTCQNLDGSYRCICPPGYSLQNDKCEDIDECVEEPEICALGTCSNTEGSFKCLC	2040
mutant        PGKCAPGTCQNLDGSYRCICPPGYSLQNDKCEDIDECVEEPEICALGTCSNTEGSFKCLC	1964
              ************************************************************

wildtype      PDGFSLSSTGRRCQDLRMSYCYAKFEGGKCSSPKSRNHSKQECCCALKGEGWGDPCELCP	2100
mutant        PDGFSLSSTGRRCQDLRMSYCYAKFEGGKCSSPKSRNHSKQECCCALKGEGWGDPCELCP	2024
              ************************************************************

wildtype      TEPDEAFRQICPYGSGIIVGPDDSAVDMDECKEPDVCKHGQCINTDGSYRCECPFGYILE	2160
mutant        TEPDEAFRQICPYGSGIIVGPDDSAVDMDECKEPDVCKHGQCINTDGSYRCECPFGYILE	2084
              ************************************************************

wildtype      GNECVDTDECSVGNPCGNGTCKNVIGGFECTCEEGFEPGPMMTCEDINECAQNPLLCAFR	2220
mutant        GNECVDTDECSVGNPCGNGTCKNVIGGFECTCEEGFEPGPMMTCEDINECAQNPLLCAFR	2144
              ************************************************************

wildtype      CVNTYGSYECKCPAGYVLREDRRMCKDEDECEEGKHDCAEKQMECKNLIGTYICICGPGY	2280
mutant        CVNTYGSYECKCPAGYVLREDRRMCKDEDECEEGKHDCAEKQMECKNLIGTYICICGPGY	2204
              ************************************************************

wildtype      QRRPDGEGCVDENECQTKPGICENGRCLNTRGSYTCECSDGFTPSPAQDECLDNREGYCF	2340
mutant        QRRPDGEGCVDENECQTKPGICENGRCLNTRGSYTCECSDGFTPSPAQDECLDNREGYCF	2264
              ************************************************************

wildtype      TEVLQNMCQIGSSNRNPVTKSECCCDGGRGWGPHCEICPFQGTVAFKKLCPHGRGFMTNG	2400
mutant        TEVLQNMCQIGSSNRNPVTKSECCCDGGRGWGPHCEICPFQGTVAFKKLCPHGRGFMTNG	2324
              ************************************************************

wildtype      ADIDECKVIHDVCRNGECVNDRGSYHCLCKNGYTPDITGTSCVDLNECNQAPKPCNFICK	2460
mutant        ADIDECKVIHDVCRNGECVNDRGSYHCLCKNGYTPDITGTSCVDLNECNQAPKPCNFICK	2384
              ************************************************************

wildtype      NTGGSFQCSCPKGYILQEDGRSCKDLDECATKQHNCQFLCVNTIGSFTCKCPPGFTQHHT	2520
mutant        NTGGSFQCSCPKGYILQEDGRSCKDLDECATKQHNCQFLCVNTIGSFTCKCPPGFTQHHT	2444
              ************************************************************

wildtype      ACIDNNECTSDINLCGSKGICQNTPGSFTCECQRGFSLDQSGASCEDVDECEGNHRCQHG	2580
mutant        ACIDNNECTSDINLCGSKGICQNTPGSFTCECQRGFSLDQSGASCEDVDECEGNHRCQHG	2504
              ************************************************************

wildtype      CQNIIGGYRCSCPQGYLQHYQWNQCVDENECLSAHICGGASCHNTLGSYKCMCPAGFQYE	2640
mutant        CQNIIGGYRCSCPQGYLQHYQWNQCVDENECLSAHICGGASCHNTLGSYKCMCPAGFQYE	2564
              ************************************************************

wildtype      QFSGGCQDINECGSAQAPCSYGCSNTEGGYLCGCPPGYFRIGQGHCVSGIGMGRGGSEPP	2700
mutant        QFSGGCQDINECGSAQAPCSYGCSNTEGGYLCGCPPGYFRIGQGHCVSGIGMGRGGSEPP	2624
              ************************************************************

wildtype      ASGEMDDNSLSPEACYECKINGYPKRGRKRRSANETDASNIEDQPERDTNVSLASWDTEK	2760
mutant        ASGEMDDNSLSPEACYECKINGYPKRGRKRRSANETDASNIEDQPERDTNVSLASWDTEK	2684
              ************************************************************

wildtype      TAMFAFNISHISNKVRILELLPALTTLTNHNKYLIESGNENGFFKINQKEGISYLHFTKK	2820
mutant        TAMFAFNISHISNKVRILELLPALTTLTNHNKYLIESGNENGFFKINQKEGISYLHFTKK	2744
              ************************************************************

wildtype      KPVAGTYSLQISSTPLYKKKELNQLEDKYDKDYLSGELGDNLKMKIQILLH	2871
mutant        KPVAGTYSLQISSTPLYKKKELNQLEDKYDKDYLSGELGDNLKMKIQILLH	2795
              ***************************************************
```

F) Fibrillin 1 Precursor Protein (NP_001274014.2) Sequence Alignment:

```
CLUSTAL O(1.2.4) multiple sequence alignment


wildtype      MRRGGLLEVVLGFTVLLASYTSHGADANLEAGNVKETRASRAKRRGGGGHDALKGPNVCG	60
mutant        MRRGGLLEVVLGFTVLLASYTSHGADANLEAGNVKETRASRAKRRGGGGHDALKGPNVCG	60
              ************************************************************

wildtype      SRYNAYCCPGWKTLPGGNQCIVPICRHSCGDGFCSRPNMCTCPSGQISPSCGSRSIQHCN	120
mutant        SRYNAYCCPGWKTLPGGNQCIVPICRHSCGDGFCSRPNMCTCPSGQISPSCGSRSIQHCN	120
              ************************************************************

wildtype      IRCMNGGSCSDDHCLCQKGYIGTHCGQPVCESGCLNGGRCVAPNRCACTYGFTGPQCERD	180
mutant        IRCMNGGSCSDDHCLCQKGYIGTHCGQPVCESGCLNGGRCVAPNRCACTYGFTGPQCERD	180
              ************************************************************

wildtype      YRTGPCFTVVSNQMCQGQLSGIVCTKTLCCATVGRAWGHPCEMCPAQPHPCRRGFIPNIR	240
mutant        YRTGPCFTVVSNQMCQGQLSGIVCTKTLCCATVGRAWGHPCEMCPAQPHPCRRGFIPNIR	240
              ************************************************************

wildtype      TGACQDVDECQAIPGLCQGGNCINTVGSFECKCPAGHKFNEASQKCEDIDECSTIPGICD	300
mutant        TGACQDVDECQAIPGLCQGGNCINTVGSFECKCPAGHKFNEASQKCEDIDECSTIPGICD	300
              ************************************************************

wildtype      GGECTNTVSSYFCKCPPGFYPSPDGTRCVDVRPGYCYTALANGRCSNQLPQSITKMQCCC	360
mutant        GGECTNTVSSYFCKCPPGFYPSPDGTRCVDVRPGYCYTALANGRCSNQLPQSITKMQCCC	360
              ************************************************************

wildtype      DVGRCWSPGVTVAPEMCPIRATEDFNKLCSVPLVIPERPGYPPPPLGPIPPVHPVSPGFP	420
mutant        DVGRCWSPGVTVAPEMCPIRATEDFNKLCSVPLVIPERPGYPPPPLGPIPPVHPVSPGFP	420
              ************************************************************

wildtype      PGPPVPVPRPPVEYPYPSREPPRVLPVNVTDYCQLFRYLCQNGRCIPTPGSYRCECNKGF	480
mutant        PGPPVPVPRPPVEYPYPSREPPRVLPVNVTDYCQLFRYLCQNGRCIPTPGSYRCECNKGF	480
              ************************************************************

wildtype      QLDLRGECIDVDECEKNPCAGGECINNQGSYTCQCRPGYQSTLTRTECRDIDECLQNGRI	540
mutant        QLDLRGECIDVDECEKNPCAGGECINNQGSYTCQCRPGYQSTLTRTECRDIDECLQNGRI	540
              ************************************************************

wildtype      CNNGRCINTDGSFHCVCNAGFHVTRDGKNCEDMDECSIRNMCLNGMCINEDGSFKCICKP	600
mutant        CNNGRCINTDGSFHCVCNAGFHVTRDGKNCEDMDECSIRNMCLNGMCINEDGSFKCICKP	600
              ************************************************************

wildtype      GFQLASDGRYCKDINECETPGICMNGRCVNTDGSYRCECFPGLAVGLDGRVCVDTHMRST	660
mutant        GFQLASDGRYCKDINECETPGICMNGRCVNTDGSYRCECFPGLAVGLDGRVCVDTHMRST	660
              ************************************************************

wildtype      CYGGYKRGQCVKPLFGAVTKSECCCASTEYAFGEPCQPCPAQNSAEYQALCSSGPGMTSA	720
mutant        CYGGYKRGQCVKPLFGAVTKSECCCASTEYAFGEPCQPCPAQNSAEYQALCSSGPGMTSA	720
              ************************************************************

wildtype      GSDINECALDPDICPNGICENLRGTYKCICNSGYEVDPSGKNCVDINECVLNSLLCDNGQ	780
mutant        GSDINECALDPDICPNGICENLRGTYKCICNSGYEVDPSGKNCVDINECVLNSLLCDNGQ	780
              ************************************************************

wildtype      CRNTPGSFVCTCPKGFIYKPDLKTCEDIDECESSPCINGVCKNSPGSFICECSSESTLDP	840
mutant        CRNTPGSFVCTCPKGFIYKPDLKTCEDIDECESSPCINGVCKNSPGSFICECSSESTLDP	840
              ************************************************************

wildtype      TKTICIETIKGTCWQTVIDGRCEININGATLKSQCCSSLGAAWGSPCTLCQVDPICGKGY	900
mutant        TKTICIETIKGTCWQTVIDGRCEININGATLKSQCCSSLGAAWGSPCTLCQVDPICGKGY	900
              ************************************************************

wildtype      SRIKGTQCEDINECEVFPGVCKNGVCVNSEGSFKCQCPNGMTLDATGRICLDIRLETCFL	960
mutant        SRIKGTQCEDINECEVFPGVCKNGVCVNSEGSFKCQCPNGMTLDATGRICLD--------	952
              ****************************************************

wildtype      RYEDEECTLPVAGRHRMDACCCSVGAAWGTEECEECPVRNTPEYEELCPRGPGFATKEIT	1020
mutant        ------------------------------------------------------------	952


wildtype      NGKPFFKDINECKMIPSLCTHGKCRNTIGSFKCRCDSGFALDSEERNCTDIDECRISPDL	1080
mutant        --------INECKMIPSLCTHGKCRNTIGSFKCRCDSGFALDSEERNCTDIDECRISPDL	1004
                      ****************************************************

wildtype      CGRGQCVNTPGDFECKCDEGYESGFMMMKNCMDIDECQRDPLLCRGGVCLNTEGSYRCEC	1140
mutant        CGRGQCVNTPGDFECKCDEGYESGFMMMKNCMDIDECQRDPLLCRGGVCLNTEGSYRCEC	1064
              ************************************************************

wildtype      PPGHQLSPNISACIDINECELSANLCPNGRCVNLIGKYQCACNPGYHSTPDRLFCVDIDE	1200
mutant        PPGHQLSPNISACIDINECELSANLCPNGRCVNLIGKYQCACNPGYHSTPDRLFCVDIDE	1124
              ************************************************************

wildtype      CSIMNGGCETFCTNSEGSYECSCQPGFALMPDQRSCTDIDECEDNPNICDGGQCTNIPGE	1260
mutant        CSIMNGGCETFCTNSEGSYECSCQPGFALMPDQRSCTDIDECEDNPNICDGGQCTNIPGE	1184
              ************************************************************

wildtype      YRCLCYDGFMASEDMKTCVDVNECDLNPNICLSGTCENTKGSFICHCDMGYSGKKGKTGC	1320
mutant        YRCLCYDGFMASEDMKTCVDVNECDLNPNICLSGTCENTKGSFICHCDMGYSGKKGKTGC	1244
              ************************************************************

wildtype      TDINECEIGAHNCDRHAVCTNTAGSFKCSCSPGWIGDGIKCTDLDECSNGTHMCSQHADC	1380
mutant        TDINECEIGAHNCDRHAVCTNTAGSFKCSCSPGWIGDGIKCTDLDECSNGTHMCSQHADC	1304
              ************************************************************

wildtype      KNTMGSYRCLCKEGYTGDGFTCTDLDECSENLNLCGNGQCLNAPGGYRCECDMGFVPSAD	1440
mutant        KNTMGSYRCLCKEGYTGDGFTCTDLDECSENLNLCGNGQCLNAPGGYRCECDMGFVPSAD	1364
              ************************************************************

wildtype      GKACEDIDECSLPNICVFGTCHNLPGLFRCECEIGYELDRSGGNCTDVNECLDPTTCISG	1500
mutant        GKACEDIDECSLPNICVFGTCHNLPGLFRCECEIGYELDRSGGNCTDVNECLDPTTCISG	1424
              ************************************************************

wildtype      NCVNTPGSYTCDCPPDFELNPTRVGCVDTRSGNCYLDIRPRGDNGDTACSNEIGVGVSKA	1560
mutant        NCVNTPGSYTCDCPPDFELNPTRVGCVDTRSGNCYLDIRPRGDNGDTACSNEIGVGVSKA	1484
              ************************************************************

wildtype      SCCCSLGKAWGTPCELCPPVNTSEYKILCPGGEGFRPNPITVILEDIDECQELPGLCQGG	1620
mutant        SCCCSLGKAWGTPCELCPPVNTSEYKILCPGGEGFRPNPITVILEDIDECQELPGLCQGG	1544
              ************************************************************

wildtype      KCINTFGSFQCRCPTGYYLNEDTRVCDDVNECETPGICGPGTCYNTVGNYTCICPPDYMQ	1680
mutant        KCINTFGSFQCRCPTGYYLNEDTRVCDDVNECETPGICGPGTCYNTVGNYTCICPPDYMQ	1604
              ************************************************************

wildtype      VNGGNNCMDMRRSLCYRNYYADNQTCDGELLFNMTKKMCCCSYNIGRAWNKPCEQCPIPS	1740
mutant        VNGGNNCMDMRRSLCYRNYYADNQTCDGELLFNMTKKMCCCSYNIGRAWNKPCEQCPIPS	1664
              ************************************************************

wildtype      TDEFATLCGSQRPGFVIDIYTGLPVDIDECREIPGVCENGVCINMVGSFRCECPVGFFYN	1800
mutant        TDEFATLCGSQRPGFVIDIYTGLPVDIDECREIPGVCENGVCINMVGSFRCECPVGFFYN	1724
              ************************************************************

wildtype      DKLLVCEDIDECQNGPVCQRNAECINTAGSFRCDCKPGYRFTSTGQCNDRNECQEIPNIC	1860
mutant        DKLLVCEDIDECQNGPVCQRNAECINTAGSFRCDCKPGYRFTSTGQCNDRNECQEIPNIC	1784
              ************************************************************

wildtype      SHGQCIDTVGSFYCLCHTGFKTNADQTMCLDINECERDACGNGTCRNTIGSFNCRCNHGF	1920
mutant        SHGQCIDTVGSFYCLCHTGFKTNADQTMCLDINECERDACGNGTCRNTIGSFNCRCNHGF	1844
              ************************************************************

wildtype      ILSHNNDCIDVDECATGNGNLCRNGQCINTVGSFQCQCNEGYEVAPDGRTCVDINECLLE	1980
mutant        ILSHNNDCIDVDECATGNGNLCRNGQCINTVGSFQCQCNEGYEVAPDGRTCVDINECLLE	1904
              ************************************************************

wildtype      PGKCAPGTCQNLDGSYRCICPPGYSLQNDKCEDIDECVEEPEICALGTCSNTEGSFKCLC	2040
mutant        PGKCAPGTCQNLDGSYRCICPPGYSLQNDKCEDIDECVEEPEICALGTCSNTEGSFKCLC	1964
              ************************************************************

wildtype      PDGFSLSSTGRRCQDLRMSYCYAKFEGGKCSSPKSRNHSKQECCCALKGEGWGDPCELCP	2100
mutant        PDGFSLSSTGRRCQDLRMSYCYAKFEGGKCSSPKSRNHSKQECCCALKGEGWGDPCELCP	2024
              ************************************************************

wildtype      TEPDEAFRQICPYGSGIIVGPDDSAVDMDECKEPDVCKHGQCINTDGSYRCECPFGYILE	2160
mutant        TEPDEAFRQICPYGSGIIVGPDDSAVDMDECKEPDVCKHGQCINTDGSYRCECPFGYILE	2084
              ************************************************************

wildtype      GNECVDTDECSVGNPCGNGTCKNVIGGFECTCEEGFEPGPMMTCEDINECAQNPLLCAFR	2220
mutant        GNECVDTDECSVGNPCGNGTCKNVIGGFECTCEEGFEPGPMMTCEDINECAQNPLLCAFR	2144
              ************************************************************

wildtype      CVNTYGSYECKCPAGYVLREDRRMCKDEDECEEGKHDCAEKQMECKNLIGTYICICGPGY	2280
mutant        CVNTYGSYECKCPAGYVLREDRRMCKDEDECEEGKHDCAEKQMECKNLIGTYICICGPGY	2204
              ************************************************************

wildtype      QRRPDGEGCVDENECQTKPGICENGRCLNTRGSYTCECSDGFTPSPAQDECLDNREGYCF	2340
mutant        QRRPDGEGCVDENECQTKPGICENGRCLNTRGSYTCECSDGFTPSPAQDECLDNREGYCF	2264
              ************************************************************

wildtype      TEVLQNMCQIGSSNRNPVTKSECCCDGGRGWGPHCEICPFQGTVAFKKLCPHGRGFMTNG	2400
mutant        TEVLQNMCQIGSSNRNPVTKSECCCDGGRGWGPHCEICPFQGTVAFKKLCPHGRGFMTNG	2324
              ************************************************************

wildtype      ADIDECKVIHDVCRNGECVNDRGSYHCLCKNGYTPDITGTSCVDLNECNQAPKPCNFICK	2460
mutant        ADIDECKVIHDVCRNGECVNDRGSYHCLCKNGYTPDITGTSCVDLNECNQAPKPCNFICK	2384
              ************************************************************

wildtype      NTGGSFQCSCPKGYILQEDGRSCKDLDECATKQHNCQFLCVNTIGSFTCKCPPGFTQHHT	2520
mutant        NTGGSFQCSCPKGYILQEDGRSCKDLDECATKQHNCQFLCVNTIGSFTCKCPPGFTQHHT	2444
              ************************************************************

wildtype      ACIDNNECTSDINLCGSKGICQNTPGSFTCECQRGFSLDQSGASCEDVDECEGNHRCQHG	2580
mutant        ACIDNNECTSDINLCGSKGICQNTPGSFTCECQRGFSLDQSGASCEDVDECEGNHRCQHG	2504
              ************************************************************

wildtype      CQNIIGGYRCSCPQGYLQHYQWNQCVDENECLSAHICGGASCHNTLGSYKCMCPAGFQYE	2640
mutant        CQNIIGGYRCSCPQGYLQHYQWNQCVDENECLSAHICGGASCHNTLGSYKCMCPAGFQYE	2564
              ************************************************************

wildtype      QFSGGCQDINECGSAQAPCSYGCSNTEGGYLCGCPPGYFRIGQGHCVSGIGMGRGGSEPP	2700
mutant        QFSGGCQDINECGSAQAPCSYGCSNTEGGYLCGCPPGYFRIGQGHCVSGIGMGRGGSEPP	2624
              ************************************************************

wildtype      ASGEMDDNSLSPEACYECKINGYPKRGRKRRSANETDASNIEDQPERDTNVSLASWDTEK	2760
mutant        ASGEMDDNSLSPEACYECKINGYPKRGRKRRSANETDASNIEDQPERDTNVSLASWDTEK	2684
              ************************************************************

wildtype      TAMFAFNISHISNKVRILELLPALTTLTNHNKYLIESGNENGFFKINQKEGISYLHFTKK	2820
mutant        TAMFAFNISHISNKVRILELLPALTTLTNHNKYLIESGNENGFFKINQKEGISYLHFTKK	2744
              ************************************************************

wildtype      KPVAGTYSLQISSTPLYKKKELNQLEDKYDKDYLSGELGDNLKMKIQILLH	2871
mutant        KPVAGTYSLQISSTPLYKKKELNQLEDKYDKDYLSGELGDNLKMKIQILLH	2795
              ***************************************************
```
