# Supplementary material for: Clinical and molecular genetic description of a Marfan-like syndrome in a Golden Retriever puppy
Source: J Vet Intern Med. 2026 Sep 30;40(5):aalag222. doi: 10.1093/jvimsj/aalag222 (PMC13626598; doi:10.1093/jvimsj/aalag222)
Supplement: Supplementary file 1 [file supplementary_material_aalag222.zip › File S_5.docx]

**File S4: As a supplementary file, we report a brief investigation supporting localized mosaicism for the proposed causative deletion in the affected individual, as elaborated below.**

**Materials and Methods**
To investigate whether the low-frequency third-allele SNV signals observed within the deleted interval was a result of artifacts or reflected instead chimerism/mosaicism, we performed a site-level allele-balance analysis using sample-level allelic depths from a multi-sample VCF. Across the genome, we identified loci with markedly skewed minor-allele fractions. Then, we compared the distribution of skewed sites in the proband with that observed in 36 informative internal control dogs and specifically assessed whether the proband showed an overall greater burden of allelic imbalance compared to the controls. Analyses were performed using two custom scripts deposited in Zenodo [https://doi.org/10.5281/zenodo.20126484].

File S_4_Table1: Partial validation of candidate mosaicism variants through Sanger sequencing was carried out using the following primers:

| **Primer name** | **Primer sequence** | **Position (canFam4)** | **Role** |
| --- | --- | --- | --- |
| Mosaic_12_F | 5’-CCGTGTCAACCAACCAAGAG-3’ | chr30:14998516-14998535 | Genotyping potential mosaicism inside of the deleted interval |
| Mosaic_12_R | 5’-TGAGTGTTTAATCCAAGAAGCCC-3’ | chr30:14999007-14999029 | Genotyping potential mosaicism inside of the deleted interval |
| Mosaic_3_F | 5’-CTGCCACTAAGAACGCACAG-3’ | chr30:15001573-15001592 | Genotyping potential mosaicism inside of the deleted interval |
| Mosaic_3_R | 5’-TGTGGGTGTGAGTGAAGAGT-3’ | chr30:15001733-15001752 | Genotyping potential mosaicism inside of the deleted interval |
| Genomic_1_F | 5’-GGCATCTCTCAGAGCAGGAT-3’ | chr16:58115066-58115085 | Genotyping potential mosaicism out of the deleted interval |
| Genomic_1_R | 5’-GTGATTGCTTCCCTTGCTGT-3’ | chr16:58115258-58115277 | Genotyping potential mosaicism out of the deleted interval |
| Genomic_2_F | 5’-CAGTGAGACCAAGAAAATTTGCA-3’ | chr21:18364391-18364413 | Genotyping potential mosaicism out of the deleted interval |
| Genomic_2_R | 5’-TTATGTCTGAAGGCCACCGT-3’ | chr21:18364615-18364634 | Genotyping potential mosaicism out of the deleted interval |

PCR parameters were the same for all primers: 95C x10’ (95C x30”denaturation, 59C x30” annealing, 72C x30” extension) x32 cycles, 72C x7’.

**Results**

Visual inspection of WGS data highlighted the presence of heterozygous SNVs within the deletion, which implied the presence of a third allele [File S_4_Figure1].

File S_4_Figure1. Sanger sequencing confirmation of minor alleles within the deleted interval. Since the region shown is within a confirmed (by Sanger sequencing) deleted interval, the presence of a detected minor allele would also imply the presence of a third haplotype.

Sanger sequencing of SNVs inside the deleted interval confirmed the presence of an alternate allele, albeit not in a 50/50 proportion, as suggested by WGS. [File S_4_Figure2]. The presence of a third haplotype is consistent with mosaicism or chimerism.

File S_4_Figure2. Minor alleles visually detected within the deleted through Sanger sequencing. (A) minor haplotype visible in the highlighted areas. Note the significant differences in the electropherogram peak size.

To check whether this suspected allelic discrepancy was localized or diffused, we carried out a genome-wide site-level screening which identified 1,442 loci in the proband that had a minor-allele fraction ≤ 0.20. Comparison with 36 control dogs called with the same pipeline and with an average coverage of 28x or greater, did not show a generalized excess of skewed sites in the proband, arguing against a genome-wide pattern of allelic imbalance. Visual inspection of the top 5 most skewed sites outside the deleted interval per chromosome indicate that these were attributable to alignment-related artifacts (examples in File S_4_Figure3, list of top 5 per chromosome reported in Supplementary File_S4_Table2).

File S_4_Figure3. Examples of likely artifacts among sites initially flagged as having highly skewed minor-allele fractions. (A) A local alignment artifact in which a column of apparent mismatches is generated near a disrupted alignment region, likely reflecting misplacement of bases around an indel or repetitive/low-complexity sequence rather than a clean SNV. (B) A low-level base-calling or alignment noise artifact, visible as scattered isolated alternate bases in only a small fraction of reads, without consistent haplotypic support.

Two candidate sites did not conform to the artifact patterns described above: a purine transition (A>G) at chr16:58,115,148 and a pyrimidine transition (T>C) at chr21:18,364,492 (File S_4_Figure4).

File S_4_Figure4. (A>G) at chr16:58,115,148 and (T>C) at chr21:18,364,492 transitions used to verify the potential presence of imbalanced minor alleles in other genomic regions.

Primers were designed looking for any asymmetrical peak and therefore possible chimerism outside of the variant.

However, Sanger sequencing of these outside-of-the deletion variants showed heterozygous peaks of comparable size (File S_4_Figure5).

File S_4_Figure5. Electropherograms of the (A) A>G at chr16:58,115,148 and (B) T>C at chr21:18,364,492 variants. Compare these equal size or comparable size heterozygous peaks against the small peaks related to the third allele in File S_4_Figure2.

**Discussion**

The combined results of WGS, Sanger sequencing, and allele-balance analyses suggest that generalized chimerism is unlikely to explain the low-frequency minor allele seen within the deleted region. Instead, mosaicism is the more likely explanation. Although a definitive confirmation is not possible with the available data, other skewed loci outside the candidate deletion appeared to be sequencing/alignment artifacts on IGV review or showed normal balanced heterozygous peaks on Sanger sequencing. Therefore, the findings most strongly support localized mosaicism involving the deleted interval, with the likely persistence of the non-deleted allele in a subset of cells in the proband. This pattern would be possibly consistent with a deletion arising during early embryonic development (post-zygotically) rather than being inherited from the parental gametes.

**File_S4_Table2.** Candidate variants identified during screening of the proband BAM file for skewed allele balance. Each row represents one variant site. “chr” indicates the chromosome and “pos” the genomic coordinate. “ref” and “alt” indicate the reference and alternate allele at that position. “gt” is the genotype call for the sample, where “0/1” indicates a heterozygous call, and “1/1” indicates a homozygous alternate call; “ad” reports the allelic depth as “reference-read” count, “alternate-read” count. “dp” is the total read depth at the site, and “gq” is the genotype quality score. “ref_count” and “alt_count” give the parsed read supporting the counts for reference and alternate alleles, respectively. “minor_count” and “major_count” indicate the counts of the two alleles. “minor_fraction” is calculated as “minor_count” / “dp" and represents the proportion of reads supporting the minor allele.

| ***chr*** | ***pos*** | ***ref*** | ***alt*** | ***gt*** | ***ad*** | ***dp*** | ***gq*** | ***ref_ count*** | ***alt_ count*** | ***minor_ count*** | ***major_ count*** | ***minor_ fraction*** |
| --- | --- | --- | --- | --- | --- | --- | --- | --- | --- | --- | --- | --- |
| chr1 | 28891671 | T | G | 1/1 | 2,21 | 23 | 54 | 2 | 21 | 2 | 21 | 0.08696 |
| chr1 | 81220135 | T | A | 1/1 | 2,22 | 24 | 52 | 2 | 22 | 2 | 22 | 0.08333 |
| chr1 | 90827835 | C | G | 1/1 | 2,25 | 27 | 28 | 2 | 25 | 2 | 25 | 0.07407 |
| chr1 | 111305166 | T | A | 0/1 | 17,2 | 19 | 39 | 17 | 2 | 2 | 17 | 0.10526 |
| chr1 | 117706403 | A | T | 1/1 | 2,30 | 32 | 85 | 2 | 30 | 2 | 30 | 0.0625 |
| chr2 | 7683556 | T | A | 1/1 | 2,21 | 23 | 48 | 2 | 21 | 2 | 21 | 0.08696 |
| chr2 | 23611759 | G | T | 1/1 | 2,17 | 19 | 37 | 2 | 17 | 2 | 17 | 0.10526 |
| chr2 | 36303462 | T | C | 0/1 | 16,2 | 18 | 39 | 16 | 2 | 2 | 16 | 0.11111 |
| chr2 | 46276426 | T | A | 0/1 | 2,18 | 20 | 30 | 2 | 18 | 2 | 18 | 0.1 |
| chr2 | 84501208 | A | T | 1/1 | 2,30 | 32 | 81 | 2 | 30 | 2 | 30 | 0.0625 |
| chr3 | 21354859 | A | C | 1/1 | 2,22 | 24 | 51 | 2 | 22 | 2 | 22 | 0.08333 |
| chr3 | 40453808 | A | G | 0/1 | 17,2 | 19 | 42 | 17 | 2 | 2 | 17 | 0.10526 |
| chr3 | 69171945 | T | A | 1/1 | 2,25 | 27 | 24 | 2 | 25 | 2 | 25 | 0.07407 |
| chr3 | 85798362 | T | C | 0/1 | 25,3 | 28 | 51 | 25 | 3 | 3 | 25 | 0.10714 |
| chr3 | 91122995 | T | A | 0/1 | 22,2 | 24 | 42 | 22 | 2 | 2 | 22 | 0.08333 |
| chr4 | 4856470 | T | G | 0/1 | 24,3 | 27 | 78 | 24 | 3 | 3 | 24 | 0.11111 |
| chr4 | 4856508 | T | G | 0/1 | 28,3 | 31 | 75 | 28 | 3 | 3 | 28 | 0.09677 |
| chr4 | 23756711 | C | G | 1/1 | 3,26 | 29 | 72 | 3 | 26 | 3 | 26 | 0.10345 |
| chr4 | 33716620 | G | T | 0/1 | 25,3 | 28 | 41 | 25 | 3 | 3 | 25 | 0.10714 |
| chr4 | 37652687 | C | G | 1/1 | 2,21 | 23 | 37 | 2 | 21 | 2 | 21 | 0.08696 |
| chr5 | 15135081 | A | C | 1/1 | 2,26 | 28 | 33 | 2 | 26 | 2 | 26 | 0.07143 |
| chr5 | 26830828 | G | A | 0/1 | 18,2 | 20 | 39 | 18 | 2 | 2 | 18 | 0.1 |
| chr5 | 50455182 | T | G | 1/1 | 2,28 | 30 | 27 | 2 | 28 | 2 | 28 | 0.06667 |
| chr5 | 76303452 | G | T | 1/1 | 2,26 | 28 | 74 | 2 | 26 | 2 | 26 | 0.07143 |
| chr5 | 85796663 | A | T | 1/1 | 2,31 | 33 | 43 | 2 | 31 | 2 | 31 | 0.06061 |
| chr6 | 14139817 | T | C | 0/1 | 25,3 | 28 | 51 | 25 | 3 | 3 | 25 | 0.10714 |
| chr6 | 22591345 | A | C | 1/1 | 2,31 | 33 | 62 | 2 | 31 | 2 | 31 | 0.06061 |
| chr6 | 55219312 | G | C | 1/1 | 2,19 | 21 | 34 | 2 | 19 | 2 | 19 | 0.09524 |
| chr6 | 71305991 | C | G | 1/1 | 2,21 | 23 | 28 | 2 | 21 | 2 | 21 | 0.08696 |
| chr6 | 75070442 | C | T | 1/1 | 3,29 | 32 | 74 | 3 | 29 | 3 | 29 | 0.09375 |
| chr7 | 11039191 | A | T | 0/1 | 27,3 | 30 | 45 | 27 | 3 | 3 | 27 | 0.1 |
| chr7 | 17000563 | C | T | 0/1 | 2,19 | 21 | 27 | 2 | 19 | 2 | 19 | 0.09524 |
| chr7 | 24400296 | A | G | 0/1 | 26,3 | 29 | 63 | 26 | 3 | 3 | 26 | 0.10345 |
| chr7 | 24400301 | A | G | 0/1 | 26,3 | 29 | 63 | 26 | 3 | 3 | 26 | 0.10345 |
| chr7 | 28480056 | G | A | 0/1 | 28,3 | 31 | 42 | 28 | 3 | 3 | 28 | 0.09677 |
| chr8 | 6854786 | C | G | 1/1 | 2,27 | 29 | 38 | 2 | 27 | 2 | 27 | 0.06897 |
| chr8 | 24149027 | A | C | 0/1 | 2,20 | 22 | 27 | 2 | 20 | 2 | 20 | 0.09091 |
| chr8 | 24893078 | T | G | 1/1 | 2,23 | 25 | 24 | 2 | 23 | 2 | 23 | 0.08 |
| chr8 | 74770817 | T | A | 0/1 | 2,20 | 22 | 24 | 2 | 20 | 2 | 20 | 0.09091 |
| chr8 | 76186053 | T | G | 0/1 | 21,2 | 23 | 48 | 21 | 2 | 2 | 21 | 0.08696 |
| chr9 | 3937027 | C | G | 0/1 | 2,17 | 19 | 20 | 2 | 17 | 2 | 17 | 0.10526 |
| chr9 | 10215895 | T | A | 0/1 | 25,3 | 28 | 43 | 25 | 3 | 3 | 25 | 0.10714 |
| chr9 | 33565814 | A | T | 1/1 | 2,17 | 19 | 38 | 2 | 17 | 2 | 17 | 0.10526 |
| chr9 | 37096067 | C | G | 0/1 | 16,2 | 18 | 39 | 16 | 2 | 2 | 16 | 0.11111 |
| chr9 | 52560400 | C | G | 0/1 | 2,16 | 18 | 21 | 2 | 16 | 2 | 16 | 0.11111 |
| chr10 | 20047435 | C | A | 0/1 | 2,17 | 19 | 33 | 2 | 17 | 2 | 17 | 0.10526 |
| chr10 | 25806951 | G | T | 1/1 | 2,19 | 21 | 39 | 2 | 19 | 2 | 19 | 0.09524 |
| chr10 | 28512766 | T | G | 1/1 | 2,18 | 20 | 40 | 2 | 18 | 2 | 18 | 0.1 |
| chr10 | 48599156 | A | C | 1/1 | 2,30 | 32 | 61 | 2 | 30 | 2 | 30 | 0.0625 |
| chr10 | 61770322 | T | G | 1/1 | 2,21 | 23 | 52 | 2 | 21 | 2 | 21 | 0.08696 |
| chr11 | 34567995 | T | A | 1/1 | 3,31 | 34 | 58 | 3 | 31 | 3 | 31 | 0.08824 |
| chr11 | 38777411 | T | G | 0/1 | 18,2 | 20 | 48 | 18 | 2 | 2 | 18 | 0.1 |
| chr11 | 38790557 | G | T | 0/1 | 22,2 | 24 | 38 | 22 | 2 | 2 | 22 | 0.08333 |
| chr11 | 39315345 | T | A | 1/1 | 2,17 | 19 | 37 | 2 | 17 | 2 | 17 | 0.10526 |
| chr11 | 69567267 | A | T | 1/1 | 2,27 | 29 | 27 | 2 | 27 | 2 | 27 | 0.06897 |
| chr12 | 937631 | T | G | 1/1 | 2,20 | 22 | 29 | 2 | 20 | 2 | 20 | 0.09091 |
| chr12 | 16215676 | T | A | 0/1 | 26,3 | 29 | 48 | 26 | 3 | 3 | 26 | 0.10345 |
| chr12 | 18199012 | C | G | 1/1 | 2,30 | 32 | 84 | 2 | 30 | 2 | 30 | 0.0625 |
| chr12 | 21371453 | C | A | 0/1 | 22,2 | 24 | 39 | 22 | 2 | 2 | 22 | 0.08333 |
| chr12 | 21371454 | C | A | 0/1 | 22,2 | 24 | 39 | 22 | 2 | 2 | 22 | 0.08333 |
| chr13 | 27532453 | C | G | 1/1 | 2,21 | 23 | 20 | 2 | 21 | 2 | 21 | 0.08696 |
| chr13 | 38979963 | C | T | 1/1 | 2,31 | 33 | 76 | 2 | 31 | 2 | 31 | 0.06061 |
| chr13 | 40092140 | G | A | 0/1 | 2,21 | 23 | 21 | 2 | 21 | 2 | 21 | 0.08696 |
| chr13 | 48765022 | A | C | 1/1 | 2,31 | 33 | 22 | 2 | 31 | 2 | 31 | 0.06061 |
| chr13 | 63196267 | T | A | 1/1 | 2,28 | 30 | 56 | 2 | 28 | 2 | 28 | 0.06667 |
| chr14 | 2421354 | C | G | 1/1 | 2,24 | 26 | 23 | 2 | 24 | 2 | 24 | 0.07692 |
| chr14 | 12171004 | A | G | 0/1 | 16,2 | 18 | 45 | 16 | 2 | 2 | 16 | 0.11111 |
| chr14 | 35279754 | C | G | 0/1 | 2,20 | 22 | 25 | 2 | 20 | 2 | 20 | 0.09091 |
| chr14 | 41121109 | A | C | 0/1 | 28,3 | 31 | 60 | 28 | 3 | 3 | 28 | 0.09677 |
| chr14 | 50527826 | A | G | 0/1 | 24,2 | 26 | 39 | 24 | 2 | 2 | 24 | 0.07692 |
| chr15 | 5419805 | G | T | 0/1 | 2,21 | 23 | 21 | 2 | 21 | 2 | 21 | 0.08696 |
| chr15 | 20360310 | G | A | 1/1 | 2,25 | 27 | 31 | 2 | 25 | 2 | 25 | 0.07407 |
| chr15 | 42145659 | A | T | 0/1 | 19,2 | 21 | 45 | 19 | 2 | 2 | 19 | 0.09524 |
| chr15 | 49886197 | G | T | 0/1 | 2,18 | 20 | 30 | 2 | 18 | 2 | 18 | 0.1 |
| chr15 | 58167538 | A | T | 0/1 | 2,17 | 19 | 33 | 2 | 17 | 2 | 17 | 0.10526 |
| chr16 | 5685150 | T | G | 0/1 | 25,3 | 28 | 54 | 25 | 3 | 3 | 25 | 0.10714 |
| chr16 | 5685153 | T | G | 0/1 | 25,3 | 28 | 54 | 25 | 3 | 3 | 25 | 0.10714 |
| chr16 | 9064705 | G | A | 1/1 | 2,24 | 26 | 40 | 2 | 24 | 2 | 24 | 0.07692 |
| chr16 | 32130597 | C | T | 0/1 | 2,17 | 19 | 26 | 2 | 17 | 2 | 17 | 0.10526 |
| chr16 | 47592943 | T | A | 0/1 | 2,18 | 20 | 23 | 2 | 18 | 2 | 18 | 0.1 |
| chr17 | 9828805 | T | G | 0/1 | 26,3 | 29 | 48 | 26 | 3 | 3 | 26 | 0.10345 |
| chr17 | 9828810 | C | T | 0/1 | 26,3 | 29 | 48 | 26 | 3 | 3 | 26 | 0.10345 |
| chr17 | 35653688 | A | C | 1/1 | 2,20 | 22 | 45 | 2 | 20 | 2 | 20 | 0.09091 |
| chr17 | 64385099 | C | G | 0/1 | 24,3 | 27 | 54 | 24 | 3 | 3 | 24 | 0.11111 |
| chr17 | 64385100 | C | G | 0/1 | 24,3 | 27 | 54 | 24 | 3 | 3 | 24 | 0.11111 |
| chr18 | 78035 | A | G | 0/1 | 30,3 | 33 | 48 | 30 | 3 | 3 | 30 | 0.09091 |
| chr18 | 880386 | C | A | 0/1 | 19,2 | 21 | 42 | 19 | 2 | 2 | 19 | 0.09524 |
| chr18 | 15563651 | G | C | 1/1 | 2,18 | 20 | 43 | 2 | 18 | 2 | 18 | 0.1 |
| chr18 | 30235107 | A | C | 0/1 | 2,20 | 22 | 24 | 2 | 20 | 2 | 20 | 0.09091 |
| chr18 | 31905771 | T | G | 0/1 | 21,2 | 23 | 42 | 21 | 2 | 2 | 21 | 0.08696 |
| chr19 | 504099 | C | G | 0/1 | 25,3 | 28 | 51 | 25 | 3 | 3 | 25 | 0.10714 |
| chr19 | 9566864 | T | A | 0/1 | 2,17 | 19 | 32 | 2 | 17 | 2 | 17 | 0.10526 |
| chr19 | 33492513 | C | T | 1/1 | 2,18 | 20 | 52 | 2 | 18 | 2 | 18 | 0.1 |
| chr19 | 55515983 | G | A | 0/1 | 19,2 | 21 | 39 | 19 | 2 | 2 | 19 | 0.09524 |
| chr19 | 55516019 | C | T | 0/1 | 18,2 | 20 | 39 | 18 | 2 | 2 | 18 | 0.1 |
| chr20 | 17747696 | T | C | 0/1 | 2,17 | 19 | 33 | 2 | 17 | 2 | 17 | 0.10526 |
| chr20 | 18343710 | T | A | 1/1 | 2,29 | 31 | 36 | 2 | 29 | 2 | 29 | 0.06452 |
| chr20 | 20371800 | C | G | 1/1 | 2,19 | 21 | 33 | 2 | 19 | 2 | 19 | 0.09524 |
| chr20 | 29908167 | T | A | 1/1 | 2,23 | 25 | 66 | 2 | 23 | 2 | 23 | 0.08 |
| chr20 | 36510711 | T | A | 0/1 | 25,3 | 28 | 41 | 25 | 3 | 3 | 25 | 0.10714 |
| chr21 | 18128233 | A | G | 0/1 | 21,2 | 23 | 45 | 21 | 2 | 2 | 21 | 0.08696 |
| chr21 | 22002175 | G | C | 1/1 | 3,32 | 35 | 77 | 3 | 32 | 3 | 32 | 0.08571 |
| chr21 | 22561651 | G | C | 0/1 | 15,2 | 17 | 39 | 15 | 2 | 2 | 15 | 0.11765 |
| chr21 | 31475232 | A | G | 0/1 | 16,2 | 18 | 45 | 16 | 2 | 2 | 16 | 0.11111 |
| chr21 | 47732485 | T | G | 1/1 | 2,21 | 23 | 47 | 2 | 21 | 2 | 21 | 0.08696 |
| chr22 | 12826828 | C | G | 0/1 | 26,3 | 29 | 66 | 26 | 3 | 3 | 26 | 0.10345 |
| chr22 | 32653390 | G | C | 0/1 | 24,3 | 27 | 54 | 24 | 3 | 3 | 24 | 0.11111 |
| chr22 | 41483075 | T | C | 0/1 | 15,2 | 17 | 39 | 15 | 2 | 2 | 15 | 0.11765 |
| chr22 | 49732782 | C | T | 0/1 | 15,2 | 17 | 42 | 15 | 2 | 2 | 15 | 0.11765 |
| chr22 | 49732787 | A | T | 0/1 | 15,2 | 17 | 42 | 15 | 2 | 2 | 15 | 0.11765 |
| chr23 | 207823 | T | C | 0/1 | 15,2 | 17 | 39 | 15 | 2 | 2 | 15 | 0.11765 |
| chr23 | 207824 | A | G | 0/1 | 15,2 | 17 | 39 | 15 | 2 | 2 | 15 | 0.11765 |
| chr23 | 13455071 | C | A | 0/1 | 15,2 | 17 | 39 | 15 | 2 | 2 | 15 | 0.11765 |
| chr23 | 28207941 | T | A | 1/1 | 2,22 | 24 | 32 | 2 | 22 | 2 | 22 | 0.08333 |
| chr23 | 52285827 | T | C | 0/1 | 2,16 | 18 | 30 | 2 | 16 | 2 | 16 | 0.11111 |
| chr24 | 334068 | C | T | 0/1 | 24,3 | 27 | 54 | 24 | 3 | 3 | 24 | 0.11111 |
| chr24 | 1579485 | A | T | 0/1 | 15,2 | 17 | 39 | 15 | 2 | 2 | 15 | 0.11765 |
| chr24 | 14715483 | A | T | 0/1 | 2,16 | 18 | 39 | 2 | 16 | 2 | 16 | 0.11111 |
| chr24 | 41905687 | G | A | 1/1 | 2,17 | 19 | 21 | 2 | 17 | 2 | 17 | 0.10526 |
| chr24 | 44384691 | G | A | 1/1 | 2,17 | 19 | 46 | 2 | 17 | 2 | 17 | 0.10526 |
| chr25 | 34745318 | C | G | 1/1 | 2,24 | 26 | 35 | 2 | 24 | 2 | 24 | 0.07692 |
| chr25 | 36021286 | C | G | 1/1 | 2,21 | 23 | 20 | 2 | 21 | 2 | 21 | 0.08696 |
| chr25 | 43114279 | T | A | 1/1 | 3,32 | 35 | 24 | 3 | 32 | 3 | 32 | 0.08571 |
| chr25 | 47714685 | G | A | 1/1 | 3,24 | 27 | 63 | 3 | 24 | 3 | 24 | 0.11111 |
| chr25 | 48782311 | T | A | 1/1 | 2,26 | 28 | 32 | 2 | 26 | 2 | 26 | 0.07143 |
| chr26 | 2435414 | C | G | 1/1 | 2,20 | 22 | 24 | 2 | 20 | 2 | 20 | 0.09091 |
| chr26 | 2815221 | T | C | 1/1 | 2,19 | 21 | 53 | 2 | 19 | 2 | 19 | 0.09524 |
| chr26 | 6122985 | T | A | 1/1 | 2,23 | 25 | 22 | 2 | 23 | 2 | 23 | 0.08 |
| chr26 | 25582499 | T | C | 0/1 | 28,3 | 31 | 42 | 28 | 3 | 3 | 28 | 0.09677 |
| chr26 | 26705323 | T | G | 0/1 | 27,3 | 30 | 45 | 27 | 3 | 3 | 27 | 0.1 |
| chr27 | 2872208 | T | C | 0/1 | 2,19 | 21 | 28 | 2 | 19 | 2 | 19 | 0.09524 |
| chr27 | 8937717 | T | G | 1/1 | 2,20 | 22 | 43 | 2 | 20 | 2 | 20 | 0.09091 |
| chr27 | 29006177 | T | A | 1/1 | 2,17 | 19 | 21 | 2 | 17 | 2 | 17 | 0.10526 |
| chr27 | 33540968 | T | G | 1/1 | 2,28 | 30 | 68 | 2 | 28 | 2 | 28 | 0.06667 |
| chr27 | 45550045 | T | G | 0/1 | 18,2 | 20 | 42 | 18 | 2 | 2 | 18 | 0.1 |
| chr28 | 4963038 | C | T | 0/1 | 24,3 | 27 | 42 | 24 | 3 | 3 | 24 | 0.11111 |
| chr28 | 13912381 | T | C | 0/1 | 15,2 | 17 | 39 | 15 | 2 | 2 | 15 | 0.11765 |
| chr28 | 14779542 | C | G | 0/1 | 2,17 | 19 | 21 | 2 | 17 | 2 | 17 | 0.10526 |
| chr28 | 15292601 | T | A | 1/1 | 2,17 | 19 | 34 | 2 | 17 | 2 | 17 | 0.10526 |
| chr28 | 22282382 | A | T | 1/1 | 2,23 | 25 | 38 | 2 | 23 | 2 | 23 | 0.08 |
| chr29 | 5543941 | C | T | 0/1 | 27,3 | 30 | 45 | 27 | 3 | 3 | 27 | 0.1 |
| chr29 | 6864240 | T | C | 0/1 | 17,2 | 19 | 48 | 17 | 2 | 2 | 17 | 0.10526 |
| chr29 | 8937813 | A | C | 1/1 | 2,27 | 29 | 70 | 2 | 27 | 2 | 27 | 0.06897 |
| chr29 | 11266353 | A | C | 0/1 | 2,20 | 22 | 27 | 2 | 20 | 2 | 20 | 0.09091 |
| chr29 | 36146886 | A | C | 1/1 | 2,17 | 19 | 21 | 2 | 17 | 2 | 17 | 0.10526 |
| chr30 | 1225700 | G | A | 0/1 | 17,2 | 19 | 48 | 17 | 2 | 2 | 17 | 0.10526 |
| chr30 | 5868718 | A | G | 0/1 | 18,2 | 20 | 45 | 18 | 2 | 2 | 18 | 0.1 |
| chr30 | 5868720 | T | G | 0/1 | 16,2 | 18 | 45 | 16 | 2 | 2 | 16 | 0.11111 |
| chr30 | 8510736 | C | A | 0/1 | 16,2 | 18 | 39 | 16 | 2 | 2 | 16 | 0.11111 |
| chr30 | 38268465 | C | A | 0/1 | 2,21 | 23 | 21 | 2 | 21 | 2 | 21 | 0.08696 |
| chr31 | 1135296 | G | A | 0/1 | 15,2 | 17 | 39 | 15 | 2 | 2 | 15 | 0.11765 |
| chr31 | 10166677 | T | A | 1/1 | 2,28 | 30 | 37 | 2 | 28 | 2 | 28 | 0.06667 |
| chr31 | 26583432 | T | A | 1/1 | 2,21 | 23 | 58 | 2 | 21 | 2 | 21 | 0.08696 |
| chr31 | 27189915 | T | G | 0/1 | 2,16 | 18 | 38 | 2 | 16 | 2 | 16 | 0.11111 |
| chr31 | 36546954 | G | T | 0/1 | 25,3 | 28 | 39 | 25 | 3 | 3 | 25 | 0.10714 |
| chr32 | 483678 | A | T | 0/1 | 16,2 | 18 | 45 | 16 | 2 | 2 | 16 | 0.11111 |
| chr32 | 5128173 | A | G | 0/1 | 30,3 | 33 | 39 | 30 | 3 | 3 | 30 | 0.09091 |
| chr32 | 22641145 | A | C | 1/1 | 2,17 | 19 | 34 | 2 | 17 | 2 | 17 | 0.10526 |
| chr32 | 24532171 | G | C | 0/1 | 2,16 | 18 | 36 | 2 | 16 | 2 | 16 | 0.11111 |
| chr32 | 39347472 | G | A | 0/1 | 16,2 | 18 | 42 | 16 | 2 | 2 | 16 | 0.11111 |
| chr33 | 8605587 | A | T | 0/1 | 24,3 | 27 | 41 | 24 | 3 | 3 | 24 | 0.11111 |
| chr33 | 11447713 | A | G | 0/1 | 2,15 | 17 | 39 | 2 | 15 | 2 | 15 | 0.11765 |
| chr33 | 26270605 | T | C | 1/1 | 2,31 | 33 | 49 | 2 | 31 | 2 | 31 | 0.06061 |
| chr33 | 27254032 | A | C | 0/1 | 2,16 | 18 | 28 | 2 | 16 | 2 | 16 | 0.11111 |
| chr33 | 29276570 | T | G | 0/1 | 19,2 | 21 | 39 | 19 | 2 | 2 | 19 | 0.09524 |
| chr34 | 4612453 | T | C | 1/1 | 2,16 | 18 | 41 | 2 | 16 | 2 | 16 | 0.11111 |
| chr34 | 10305031 | A | C | 0/1 | 27,3 | 30 | 47 | 27 | 3 | 3 | 27 | 0.1 |
| chr34 | 16066556 | A | T | 0/1 | 24,3 | 27 | 54 | 24 | 3 | 3 | 24 | 0.11111 |
| chr34 | 16066560 | A | G | 0/1 | 24,3 | 27 | 54 | 24 | 3 | 3 | 24 | 0.11111 |
| chr34 | 17565063 | C | G | 0/1 | 2,17 | 19 | 20 | 2 | 17 | 2 | 17 | 0.10526 |
| chr35 | 639526 | T | C | 0/1 | 26,3 | 29 | 48 | 26 | 3 | 3 | 26 | 0.10345 |
| chr35 | 1277988 | G | C | 0/1 | 15,2 | 17 | 39 | 15 | 2 | 2 | 15 | 0.11765 |
| chr35 | 18553858 | A | T | 0/1 | 15,2 | 17 | 39 | 15 | 2 | 2 | 15 | 0.11765 |
| chr35 | 18553861 | C | G | 0/1 | 15,2 | 17 | 39 | 15 | 2 | 2 | 15 | 0.11765 |
| chr35 | 21862213 | G | A | 0/1 | 23,3 | 26 | 66 | 23 | 3 | 3 | 23 | 0.11539 |
| chr36 | 4393789 | T | A | 1/1 | 2,21 | 23 | 20 | 2 | 21 | 2 | 21 | 0.08696 |
| chr36 | 8458085 | C | G | 1/1 | 2,18 | 20 | 21 | 2 | 18 | 2 | 18 | 0.1 |
| chr36 | 23011932 | A | G | 0/1 | 27,3 | 30 | 45 | 27 | 3 | 3 | 27 | 0.1 |
| chr36 | 23864086 | C | T | 1/1 | 3,24 | 27 | 59 | 3 | 24 | 3 | 24 | 0.11111 |
| chr36 | 24506307 | T | A | 1/1 | 2,16 | 18 | 34 | 2 | 16 | 2 | 16 | 0.11111 |
| chr37 | 11230847 | A | T | 0/1 | 14,2 | 16 | 42 | 14 | 2 | 2 | 14 | 0.125 |
| chr37 | 16846267 | A | T | 0/1 | 2,16 | 18 | 22 | 2 | 16 | 2 | 16 | 0.11111 |
| chr37 | 19399088 | T | A | 0/1 | 16,2 | 18 | 45 | 16 | 2 | 2 | 16 | 0.11111 |
| chr37 | 20724899 | C | T | 0/1 | 22,3 | 25 | 60 | 22 | 3 | 3 | 22 | 0.12 |
| chr37 | 22625566 | A | C | 1/1 | 3,20 | 23 | 36 | 3 | 20 | 3 | 20 | 0.13044 |
| chr38 | 68242 | C | A | 0/1 | 28,3 | 31 | 48 | 28 | 3 | 3 | 28 | 0.09677 |
| chr38 | 8457223 | C | G | 0/1 | 16,2 | 18 | 42 | 16 | 2 | 2 | 16 | 0.11111 |
| chr38 | 10445118 | G | A | 0/1 | 15,2 | 17 | 39 | 15 | 2 | 2 | 15 | 0.11765 |
| chr38 | 11413580 | T | A | 0/1 | 15,2 | 17 | 39 | 15 | 2 | 2 | 15 | 0.11765 |
| chr38 | 11413581 | C | T | 0/1 | 15,2 | 17 | 39 | 15 | 2 | 2 | 15 | 0.11765 |
| chrX | 74985476 | C | G | 0/1 | 28,3 | 31 | 42 | 28 | 3 | 3 | 28 | 0.09677 |
| chrX | 106809641 | A | C | 0/1 | 19,2 | 21 | 42 | 19 | 2 | 2 | 19 | 0.09524 |
| chrX | 106809642 | G | A | 0/1 | 19,2 | 21 | 42 | 19 | 2 | 2 | 19 | 0.09524 |
| chrX | 119411505 | A | C | 0/1 | 27,3 | 30 | 45 | 27 | 3 | 3 | 27 | 0.1 |
| chrX | 119411506 | G | A | 0/1 | 27,3 | 30 | 45 | 27 | 3 | 3 | 27 | 0.1 |
