# Supplementary material for: Clinical and molecular genetic description of a Marfan-like syndrome in a Golden Retriever puppy
Source: J Vet Intern Med. 2026 Sep 30;40(5):aalag222. doi: 10.1093/jvimsj/aalag222 (PMC13626598; doi:10.1093/jvimsj/aalag222)
Supplement: Supplementary file 1 [file supplementary_material_aalag222.zip › File S_6.docx]

**Supplementary File 5**: Electropherogram of patient’s FBN1 featuring the deletion, showing the point of the deletion with blue and red arrows pointing towards the 5’ and 3’ prime ends of DNA, respectively. The numbers represent the base pair coordinates of which the sequence stops/resumes on either side of the deletion.
