# Supplementary material for: Clinical and molecular genetic description of a Marfan-like syndrome in a Golden Retriever puppy
Source: J Vet Intern Med. 2026 Sep 30;40(5):aalag222. doi: 10.1093/jvimsj/aalag222 (PMC13626598; doi:10.1093/jvimsj/aalag222)
Supplement: Supplementary file 1 [file supplementary_material_aalag222.zip › File S_7.pdf]

FBN1_F1/F2/R PCR results – golden ret. samples only.

# of samples tested= 153

Testing done 08/14/2025-08/21/2025.

DNA isolation of some samples tested done 08/15/2025. The rest of the samples were isolated prior to the testing.

1.2% agarose gel used in gel electrophoresis.

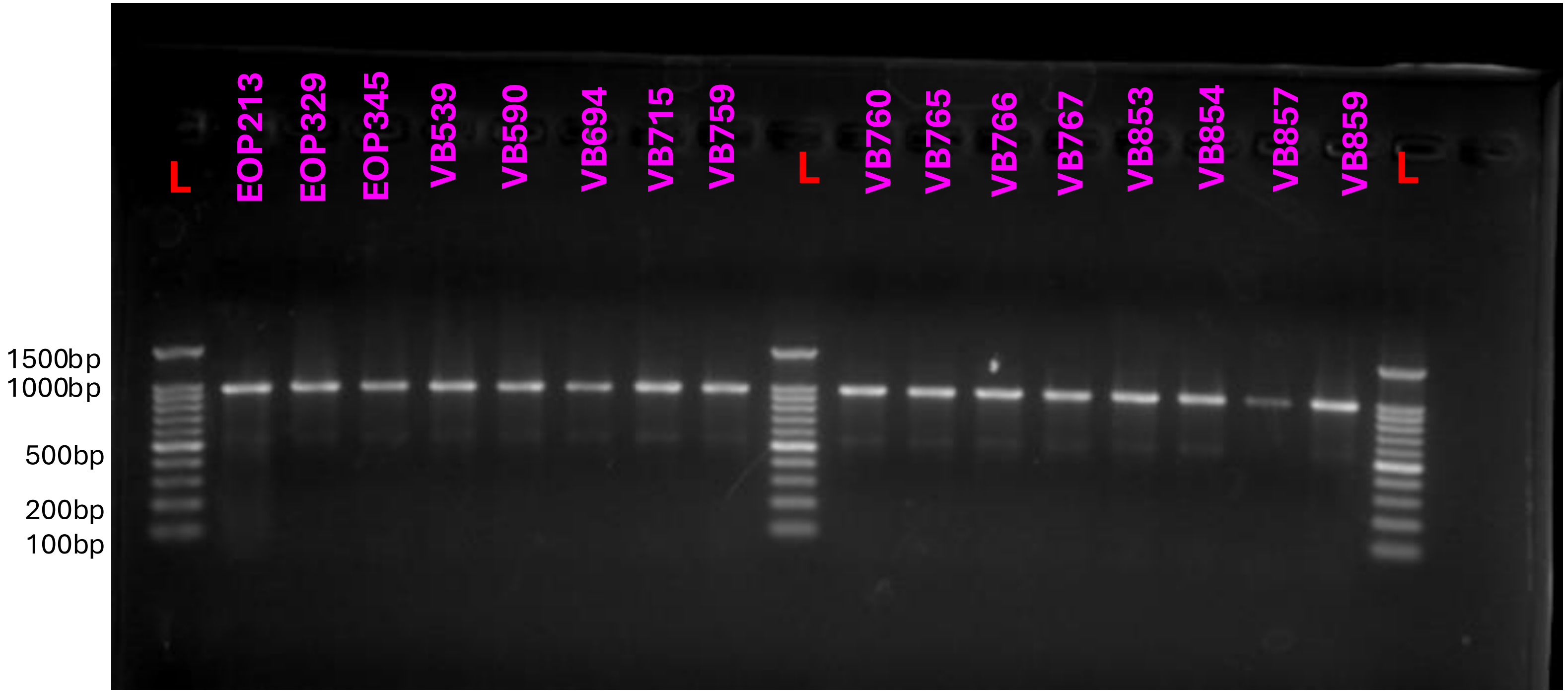

L- 100 bp marker

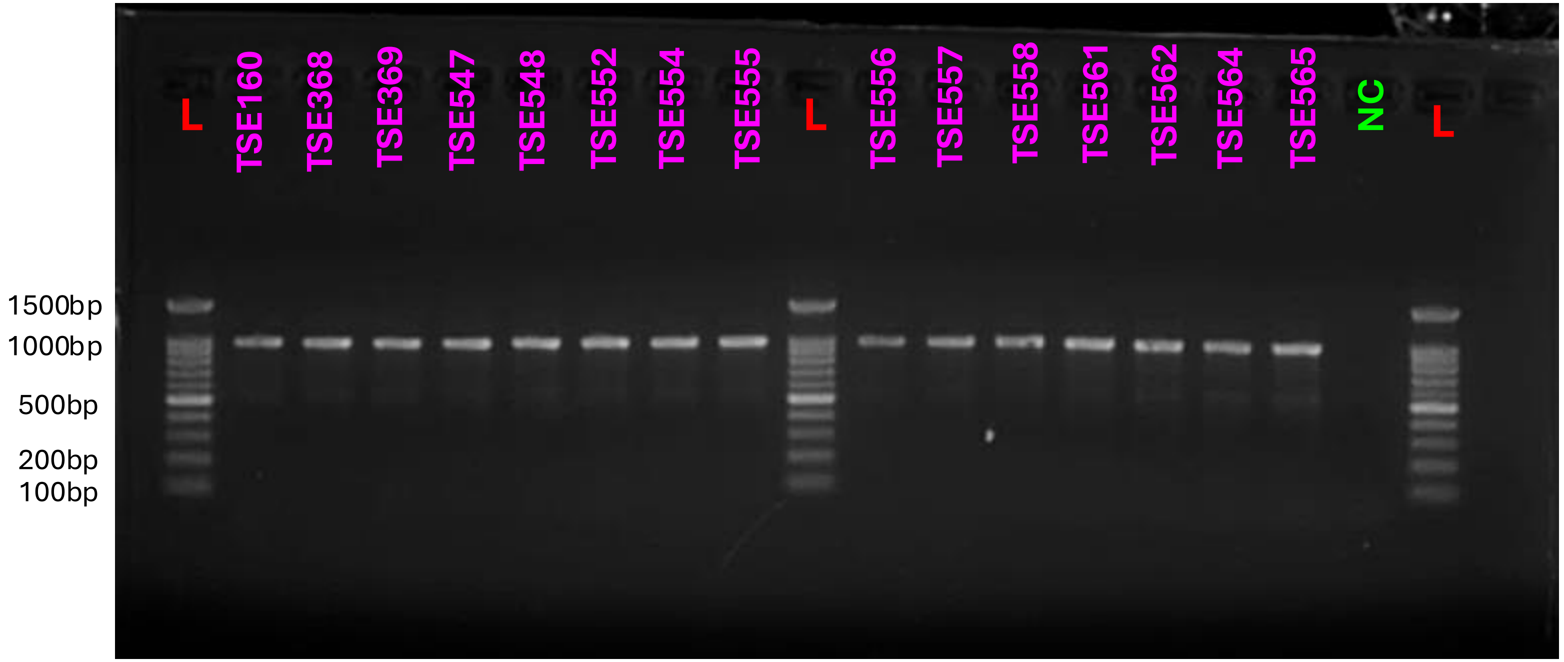

L- 100 bp marker
NC- non template control

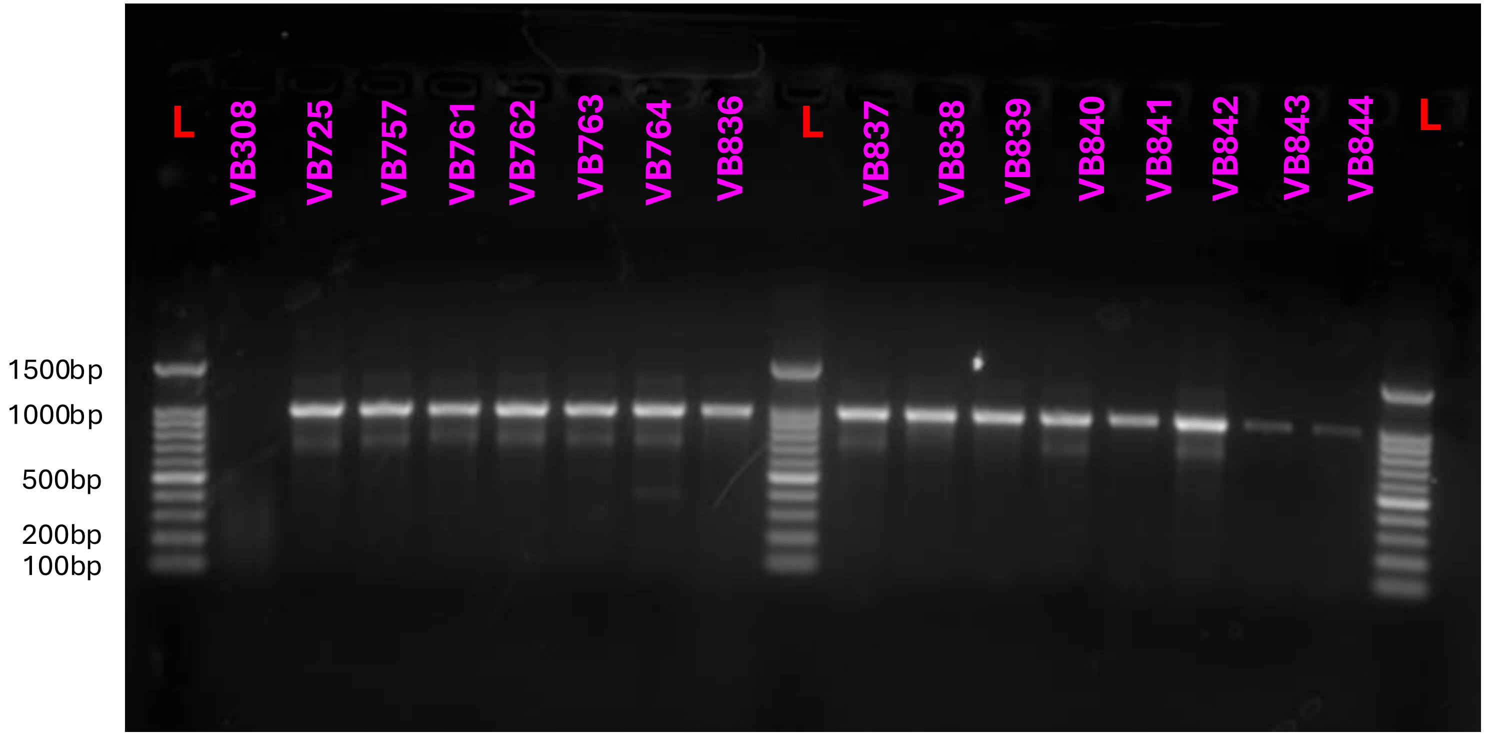

L- 100 bp marker

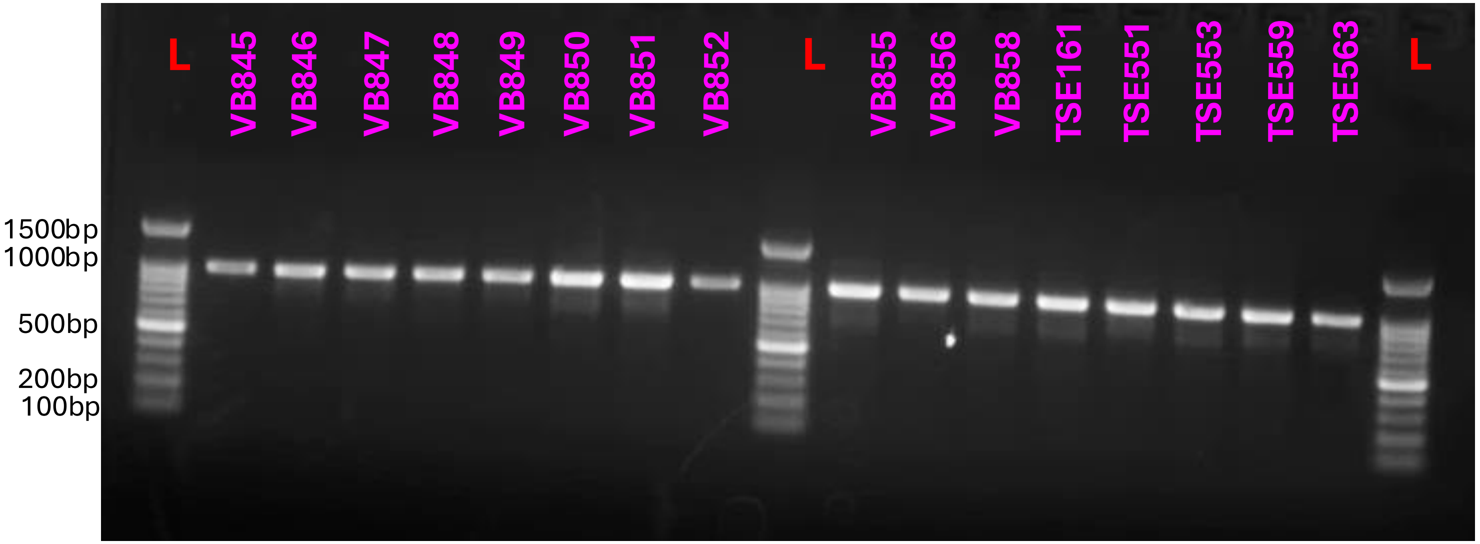

L- 100 bp marker

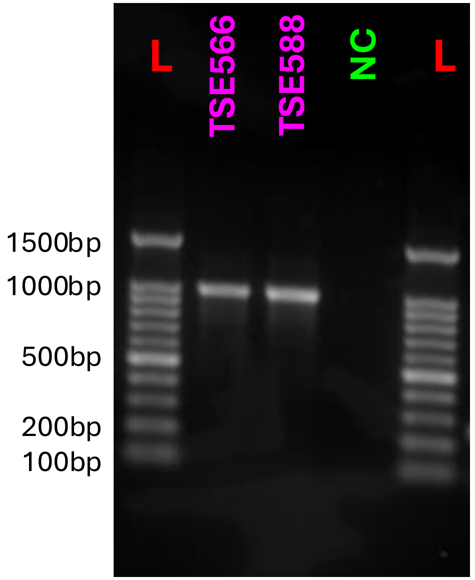

L- 100 bp marker
NC- non template control

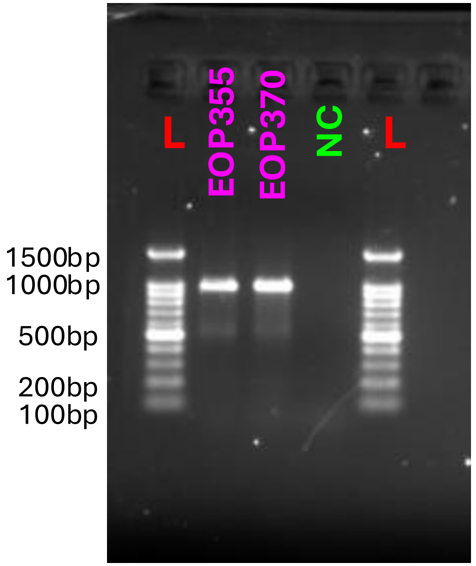

L- 100 bp marker
NC- non template control

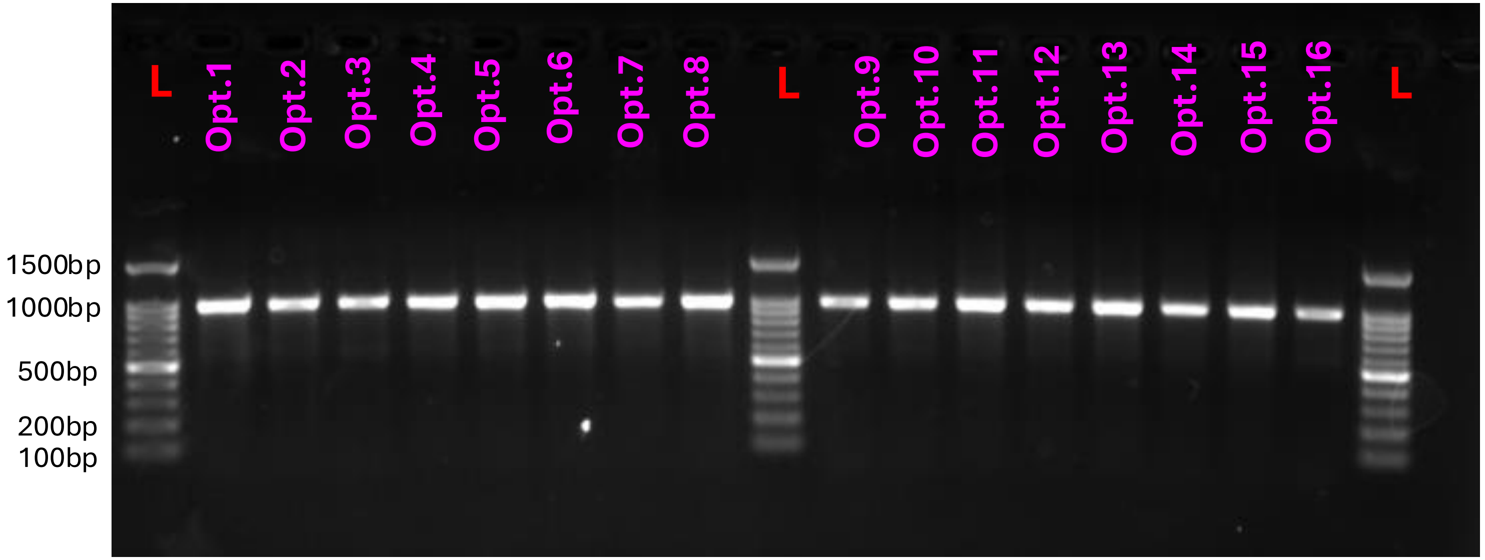

L- 100 bp marker

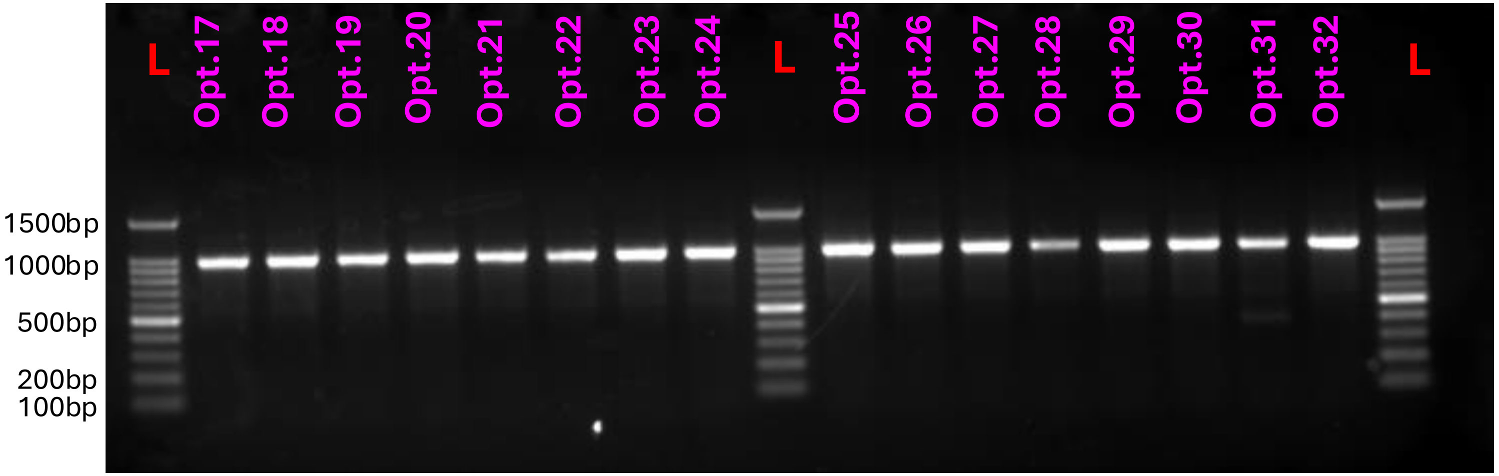

L- 100 bp marker

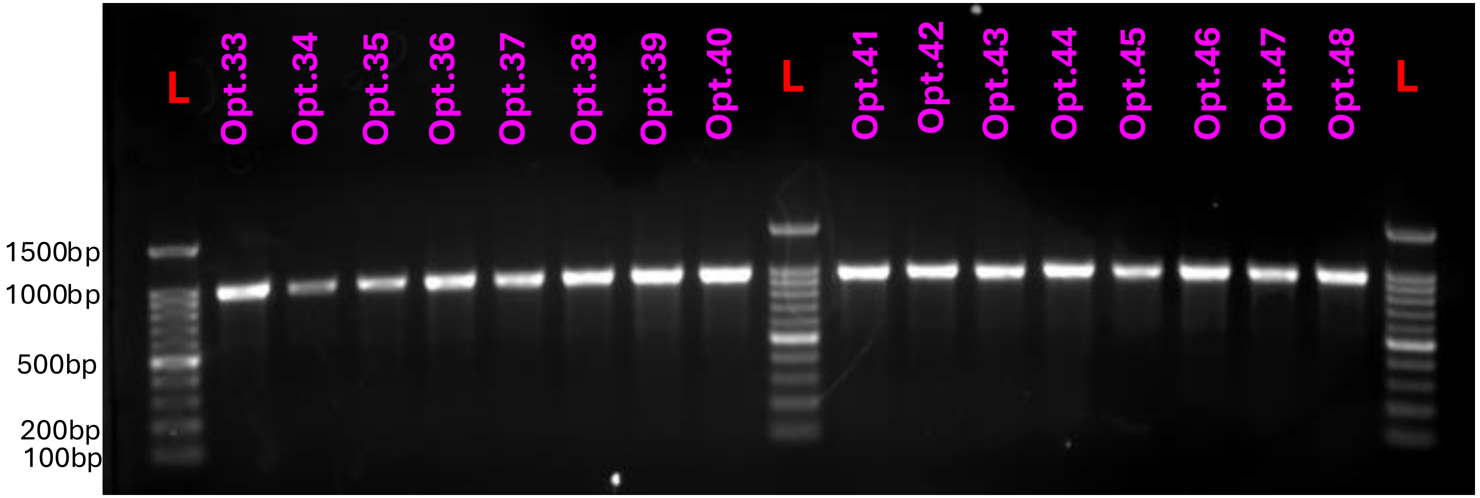

L- 100 bp marker

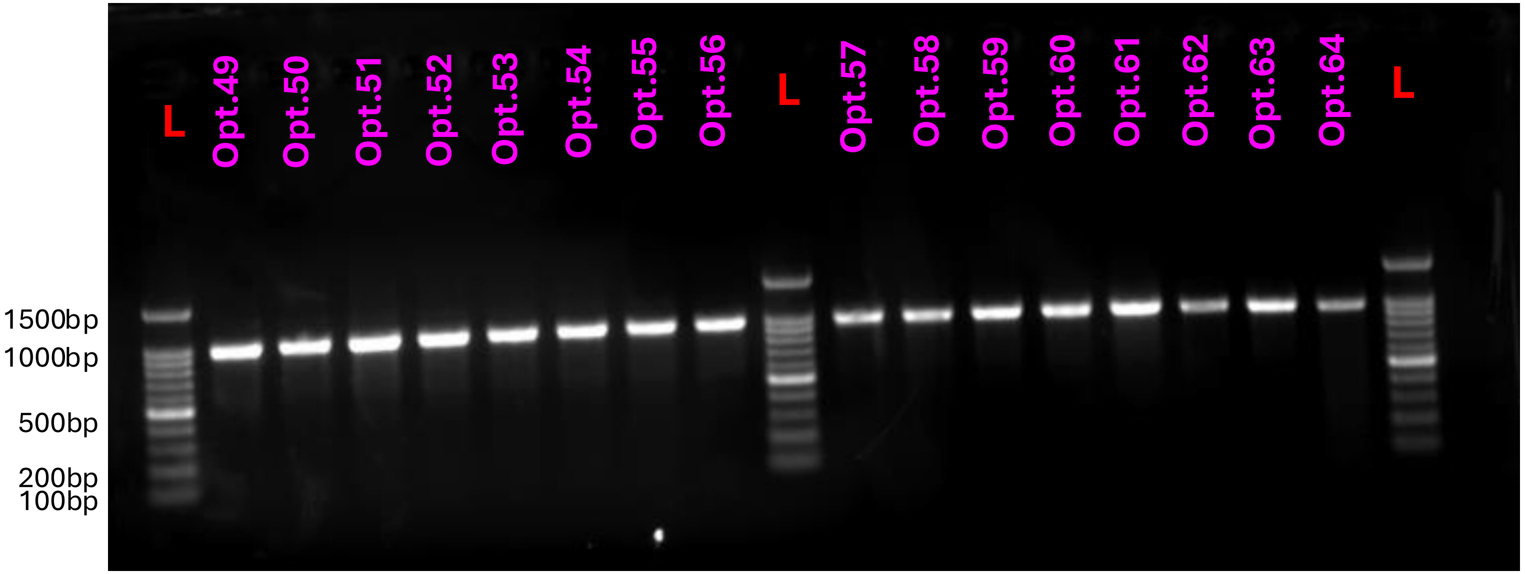

L- 100 bp marker

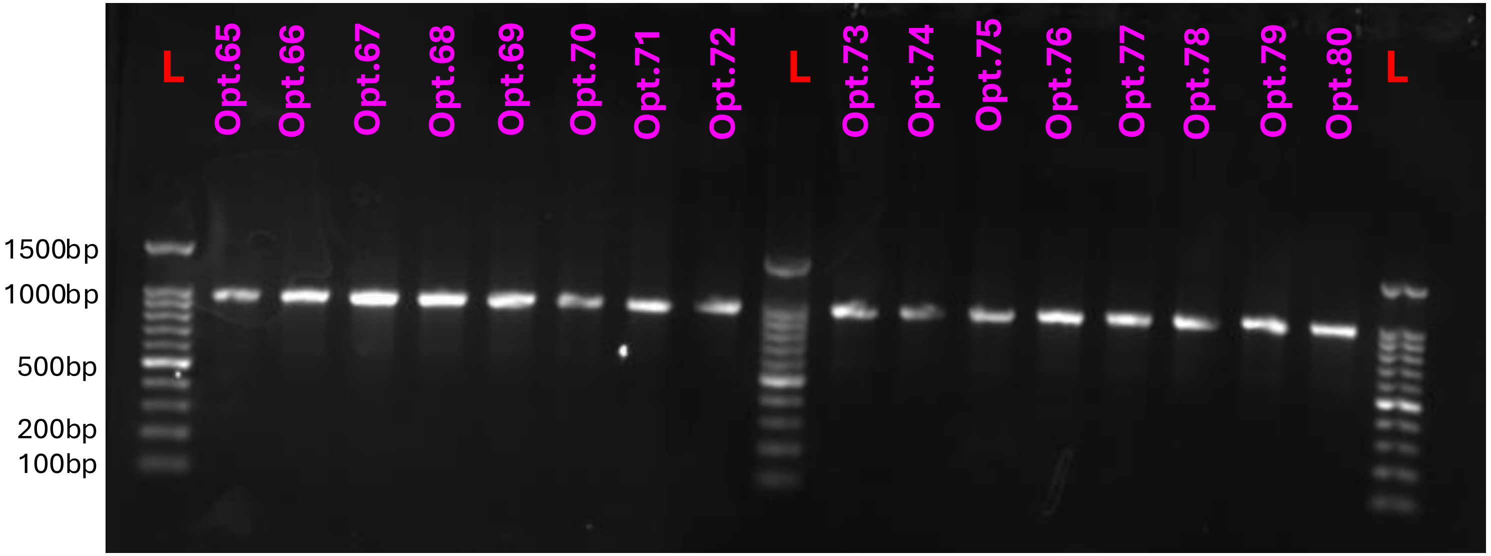

L- 100 bp marker

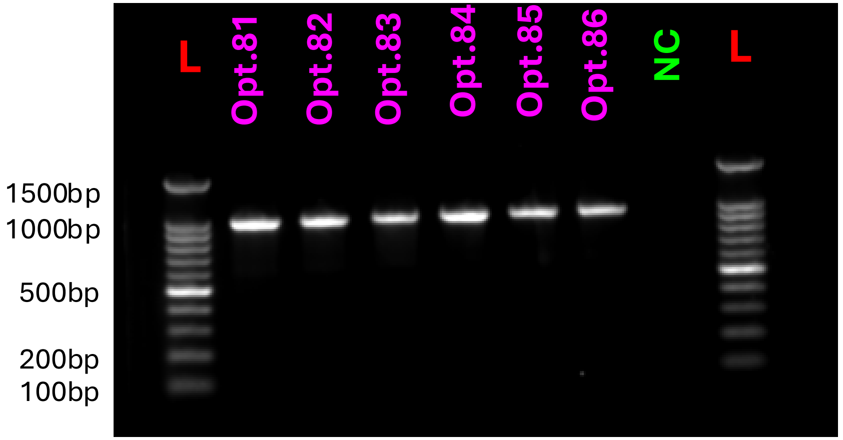

L- 100 bp marker

NC- non template control
