# Supplementary material for: Clinical and molecular genetic description of a Marfan-like syndrome in a Golden Retriever puppy
Source: J Vet Intern Med. 2026 Sep 30;40(5):aalag222. doi: 10.1093/jvimsj/aalag222 (PMC13626598; doi:10.1093/jvimsj/aalag222)
Supplement: Supplementary file 1 [file supplementary_material_aalag222.zip › File S_1.docx]

**Supplementary File 1**: Specific PCR mastermix and cycling parameters.

Mastermix solution (can be scaled):

| 10X *Taq* Buffer | 50 µL |
| --- | --- |
| 2 mM each dNTP | 50 µL |
| Forward 1 | 10 µL |
| Forward 2 | 10 µL |
| Reverse | 10 µL |
| 50 mM MgCl_2_ | 10 µL |
| *Taq* polymerase | 2.5 µL |
| Nuclease-free water | 347.5 µL |

PCR Cycling Parameters

| Initial Denaturation | 95 ºC | 2 minutes | 1 cycle |
| --- | --- | --- | --- |
| Denaturation | 95 ºC | 30 seconds | 38 cycles |
| Annealing | 57 ºC | 30 seconds |  |
| Extension | 72 ºC | 1.5 minutes |  |
| Final Extension | 72 ºC | 5 minutes | 1 cycle |

DNA from a dog with normal clinical features identified as M3246 was used as a wildtype control, and nuclease-free water as a negative control. 1 µL of 1/3 concentration DNA sample was added to mastermix for a 50 µL reaction volume. PCR products were visualized on a 1X TAE 1% agarose gel containing ethidium bromide.
