# Supplementary material for: Clinical and molecular genetic description of a Marfan-like syndrome in a Golden Retriever puppy
Source: J Vet Intern Med. 2026 Sep 30;40(5):aalag222. doi: 10.1093/jvimsj/aalag222 (PMC13626598; doi:10.1093/jvimsj/aalag222)
Supplement: Supplementary file 1 [file supplementary_material_aalag222.zip › File S_2.docx]

**Supplementary File 2**: Transverse captures from the dog’s CT scan, with views going from cranial to caudal as the images go from left to right. A) demonstrates right lung hyperinflation, as evidenced by its relative size to the left lung lobe. B) demonstrates the continuously enlarged nature of the right lung, suggesting displacement secondary to the right middle lung lobe emphysema. C) demonstrates the persistently present right caudal lung lobe, while the left caudal lung lobe has ended at this caudal point of the thorax, and abdominal structures such as the kidney have become present.
